# Safety and efficacy of fruquintinib in patients with previously treated metastatic colorectal cancer: a phase Ib study and a randomized double-blind phase II study

**DOI:** 10.1186/s13045-016-0384-9

**Published:** 2017-01-19

**Authors:** Rui-Hua Xu, Jin Li, Yuxian Bai, Jianming Xu, Tianshu Liu, Lin Shen, Liwei Wang, Hongming Pan, Junning Cao, Dongsheng Zhang, Songhua Fan, Ye Hua, Weiguo Su

**Affiliations:** 1 Department of Medical Oncology, Sun Yat-sen University Cancer Center, State Key Laboratory of Oncology in South China, Collaborative Innovation Center for Cancer Medicine, Guangzhou, 510060 China; 2 Department of Medical Oncology, Fudan University Shanghai Cancer Center, Shanghai Medical College, Shanghai, 200032 China; 3 Department of Medical Oncology, Harbin Medical University Cancer Hospital, Harbin, 150081 China; 4 Department of Medical Oncology, 307th Hospital of PLA, Academy of Military Medical Sciences, Beijing, 100071 China; 5 Department of Medical Oncology, Fudan University Zhongshan Hospital, Shanghai Medical College, Shanghai, 200032 China; 6 Department of Medical Oncology, Beijing Cancer Hospital, Beijing, 100142 China; 7 Department of Medical Oncology, Shanghai First People’s Hospital, Shanghai, 200090 China; 8 Department of Medical Oncology, Sir Run Run Shaw Hospital, School of Medicine, Zhejiang University, Hangzhou, 310016 China; 9 Hutchison MediPharma Ltd, Shanghai, 201203 China; 10 Department of Oncology, Tongji University Shanghai East Hospital, No. 150 Jimo Road, Pudong District Shanghai, 200120 China

**Keywords:** Fruquintinib, Metastatic colorectal cancer, Progression-free survival, VEGFR

## Abstract

**Background:**

To assess the efficacy and safety of fruquintinib, a vascular endothelial growth factor receptor (VEGFR) inhibitor, in metastatic colorectal cancer (mCRC) patients.

**Methods:**

A phase Ib open-label study and phase II randomized, placebo-controlled trial compared the efficacy of fruquintinib plus best supportive care (BSC) with placebo plus BSC in mCRC patients with ≥2 lines of prior therapies. The primary endpoint was progression-free survival (PFS).

**Results:**

In the phase Ib study, 42 patients took fruquintinib 5 mg for 3 weeks on/1 week off. The median PFS was 5.80 months, and the median overall survival (OS) was 8.88 months. In the phase II study, 71 patients were randomized (47 to fruquintinib, 24 to placebo). PFS was significantly improved with fruquintinib plus BSC (4.73 months; 95% confidence interval [CI] 2.86–5.59) versus placebo plus BSC (0.99 months; 95% CI 0.95–1.58); (hazard ratio [HR] 0.30; 95% CI 0.15–0.59; *P* < 0.001). The median OS was 7.72 versus 5.52 months (HR 0.71; 95% CI 0.38–1.34). The most common grade 3–4 adverse events were hypertension and hand-foot skin reaction.

**Conclusions:**

Fruquintinib showed a significant PFS benefit of 3.7 months in patients with treatment-refractory mCRC. The safety profile was consistent with that of VEGFR tyrosine kinase inhibitors. A randomized phase III confirmatory study in mCRC is underway.

**Trial registration:**

NCT01975077 and NCT02196688

**Electronic supplementary material:**

The online version of this article (doi:10.1186/s13045-016-0384-9) contains supplementary material, which is available to authorized users.

## Background

In patients with metastatic colorectal cancer (mCRC), addition of anti-vascular endothelial growth factor (VEGF) and anti-endothelial growth factor receptor (EGFR) biologic agents to chemotherapy regimens, either in the first or second line, improves overall survival (OS), progression-free survival (PFS), and anti-tumor response compared with chemotherapy alone [1–3]. However, patients frequently develop resistance and ultimately experience disease progression, highlighting a demand for more therapeutic strategies after failure of standard chemotherapy [4, 5].

Angiogenesis is an important hallmark of cancer development and progression. The VEGF and vascular epidermal growth factor receptor (VEGFR) signaling pathways strongly promote tumor growth and metastasis. Inhibition of these pathways has demonstrated strong clinical anti-tumor activity against multiple types of cancer, leading to the successful approval of both monoclonal antibody drugs and small molecule VEGFR inhibitors [6–8]. For instance, bevacizumab, a VEGF-directed monoclonal antibody, has been approved for the treatment of mCRC [9]. Recently, regorafenib has been approved by the US Food and Drug Administration based on the results from the CORRECT study [10].

Fruquintinib is a potent and highly selective small molecule inhibitor of VEGFR-1, VEGFR-2, and VEGFR-3 tyrosine and has shown strong anti-tumor activity in various preclinical models [11, 12]. In phase I trials, fruquintinib demonstrated good pharmacokinetic properties, tolerable safety, and promising anti-tumor activity against multiple tumor types [13]. A further two-regimen comparison study was carried out [14], and a regimen of 5 mg once daily oral dose on a 3-week-on/1-week-off treatment cycle was determined as the recommended phase II dose (RP2D). We report here data on a phase Ib expansion trial (NCT01975077) and a randomized, double-blind, placebo-controlled, multicenter phase II trial (NCT02196688) to further assess the safety and efficacy of fruquintinib at the RP2D in patients with mCRC who failed at least two prior standard treatments.

## Methods

### Study design and participants

We conducted an open-label phase Ib trial in two hospitals (NCT01975077) and a randomized, double-blind, placebo-controlled phase II trial in eight hospitals (NCT02196688) in China. Most of the inclusion and exclusion criteria were common to both the studies. Patients were eligible to participate when they had histological or cytological documentation of adenocarcinoma of the colon or rectum. Patients had to have received at least a second-line standard therapy, including fluoropyrimidine, oxaliplatin, or irinotecan-based regimens and to have disease progression within 3 months after the last administration of the last standard therapy or to have stopped such therapy due to unacceptable toxicities. Pre-treatment with EGFR and VEGF inhibitors (bevacizumab and aflibercept) were allowed but were not mandatory.

Patients had to be aged between 18 and 75 years and have an Eastern Cooperative Oncology Group performance status of 0 or 1, life expectancy of at least 12 weeks, and adequate bone marrow, liver, and renal function at the start of the trial. Patients could not participate if they had previously received any VEGFR inhibitors (regorafenib, ramucirumab, apatinib, axitinib, famitinib, or other tyrosine kinase inhibitors) or had other uncontrolled medical disorders. Additional files 1 and 2 show full inclusion and exclusion criteria for both of the studies.

These studies were conducted in accordance with the laws and regulations in China regarding patient protection. The studies were approved by the independent ethics committees of each involved institution. Informed consent was obtained from all the participants.

### Randomization and treatment

Patients who met the eligibility criteria for the phase Ib study took fruquintinib 5 mg once daily, for 3 weeks on and 1 week off. In the phase II trial, the eligible participants were randomly assigned in a 2:1 ratio to receive fruquintinib plus BSC or placebo plus BSC. The participants, investigators, and the study funder were masked to treatment group assignment. Randomization was performed centrally using the interactive web response system (IWRS), and no stratified randomization was performed. Unblinding could occur for individual patients via the IWRS in the case of emergencies only, and serious adverse events (AEs) did not necessarily precipitate immediate unblinding. All eligible participants repeated the 28-day treatment cycle until disease progression, death, unacceptable toxicity, withdrawal of consent by the patient, or decision by the treating physician that discontinuation would be in the patient’s best interest. The primary study endpoint was PFS.

Toxicity was graded using the National Cancer Institute-Common Terminology Criteria for Adverse Events version 4.03. Tumor assessment was performed every 8 weeks in the phase Ib trial and every 4 weeks during the first 4 cycles and every 8 weeks thereafter in the phase II trial until disease progression, which was based on computed tomography and/or magnetic resonance imaging evaluation as defined by the Response Evaluation Criteria In Solid Tumors version 1.1.

We allowed predefined treatment modifications to manage clinically significant toxicity. Patients who needed dose reductions could not re-escalate. The detailed treatment protocols are provided in Additional files 1 and 2.

### Statistical analysis

No formal statistical hypothesis testing was planned for the phase 1b study, and the planned primary population for the evaluation of efficacy was the intent-to-treat (ITT) population.

Based on the anti-tumor efficacy observed for fruquintinib in the phase Ib trial, the placebo-controlled phase II study was designed to have 67% statistical power to detect a 50.0% increase in the median PFS, assuming a 2-month median PFS for the placebo group. Assuming a two-sided overall *α* of 0.05, statistical power of 67%, randomization ratio of 2:1 between fruquintinib and placebo, and no interim analyses during the study, 6 months had to elapse after the last patient enrolled for the primary endpoint analysis of PFS and until mature OS data could be obtained for 80% of the patients for the final analysis. We planned to randomize approximately 70 patients.

All statistical analyses were performed using SAS (version 9.2). PFS and OS were compared between the treatment groups using a stratified log-rank test; HRs (with 95% confidence interval [CI]) were calculated using the Cox proportional hazards model, adjusting for stratification factors, and Kaplan–Meier survival estimates were calculated for each treatment group. The stratified factors included previous chemotherapy lines (2 versus ≥3), previous treatment with VEGF-targeting drugs (yes versus no), and liver metastases (yes versus no).

## Results

### Phase Ib trial

The demographic and baseline characteristics for the 42 participants with mCRC who were enrolled into the phase Ib study between December 26, 2012, and January 24, 2014, are shown in Table 1.

**Table 1:** Baseline characteristics of participants in the phase Ib and the phase II trials

Characteristics	Phase Ib *N* (%)	Phase II *N* (%)		
	(*n*=42)	Fruquintinib group (*n*=47)	Placebo group (*n*=24)	*P* value
Age (years)				
Median, range	55.5, 33.0–70.0	50.0, 25.0–69.0	54.0, 38.0–70.0	0.090
Gender				
Male	25 (59.5)	35 (74.5)	17 (70.8)	0.743
Female	17 (40.5)	12 (25.5)	7 (29.2)	
Baseline ECOG PS score				
0	8 (19.0)	6 (12.8)	5 (20.8)	0.374
1	34 (81.0)	41 (87.2)	19 (79.2)	
Duration from first metastasis Diagnosis to randomization				
≤18 months	NA	20 (42.6)	14 (58.3)	0.208
>18 months	NA	27 (57.4)	10 (41.7)	
Prior treatment line on or above metastatic disease				
2–3	18 (42.9)	30 (63.8)	17 (70.8)	0.555
>3	24 (57.1)	17 (36.2)	7 (29.2)	
Previous chemotherapy lines				
2	5 (11.9)	12 (25.5)	7 (29.2)	0.743
≥3	37 (88.1)	35 (74.5)	17 (70.8)	
Prior VEGF inhibitor treatment				
Yes	10 (23.8)	15 (31.9)	7 (29.2)	0.412
No	32 (76.2)	29 (61.7)	17 (70.8)	
Unknown	0	3 (6.4)	0	
Primary site				
Colon	21 (50.0)	24 (51.1)	13 (54.2)	0.804
Rectal	20 (47.6)	23 (48.9)	11 (45.8)	
Cecum	1 (2.4)	0	0	
Metastatic site				
Single	5 (11.9)	2 (4.3)	2 (8.3)	0.481
Multiple	37 (88.1)	45 (95.7)	22 (91.7)	
Liver metastasis				
Yes	29 (69.0)	29 (61.7)	17 (70.8)	0.446
No	13 (31.0)	18 (38.3)	7 (29.2)	

*ECOG PS* Eastern Cooperative Oncology Group performance status, *VEGF* vascular endothelial growth factor, *NA* not available

Thirty-one (73.8%) participants completed at least three treatment cycles in 12 weeks, and 28 (66.7%) participants completed at least four treatment cycles in 16 weeks. Dose reduction and interruption was necessary in 20 participants (47.6%).

The median PFS was 5.80 months (95% CI 4.01–7.60), and the median OS was 8.88 months (95% CI 7.53–15.53). Four participants had partial response (PR) with an objective response rate of 9.5%, and 28 participants had stable disease for at least 8 weeks, with a disease control rate (DCR) of 76.2%. The treatment efficacy is summarized in Table 2.

**Table 2:** Treatment efficacy in the phase Ib and the phase II trials

	Phase Ib	Phase II		
	(*N*=42)	Fruquintinib group (*N*=47)	Placebo group (*N*=24)	*P* value
Median PFS months, 95% CI	5.80, 4.01 to 7.60	4.73, 2.86 to 5.59	0.99, 0.95 to 1.58	<0.001
Median OS months, 95% CI	8.88, 7.53 to 15.53	7.72, 6.90 to 10.28	5.52, 3.61 to 11.30	0.29
CR No. (%)	0	0	0	
PR No. (%)	4 (9.5)	1 (2.1)	0	
SD No. (%)	28 (66.7)	31 (66.0)	5 (20.8)	
PD No. (%)	7 (16.7)	12 (25.5)	17 (70.8)	
Not evaluable, No. (%)	3 (7.1)	2 (4.3)	1 (4.2)	
No post-baseline assessment	0	1 (2.1)	1 (4.2)	
ORR No. (%), 95% CI	4 (9.5)	1 (2.1), 0.1 to 10.7	0, 0.0 to 12.6	0.45
DCR No. (%), 95% CI	32 (76.2)	32 (68.1), 53.6 to 80.8	5 (20.8), 8.6 to 40.6	<0.001

*P* values are the results of stratified analyses for comparisons between the fruquintinib group and placebo group in the phase II trial. ORR = CR + PR, DCR = CR + PR + SD

*CI* confidence interval, *No.* number of participants, *PFS* progression-free survival, *OS* overall survival, *CR* complete response, *PR* partial response, *SD* stable disease, *PD* progressive disease, *ORR* objective response rate, *DCR* disease control rate

Treatment-related treatment-emergent AEs (TEAEs) were reported in all 42 participants. The most common TEAEs of grade 3 or higher were hypertension (21.4%), hand-foot skin reaction (HFSR, 9.5%), and diarrhea (9.5%). Overall, fruquintinib was permanently discontinued in five participants (11.9%) due to related TEAEs, including skin lesion (*n* = 1), chest pain (*n* = 1), hemoptysis (*n* = 1), pancreatitis (*n* = 1), and proteinuria (*n* = 1). The incidences of grade 3 or higher TEAEs related to the study drugs are summarized in Table 3. Only one death, of a patient with lung metastasis who had fatal hemoptysis, was considered to be possibly treatment related by the investigator. The most common TEAEs needing treatment modification (treatment interruption or dose reduction) were thrombocytopenia (11.9%), HFSR (11.9%), and hypertension (9.5%).

**Table 3:** Grade 3 or above treatment-related TEAEs occurring in at least 4% of patients in the phase Ib and the phase II trials

Preferred term	Phase Ib *N* (%)	Phase II *N* (%)	
	(*n*=42)	Fruquintinib group (*n*=47)	Placebo group (*n*=24)
Hypertension	9 (21.4)	14 (29.8)	0
HFSR	4 (9.5)	7 (14.9)	0
Diarrhea	4 (9.5)	1 (2.1)	0
Serum sodium decreased	3 (7.1)	0	0
Fatigue	2 (4.8)	2 (4.3)	0
Chest pain	2 (4.8)	0	0
Blood bilirubin increased	1 (2.4)	2 (4.2)	1 (4.2)
AST increased	0	1 (2.1)	1 (4.2)
Platelet count decreased	0	1 (2.1)	1 (4.2)
Blood alkaline phosphatase increased	0	0	2 (8.3)
Myalgia	0	0	1 (4.2)
Coma hepatic	0	0	1 (4.2)
Infection	0	0	1 (4.2)

*TEAE* treatment-emergent adverse event, *HFSR* hand-foot skin reaction, *AST* aspartate aminotransferase

### Phase II trial

**Fig. 1:**
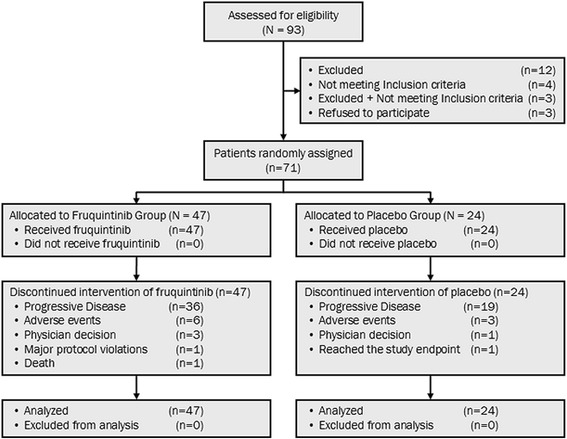
Trial profile

Between April 1, 2014, and August 20, 2014, 93 patients were screened and 71 patients were randomized to receive fruquintinib (*n* = 47) or placebo (*n* = 24). All 71 participants underwent treatment for efficacy and safety analyses (Fig. 1).

The baseline characteristics for all randomized patients are shown in Table 1. In general, the two groups were well balanced in terms of baseline demographics and oncology disease history.

Participants in the fruquintinib group were treated for a longer period than were those in the placebo group, with mean treatment durations (from the first dose to the end of treatment) of 3.2 versus 0.8 months, respectively. Dose modifications were required in 29 (61.7%) of 47 participants who received fruquintinib and 7 (29.2%) of 24 participants who received placebo. AEs were the most frequent reasons for dose modification.

**Fig. 2:**
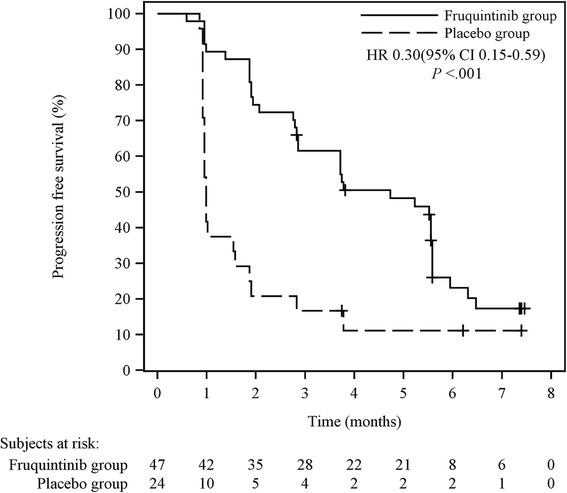
Kaplan–Meier curves of progression-free survival (PFS) in the phase II study

**Fig. 3:**
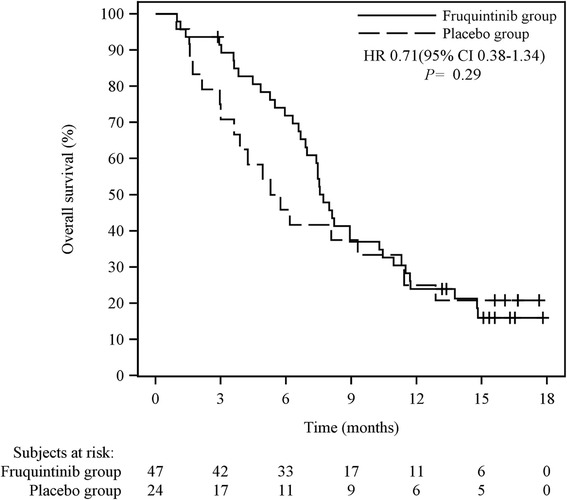
Kaplan–Meier curves of overall survival (OS) in the phase II study

PFS was significantly prolonged for patients who were treated with fruquintinib compared with patients who received placebo (stratified HR 0.30; 95% CI 0.15–0.59; two-sided *P* < 0.001; Fig. 2), which was consistent with the results of a blinded independent central review (stratified HR 0.26; 95% CI 0.14–0.50; two-sided *P* < 0.001). The median PFS was 4.73 months (95% CI 2.86–5.59) in the fruquintinib group and 0.99 months (95% CI 0.95–1.58) in the placebo group. Pre-specified subgroup analyses showed significantly superior PFS in the fruquintinib group in most of the subgroups examined (Additional file 3). Patients who received fruquintinib showed a trend of prolonged median OS (7.72 months) compared with those who received placebo (5.52 months); however, the difference was not significant (stratified HR 0.71; 95% CI 0.38–1.34; Fig. 3). Only one patient (2.1%) in the fruquintinib group achieved PR. The DCR was significantly higher in the fruquintinib group than in the placebo group (68.1% versus 20.8%; two-sided *P* < 0.001). The waterfall plots for tumor responses are shown in Additional file 4. The summary of drug efficacy is shown in Table 2.

All 47 (100%) participants in the fruquintinib group and 21 (87.5%) of the 24 participants in the placebo group had AEs; the AEs were deemed treatment-related in 44 (93.6%) participants in the fruquintinib group and 14 (58.3%) participants in the placebo group. The most common (incidence >5%) treatment-related grade 3 or higher TEAEs were hypertension (29.8%) and HFSR (14.9%). The summary of grade 3 or higher treatment-related TEAEs is shown in Table 3. Frequencies of AEs leading to death, irrespective of relationship to study drug, were similar, at three in the fruquintinib group (one [2.1%] upper gastrointestinal hemorrhage, one [2.1%] bilirubin increased, and one [2.1%] hemoptysis) and two in the placebo group (one [4.3%] sudden death and one [4.3%] hepatic coma). Serious AEs occurred in 12 (25.5%) of the 47 participants receiving fruquintinib and 5 (20.8%) of the 24 participants receiving placebo. Treatment was interrupted because of AEs in 15 (34.0%) patients in the fruquintinib group and in 4 (16.7%) in the placebo group. The study dose was reduced due to AEs in 13 (27.7%) participants in the fruquintinib group and none in the placebo group. The common AEs that required treatment modification were HFSR (17.0%), hypertension (12.8%), and diarrhea (4.3%).

## Discussion

In the phase II trial, fruquintinib significantly prolonged PFS to approximately 4.7 versus 1.0 month for placebo. This supports the significantly higher DCR in the fruquintinib group. Moreover, a blinded independent central review of this randomized phase II study confirmed the PFS benefit conferred by fruquintinib (3.71 versus 0.95 months, Additional file 5). A similar anti-tumor efficacy with a 5.8-month PFS was observed for patients who were enrolled in the phase Ib trial. These two studies show the potential of fruquintinib as a third-line treatment for mCRC.

In accordance with the results obtained from other studies investigating anti-VEGF/VEGFR drugs, our study verified that this pathway is an effective target for the treatment of mCRC. Similar to the results obtained from the administration of regorafenib and aflibercept as monotherapies in previously treated mCRC patients, very few patients reached PR and a general benefit was mainly observed in patients with stable disease [10, 15, 16]. However, more than half of the patients who received fruquintinib showed tumor shrinkage to different degrees, as demonstrated in the waterfall plots (Additional file 4), suggesting a substantial anti-tumor effect for fruquintinib.

Compared with BSC, OS was prolonged for about 2.2 months in patients who received fruquintinib, although no statistically significant difference was observed. The improvement of OS by 2.2 months was similar to the results of the regorafenib CONCUR trial [15] that was predominantly conducted in the Chinese population. Considering the small sample size in the present phase II trial, it was speculated that a more defined OS benefit might be observed if the sample size was expanded in future clinical studies. Thus, a phase III clinical trial (NCT02314819) with OS as the primary endpoint is currently underway in patients with mCRC who have failed standard treatment.

The safety profile in the phase II trial was consistent with that of the patients in the phase Ib trial, and the AEs included HFSR, hypertension, and proteinuria. These results were consistent with the results of studies conducted using other selective VEGFR inhibitors such as regorafenib [10]. In general, treatment with fruquintinib was well tolerated. As has been reported elsewhere for other multi-kinase inhibitors, we observed that the occurrence of grade 3 hypertension was more frequent in our trial (21.4% of the 42 participants in the phase Ib trial and 29.8% of the 47 participants in the phase II trial). Nonetheless, neither grade 4 hypertension nor hypertensive crisis were reported and no patients discontinued treatment. Hypertension is commonly observed with anti-angiogenic agents, and it could be managed using standard anti-hypertensive agents if required. Moreover, some retrospective analyses have suggested that the development of high blood pressure might be a predictor of good clinical outcome [17, 18]. The incidence of HFSR (64%) in the present study was slightly higher than that reported by the CORRECT trial [11]; however, it was consistent with that of the CONCUR study [15]. Approximately 15% of the patients were reported to have developed grade 3 HFSR; however, the symptoms were clinically manageable by dose interruption or reduction. Frequencies of treatment modification (treatment interruption or dose reduction) were similar between the phase II study (34% and 27%, respectively) and phase 1b study (47.6%) but were slightly lower than in CONCUR (63% and 40%, respectively) [15] or CORRECT (61% and 38%, respectively) [10]. Unexpected safety issues did not arise, and no patient had bowel perforation, which has been related to other VEGF agents.

One limitation of this study was that a predictive biomarker for fruquintinib was not investigated. However, considering the lack of a definitive predictive biomarker for bevacizumab, another anti-VEGF drug that has been widely used in clinical practice, it is expected that identifying a predictive biomarker for fruquintinib would require some time [18]. In addition, we did not collect any information on the RAS and BRAF expression status of the tumors and therefore could not investigate whether any relationship existed between the RAS status of the tumor and the efficacy of fruquintinib. However, it should be noted that previous studies suggested that the RAS and BRAF status had no predictive value for outcome in mCRC patients treated with bevacizumab [19].

## Conclusions

In conclusion, based on the present trials, fruquintinib showed good performance in both safety and efficacy and might be a suitable treatment for mCRC resistant to standard treatment. The phase III trial (NCT02314819) that is currently ongoing will help to achieve a definitive assessment of the safety and efficacy of fruquintinib in mCRC patients who failed the second-line or above treatment.

## Additional files

Additional file 1: Protocol for phase Ib study (NCT 01975077). (PDF 636 kb)
Additional file 2: Protocol for phase II study (NCT 02196688). (PDF 1031 kb)
Additional file 3: Forest plot of subgroup analysis in phase II study. (PDF 104 kb)
Additional file 4: The waterfall plots for tumor responses in phase II study. (PDF 49 kb)
Additional file 5: Analysis of progression-free survival in phase II study by blinded independent central review. (PDF 129 kb)
