# Supplementary material for: Safety and efficacy of fruquintinib in patients with previously treated metastatic colorectal cancer: a phase Ib study and a randomized double-blind phase II study
Source: J Hematol Oncol. 2017 Jan 19;10:22. doi: 10.1186/s13045-016-0384-9 (PMC5244709; doi:10.1186/s13045-016-0384-9)
Supplement: Additional file 1: — Protocol for phase Ib study (NCT 01975077). (PDF 636 kb) [file 13045_2016_384_MOESM1_ESM.pdf]

Front Page of Clincal Study Protocol of Fruquintinib

Protocol No.: **2012-013-00CH3**

Date of establishment**: 2014-08-14 Version Amendment IV**

Study Protocol

Phase Ib Clinical Study of Fruquintinib (3rd-line or above) for the Treatment of Advanced Colorectal Cancer

Title: a randomized, open-label, phase Ib clinical study comparing Fruquintinib 4mg qd continuous with 5 mg qd 3-week on/1-week off as 3rd-line or above treatment in patients with advanced colorectal cancer who have failed with standard therapy

Clinical study protocol No.:	2012-013-00CH3
Study drug:	Fruquintinib (HMPL-013)
Study unit of team leader:	Fudan University Shanghai Cancer Center
Principal investigator:	Li Jin
Co-principal investigator:	Cao Junning
Unit participating in the study:	Sun Yat-Sen University Cancer Center
Primary responsible person：	Xu Ruihua
Sponsor:	Hutchison Medi Pharma (Shanghai) Co., Ltd.
Unit for statistics:	Beijing Bontz Technology Limited Co.

Proprietary right statement: this protocol is the property of Hutchison Medi Pharma (Shanghai) Co., Ltd., and must not be disseminated, copied or published by any one or business unit without authorization.

Signature page of clinical study protocol

Protocol title: A randomized, open-label, phase Ib clinical study comparing Fruquintinib 4mg qd continuous with 5 mg qd 3-week on/1-week off as 3rd-line or above treatment in patients with advanced colorectal cancer who have failed with standard therapy

Clinical study protocol number: 2012-013-00CH3, amendment IV，date of establishment**:** August 14, 2014

Here, I confirm that I have read, understood and agreed to follow all the items in the clinical study protocol number 2012-013-00CH3 dated _____/_____/_____, and will execute my responsibilities conscientiously in accordance with the provisions in Good Clinical Practice.

Sponsor’s signature	Date
Principal investigator (signature)	Date

(Emergency) contact of Hutchison Medi Pharma (Shanghai) Co., Ltd.

In the case of a medical emergency, the study director of study team may be contacted.When the study director can not be reached, you can contact the safety reporting contact person listed in the table below.

	Name	Telephone number
Study Director	FAN Songhua	021-20673058<br>18516082311
Safety reporting contact person	XI Xiaoyun	021-20673061<br>18601688469

Synopsis of phase Ib clinical study protocol

Name of Study Drug	Fruquintinib (laboratory code: HMPL-013)
Protocol number	**2012-013-00CH3**
Study title	A randomized, open-label, phase Ib clinical study comparing Fruquintinib 4mg qd continuous with 5 mg qd 3-week on/1-week off as 3rd-line or above treatment in patients with advanced colorectal cancer who have failed with standard therapy
Sponsor	Hutchison Medi Pharma (Shanghai) Co., Ltd.
Principle of drug action	Fruquintinib is a potent, anti-angiogenic small molecular compound with vascular endothelial growth factor (VEGF) receptor kinase family (VEGFR1, 2 and 3) as the target.Fruquintinib has a very high selectivity for kinase, with a good pharmacokinetic profile and safety index in animal models, and showed a powerful tumor inhibition in a variety of human tumor models in nude mice. At the same dose, its antitumor activity is more potent than sunitinib.<br>Phase I clinical trial on tolerability showed Fruquintinib had a significant antitumor activity for advanced intestinal cancer, gastric cancer, lung cancer and breast cancer.
Study Sites	Two study sites: Fudan University Shanghai Cancer Center, Sun Yat-sen University Cancer Center
Planned number of patients	About 60 subjects are planned to be enrolled.<br>40 patients are enrolled in the randomized comparison part, and about 20 patients are added for the single-arm expansion part.<br>Randomized comparison part:<br>Group A (4mg daily)-20 patients; group B (5 mg 3-week on/1-week off)-20 patients<br>Expansion part:<br>5mg qd 3-week on/1-week off – about 20 patients
Target Population	Histologically or cytologically confirmed advanced colorectal cancer after failure of second-line and above chemotherapy or standard care.<br>Note: treatment failure is defined as: progression of disease<br>a. each-line of therapy for progressive disease includes one or multiple drugs used for ≥1 cycle or longer time<br>b. Early adjuvant/neoadjuvant therapy is allowed.If recurrence or metastasis occurs during or within 6 months after the completion of adjuvant/neoadjuvant therapy, the adjuvant/neoadjuvant therapy is considered as one first-line early systemic chemotherapy for one progressive disease.<br>c. The early therapy allowed is defined as chemotherapy combined with targeted drugs except VEGFR
Study start and end time	The recruitment period for the randomized comparison part is estimated to be 8 months.The recruitment period for the expansion part is estimated to be 4 months.<br>Estimated start time: December 2012<br>Enrollment completion time for the expansion part: January 24, 2014<br>End time: October 24, 2014
Study objectives	**Primary objective:**<br>To randomly compare the safety and tolerability of two different administration methods of Fruquintinib (4mg qd daily vs. 5mg qd 3-week on/1-week off) in treatment of patients with advanced colorectal cancer who have failed with standard care (evaluated according to CTCAE4.0), as to further determine the recommended dose for phase II/III clinical studies.<br>To further observe and evaluate the safety, toleratbility and efficacy (progression-free survival) of Fruquintinib 5mg qd 3-week on/1-week off in treatment of patients with advanced colorectal cancer who have failed with standard care during the expansion

	part.
	**Secondary objective:**<br>● To compare the objective response rate (ORR), disease control rate (DCR), duration of response (according to RECIST 1.1 criteria), progression-free survival (PFS) and overall survival (OS) between two groups<br>● To compare the pharmacokinetic profile of two different administration methods of Fruquintinib monotherapy in treatment of advanced colorectal cancer<br>● Expansion part- to further observe the DCR, ORR and OS of Fruquintinib in treatment of advanced colorectal cancer
Trial Design	This is a randomized, multi-center, phase Ib clinical study comparing the safety and efficacy of two administration methods of Fruquintinib in treatment of patients with advanced colorectal cancer who have failed with standard care, as to further determine the administration method and recommended dose for phase II/III studies.<br>The study will be conducted in two parts:<br>1、 Randomized comparison part: to compare the safety and efficacy of two administration methods of Fruquintinib in treatment of patients with advanced colorectal cancer who have failed with standard care, as to further determine the recommended dose for phase II/III studies.<br>2、 Expansion part: to perform the expansion part with the determined recommended dose for phase II/III studies. The regimen of 5mg qd 3-week on/1-week off is selected for expansion part according to the results from randomized comparison part.<br>All the patients will receive Fruquintinib monotherapy in a cycle of 4 weeks, and receive tumor assessment every 8 weeks until progression of disease, intolerable toxicity or withdrawal of informed consent.
Dosage and route of administration	Randomized comparison part:<br>Group A: Fruquintinib 4mg qd po, daily<br>Group B: Fruquintinib 5mg qd po, 3-week on/1-week off<br>Expansion part: Fruquintinib 5mg qd po, 3-week on/1-week off
assessment	
Safety	The adverse event is adjudicated and graded in accordance with NCI-CTCAE V4.0. The comprehensive evaluation of safety and tolerability is performed according to the severity and incidence of adverse event.The primary safety endpoints includes the overall incidence of adverse events, incidence of Grade 3/4 adverse events and serious adverse events.<br>The tolerability will be comprehensively evaluated based on the occurrence of discontinuation of the drug or dose reduction due to adverse event.
Effectiveness	Randomized comparison part:<br>The secondary endpoints are objective response rate, disease control rate, duration of response, progression-free survival and overall survival determined in accordance with RECIST1.1<br>Expansion part:<br>The primary efficacy endpoint is progression-free survival at Week 16<br>The secondary efficacy endpoints are DCR, ORR and OS.
PK study	To explore the pharmacokinetic (PK) profile of two different administration methods of Fruquintinib (4mg qd daily vs. 5mg qd 3-week on/1-week off), and to explore the relationship between PK and preliminary pharmacodynamic parameters as well as the main safety parameters
Inclusion Criteria	All the following conditions must be met:<br>1. Fully understanding of this study and voluntary signature of the informed consent form;<br>2. Histologically and/or cytologically confirmed advanced colorectal cancer after

	2nd-line or above treatment failure;<br>3. No systemic antitumor treatment or radiotherapy, immunotherapy, biogical or hormone therapy in the past 4 weeks; no treatment with VEGFR inhibitor;<br>4. Age = 18-70 years<br>5. Body weight >40KG;<br>6. Eastern cooperative oncology group (ECOG) socre 0 or 1, and no exacerbation within 7 days;<br>7. Evaluation of cardiac function: left ventricular ejection fraction ≥50% (echocardiography);<br>8. Definitely measurable lesion according to RECIST1.1;<br>9. Expected survival > 12 weeks.
Exclusion Criteria	Patients meeting any of the following conditions must be excluded from this study:<br>1. Absolute neutrophil count (ANC) $<1.5\times10^9$/L, platelet count $<80\ \times10^9$/L or hemoglobin < 9g/dL in the laboratory test performed wthin one week prior to enrollment;<br>2. Serum total bilirubin, glutamic-pyruvic transaminase, glutamic-oxaloacetic transaminase and alkaline phosphatase exceeding normal range (based on the normal value at clinical study site); alkaline phosphatase more than 2.5×ULN for patients with liver metastasis;<br>3. Serum creatinine exceeding the upper limit of normal range (based on the normal value at clinical study site) or creatinine clearance lower than 50ml/min;<br>4. Systolic blood pressure ≥150mmHg and/or diastolic blood pressure ≥100mmHg regardless of the use of antihypertensive agent;<br>5. Serum potassium exceeding normal range with clinical significance, regardless of the supplementation of potassium preparation;<br>6. Serum calcium or magnesium exceeding normal range with clinical significance, regardless of any supplementation;<br>7. Urine protein 2+ or above, or 24-hour amount of urine protein ≥1.0g/24h;<br>8. Evidence of intestinal cavity infiltration of the tumor (with possibility of potential bleeding);<br>9. Unrecovered toxicity from previous anticancer therapy (CTCAE ＞ 1), not completely recovered from previous surgery or less than 4 weeks from the last anticancer therapy or surgery;<br>10. Presence of metastasis to central nervous system (CNS);<br>11. Clinically uncontrolled active infection, such as acute pneumonia, active phase of hepatitis B;<br>12. Dysphagia or known malabsorption to drugs;<br>13. Current digestive diseases such as duodenal ulcer, ulcerative colitis, or other condtions to possibly cause digestive tract bleeding or perforation as judged by investigators;<br>14. Bleeding tendency or history of bleeding within 2 months prior to enrollment, regardless of the severity;<br>15. Development of stroke within 12 months prior to enrollment, including transient ischemic attack;<br>16. Activated partial thromboplastin time (APTT) or/and INR and prothrombin time (PT) exceeding normal range with clinical significance (based on the normal value at clinical study site);<br>17. Incomplete healing of skin wound, severe mucosal ulcer or bone fracture;<br>18. Acute myocardial infarction, severe/unstable angina pectoris or coronary bypass surgery within 6 months prior to enrollment；history of arterial thrombosis or serious deep venous thrombosis<br>19. Pregnant or lactating women or positive pregnancy test in women of childbearing potential prior tot the first dose; premenopausal female subjects who are unwilling to use appropriate contraceptive measures (over one year from the last

	menstruation)<br>20. Unsuitability to partipating in the clinical study due to any clinical or laboratory abnormality as considered by investigators;<br>21. Severe metal or psychological illness, and inadequate compliance with the clinical study could be anticipated;<br>22. Participation in clinical trial of other drugs within the last 4 weeks.
Subjects Terminating the Study	Criteria on completion of the trial:<br>1. Progression of disease<br>2. Death<br>3. Inability to recover to ≤NCI CTC AE Grade 1 or baseline within 14 days after dose interruption<br>4. NCI CTC AE Grade 4 (nephrotic syndrome) in routine urinalysis or 24-hour urine protein quantification; CTC AE Grade 4 abnormal hepatic function and hemorrhage<br>Criteria on premature discontinuation：<br>1. The subject becomes pregnant.<br>2. The investigator judges the termination of the study in the subject's best interest;<br>3. The subject or his/her legal representative requestes withdrawal from the study;<br>4. The subject is lost to follow-up<br>5. Serious violation from the study protocol
End of the whole trial	The whole trial ends 9 months after the end of enrollment for the expansion part.

Statistical analysis:

SAS 9.2 software is used for analysis, where the pharmacokinetic parameters are calculated using WinNonlin software.

The detailed statistical method will be provided in the statistical analysis plan.

In the randomized comparison part, statistical analysis and comprehensive evaluation will be performed for the primary parameters including safety and tolerability (overall incidence of adverse events, incidence of G3/4 adverse events, incidence of serious adverse events, incidence of adverse events leading to dose interruption and permenant discontinuation of the drug) and secondary parameters including the efficacy, as to determine the recommended dose for phase II/III trials.

In the expansion part, the safety, tolerability and efficacy of Fruquintinib 5mg 3-week on/1-week off are further evaluated in patients with advanced colorectal cancer.The primary endpoints (safety, tolerability and progression-free survival at Week 16) are analyzed three months after the inclusion of the last subject in the expansion part.The overall survival is analyzed 9 months after the inclusion of the last subject in the expansion part.

Efficacy	To perform statistical description of objective response rate (proportion of subjects with complete response or partial response as the optimal overall response, according to RECIST1.1 criteria), DCR, DOR, PFS and OS.
Safety	All the adverse events will be listed by subject, and MedDRA is used to code adverse events by organ system and standard terminology for statistical description.Abnormal values in laboratory examinations, vital signs, ECG and other safety parameters are described by subject list.<br>The overall incidence of adverse reactions, incidence of G3/4 adverse events and incidence of serious adverse events are compared and evaluated for the safety between the two groups.<br>Proportions of dose interruption /dose reduction as well as permenant discontinuation due to study related adverse reaction are compared and evaluated for the tolerability between the two groups.<br>Expansion part- the safety and tolerability of 5mg qd 3-week on/1-week off are further observed in treatment of advanced colorectal cancer.The overall incidence of adverse reactions, incidence of G3/4 adverse events and incidence of serious adverse events

	are evaluated.
Pharmacokinetic Studies	Non-compartment model analysis of data on the plasma concentration is performed by the central laboratory using WinNonlin software (enterprise edition).<br>One-way analysis of variance will be used for the Cmax, $AUC_{0-\infty}$, and AUC_{0-t} (to be determined according to the opinions from the department of pharmacokinetics) during the trial, using dose group as the fixed factor.

(Emergency) contact of Hutchison Medi Pharma (Shanghai) Co., Ltd. 3
Synopsis of phase Ib clinical study protocol 4
Abbreviations and Special Term 11
1. Background 14
1.1 Colorectal cancer 14
1.2 Fruquintinib 15
1.2.1 Preclincial evidence of Fruquintinib 15
1.2.2 Results of phase I clinical trial of Fruquintinib (preliminary results as of October 30, 2012) 16
1.2.2.1 Maximum tolerated dose (MTD) of Fruquintinib in subjects with advanced malignant solid tumor 16
1.2.2.2 Safety and tolerability 17
1.2.2.3 Observation of clinical efficacy: 25
1.2.2.4 Recommended dose for phase Ib clinical trial 26
1.2.2.5 Pharmacodynamics results 26
1.2.3 Results from long-term toxicology study of Fruquintinib 26
1.2.3.1 Long-term toxicity study in SD rats given Fruquintinib orally for 26 weeks followed by 4-week recovery period 26
1.2.3.2 Long-term toxicity study in Beagle dogs given Fruquintinib (drug product) orally for 39 weeks followed by 4-week recovery period 27
2 Study objectives 27
2.1 Primary objectives 27
2.2 Secondary objectives 27
3 Trial Design 28
3.1 SUMMARY OF EXPERIMENTAL DESIGN 28
3.1.1 Rationale for Study Design 28
3.1.2 Rationale for Dose Selection. 28
3.2 Number of subjects 30
3.3 Study Sites 30
4 Study Population 30
4.1 Target Population 30
4.2 Enrollment criteria 30
4.3 Exclusion Criteria 31
4.4 Criteria for treatment termination 32
4.5 Exclusion Criteria 33
4.6 End of Trial 33
5 Treatment 33
5.1 Dosage and administration regimen of study drug 33
5.2 Source, strength and shelf-life of the product 34
5.3 Product Labeling 34
5.4 Drug storage 34
5.5 Grouping method and drug dispension 34
5.6 Dosage and administration method 35
5.7 Administration method 35
5.8 Accountability 35
5.9 Principle and method for management of toxic and side effects after administration 36
5.9.1 Principle on management of toxic and side effects during the study 36
5.9.2 Management of the expected adverse events of Fruquintinib 36
5.9.3 Provisions on dose adjustment resulted from toxicity： 39
5.9.4 Management of Overdose 40
5.10 Concomitant therapy 40
5.11 Other concomitant treatments 41
5.12 Treatment compliance 41
6 Variables, evaluation plan and steps 41
6.1 Screening examination and eligibility screening form 45
6.2 Clinical Assessment 45
6.2.1 Effectiveness 45
6.2.1.1 Effectiveness endpoint 45

6.2.1.2 Assessment of effectiveness 45
6.2.2 Performance status 46
6.2.3 Clinical safety evaluation 46
6.2.3.1 Safety endpoints 46
6.2.3.2 Evaluation of adverse events and safety laboratory parameters 46
6.2.4 Measurement of drug concentration and pharmacokinetic study 47
6.2.5 Subsequent antitumor therapy 50
7 Safety evaluation 50
7.1 Safety parameters and definition 50
7.1.1 Adverse event 50
7.1.2 Serious adverse event 50
7.1.3 Protocol-defined special events 51
7.1.4 Progression of disease 51
7.2 Collection and evaluation of safety parameters 52
7.2.1 Definition of adverse event reporting period 52
7.2.2 Intensity assessment of adverse events 52
7.2.3 Drug-event relationship 52
7.3 Recording and reporting of safety parameters 53
7.3.1 Recording of adverse events. 53
7.3.2 Expedited reporting of serious adverse events 54
7.3.3 Reporting of protocol-defined special events 54
7.3.4 Recording and reporting of abnormal laboratory examinations 55
7.4 Follow-up of subject 55
7.4.1 Follow-up of Adverse Event(s) 55
7.4.2 Follow-up of Serious Adverse Event(s) 56
7.4.3 Follow-up of protocol-defined special events 56
7.4.4 Follow-up of abnormal laboratory results 56
7.5 Pregnancy and management of pregnancy 56
8 Statistics and analysis plan 57
8.1 Primary and secondary research variables 57
8.2 Statistical and analytical methods 58
8.2.1 Data Set Analyzed 58
8.2.2 Statistical Methods 58
8.2.3 Statistical Software and General Requirements 59
8.2.4 Interim Analyses 59
8.3 Sample size 60
9 Data Quality Assurance 60
10 Study committee 61
11 Ethics 61
11.1 Local Regulations/Declaration of Helsinki 61
11.2 Informed Consent 61
11.3 Independent Ethics Committee/Institutional Review Board 62
12 Conditions to Change the Protocol 62
13 Condition to End the Protocol 63
14 Record, Storage, and Access to the Study Documents 63
14.1 Storage of Investigator's Documents/Records 63
14.2 Original Documents and Background Information 63
14.3 Direct Access to Original Data and Documents 63
15 Study monitoring 63
16 Confidentiality of the Trial Documents and Patients' Records 64
17 Publication of the Data, and Protection of Business Secrets 64
18 References 64
Appendix 1 ECOG performance status 66
Appendix 2 RECIST version 1.1 for Tumor Evaluation 67
Appendix 3 Sketch of Safety Data Reporting 83
Appendix 4 Clinical Evaluation of Hepatic Impairment 84
Appendix 5 Shipment and Processing of Pharmacokinetic Samples 86

Abbreviations and Special Term

Abbreviations and Special Term	Explanation
AE	Adverse event
A/G	Albumin/globulin
ALB	Albumin
ALP	Alkaline phosphatases
ALT	Alanine aminotransferase
ANC	Absolute neutrophils count
APTT	Partial thromboplastin time
AST	Aspartate transaminase
AUC_{ss}	Area under the plasma concentration-time curve over one dosing interval at steady state
Best Supportive Care BUN	Urea nitrogen
CEA	Carcino Embryonic Antigen
CHOL	CHOLESTEROL
c-MET	Mesenchymal-epithelial transition factor
CNS	Central Nervous System
CR	Complete response
CRF (eCRF)	Case report form(s) (electronic case report form)
CT	Computer tomography
CTC	Common toxicity criteria
CTCAE	Common Terminology Criteria For Adverse Events
DCR	Disease Control Rate
DFS	Disease free survival
DLT	Dose limiting toxicity
ECG	Electrocardiogram
ECOG	Eastern Co-operative Oncology Group
EGFR	Epidermal growth factor
EORTC	European Organisation for Research and Treatment of Cancer
Ethics Committee	Ethics Committee
FDA	Food and Drug Administration FGA
FDG-PET	Fluorodeoxyglucose - positron emission tomography
FIB	Fibrinogen.
GCP	Good Clinical Practice
Glu	Glucose Values
HFS	Hand-foot syndrome
HUVEC	Human umbilical vein endothelia cell
IARC	International Agency for Research on Cancer
IC50	Half inhibitory concentration
ICH	International Conference on Harmonisation

Abbreviations and Special Term	Explanation
IDMC	Independent Data monitoring committee
INR	International normalized ratio
ITT	Intent-to-treat
LDH	Lactic Dehydrogenase
LOAEL	Lowest observed adverse effect level
LVEF	Left ventricular ejection fraction
MedDRA	Medical Dictionary for Regulatory Activities
mg	Milligram(s)
ml	Millilitre(s)
MRI	Magnetic resonance imaging MRP
MTD	Maximum tolerated dose
NCCN	National Comprehensive Cancer Network
NCI	National Cancer Institute
NCI-CTC	National Cancer Institute Common Toxicity Criteria
NCI-CTCAE	National Cancer Institute Common Terminology Criteria for Adverse Events
NE	Non-Evaluable
NOAEL	No observed adverse effect level
NSCLC	Non-small-cell lung carcinoma
ORR	Objective response rate
OS	Overall survival
PD	Progressive disease or pharmacodynamics
PDGFR	Platelet derived growth factor receptor
PFS	Progression-free Survival
p.o.	per os
PR	Partial response
PS	Performance status
PSA	Prostate-specific antigen
PT	Prothrombin time
QD	Qd
RECIST	Response Evaluation Criteria in Solid Tumors
SAE	Serious adverse event
SD	Stable disease/standard devivation
TG	Triglyceride
TKi	Tyrosine kinase inhibitor
TP	Total protein
TT	Thrombin time
TTP	Time to Progression
TSH	thyroid stimulating hormone

Abbreviations and Special Term	Explanation
VEGF	Vascular endothelial growth factor
VEGFR	Vascular endothelial growth factor receptor
WHO	World Health Organization

1. Background

1.1 Colorectal cancer

Colorectal cancer is one of the most common malignancies, and ranks No.3 in the incidence among all the malignancies worldwide, regardless of male or female. In the western developed countries, colorectal cancer is the 2nd common malignancy in incidence, and took the No. 3 place in both the number of newly developing cases and mortality in US in 2003. As shown in the data provided by the International Agency for Research on Cancer (IARC), the incidence of colorectal cancer also increases rapidly in Asian, in particular in economically developed regions, and is approaching that in western countries. Colorectal cancer has been the second tumor in terms of incidence and mortality in our country since 2010, its incidence has reached 56/100,000 in the developed regions along the coast and showed a trend of onset in younger patients. In Shanghai, the incidence of colorectal cancer is increasing by 4.2% per year on average, it has taken the 2nd place among common malignancies and accounted for 13.08% of all the malignancies.Chemotherapy is the main therapy for advanced colorectal cancer. The chemotherapeutic agents commonly used in clinical practice include 5FU/LV, capecitabine, irinotecan and oxaliplatin, and their combination regimen are mostly used for chemotherapy.In recent years, the monoclonal antibody bevacizumab targeting vascular endothelial growth factor (VEGF), the monoclonal antibody cetuximab and panitumumab targeting EGFR have been combined with chemotherapy respectively as the first-line therapy for patients with advanced colorectal cancer, and have significantly improved the therapeutic outcome in these patients[1-2].The 2nd-line chemotherapy is the main therapy for patients with colorectal caner who have failed with the 1st-line therapy, mechanisms of the 2nd-line therapeutic agents variey, selection of treatment is mainly dependent on the type of tumor, duration of chemotherapy as well as the side and toxic effects of the drug. The efficacy and safety of some drugs still need to be further evaluated [2-3].The selective kinase inhibitor of VEGFR from Bayer healthcare, regorafinib was approved by FDA for treatment of advanced colorectal cancer after failure of the 2nd-line chemotherapy in 2012, its main endpoint, the median survival was prolonged by 1.4 month as compared with placebo (6.4m vs 5.0m), and the hazard ratio was 0.773 (p=0.0051) [4].

Angiogenesis is the most critical process in the occurrence and progression of malignant tumors.The study found the angiogenesis in tumor area provided nutrients for the tumor and took away metabolites, and tumor cell could transfer to other sites via the new blood vessels.Thus, effective inhibition of the angiogenesis in tumor area could inhibt the growth of tumor cells, and reduce the occurrence of metastasis.Currently, anti-tumor angiogenesis has become the most promising novel strategy for treatment of tumors.Development of tumor angiogenesis is associated with multiple vessel-related factors, rapidly growing tumor cells secret various vascular growth factors under anoxic condition and stimulate tumor angiogenesis.One of the important growth factors is vascular endothelial cell growth factor (VEGF).VEFR is currently found to be one of the main inducing factors related with tumor angiogenesis, thus VEGF/VEGFR signaling pathway is considered as one of the

most promising targets in molecular targeted therapy of tumors[5].

In recent years, the successful advent of the monoclonal antibody bevacizumab for treatment of advanced colorectal cancer and breast cancer, Sunitinib (Sutent) for treatment of renal carcinoma, sorafenib (Nexavar) for treatment of hepatic carcinoma and renal carcinoma, and other small molecular targeted antitumor agents in succession not only exhibited an astonishing market outlook, but also brought the traditional therapy of tumors towards individualized comprehensive therapy, allowing numerous patients to benefit from control of tumor and prolonged survival. The giant success of VEGF highly selective monoclonal antibody, bevacizumab in clinical practice fully demonstrated the effectiveness of this target and its importance in R & D of new drugs.Currently, the available new drug Sunitinib and sorafenib in the market are classified as inhibitors for multi-target small molecular kinase including vascular endothelial cell growth factor receptor (VEGFR). These drugs inhibit tumor angiogenesis kinase and cancer cell growth signaling kinase at the same time. Excessive inhibition of kinase, however would cause great toxic and side effedcts.Research and development of the selective 2nd generation of VEGFR have become the hot spot globally.

Fruquintinib, researched and developed by Hutchison Medi Pharma (Shanghai) Co., Ltd., is a potent small molecular compound using VEGFR kinase family as the target and closely related with angiogenesis, with complete proprietary intellectual property rights.Fruquintinib mainly acts on the transmembrane receptor family (VEGFR1, 2 and 3) on vascular endothelial cells (vascular endothelial growth factor receptor, VEGFR) and has a very high selectivity for kinase.The 50% inhibitory concentration (IC50) was measured to be 35nM, 33nM and 0.5nM for VEGFR1, 2 and 3, respectively.However, Fruquintinib had no inhibitory activity on a variety of kinases related with cell cycle or proliferation, including cyclin-dependent kinase (CDK1, 2, 5), epidermal growth factor receptor (EGFR) and mesenchymal-epithelial transition factor (c-Met) (IC50>3μM). Fruquintinib had no obvious inhibitory activity on platelet-derived growth factor receptor (PDGFRβ) kinase as well (IC50>1 μM), thus demonstrating a good selectivity at molecular level.

1.2 Fruquintinib

1.2.1 Preclincial evidence of Fruquintinib

Fruquintinib was very weak in direct killing of 13 kinds of cells including primary HUVEC cell (IC50≥30 μM, 18.7 μM for primary HUVEC cell).As compared with VEGF-dependent HUVEC proliferation (IC50 1.7 nM only), the difference between them was more than 10,000 times.Fruquintinib had a potent efficacy and low toxicity at enzymatic and cellular level.

In human colon cancer HT-29, human non-small cell lung cancer NCI-H460 and human renal carcinoma Caki-1 subcutaneous grafting models in nude mice at the dose of 0.77, 1.92, 4.8 and 12 mg/kg (2.5 times ascending) qd po for consecutive 3 weeks, Fruquintinib showed a dose-dependent inhibitory effect on tumor growth.And in human malignant melanoma A375 model, human pancreatic cancer model BXPC-3、pancreatic cancer model

Miapaca, hepatic carcinoma Bel-7402 and other refractory tumor models, Fruquintinib showed a significant inhibition of tumor growth at the dose of 1.5, 5 and 15 mg/kg daily.Human gastric cancer BGC-823 model was the most sensitive to Fruquintinib, the tumor growth could almost be completely inhibited at the dose of 2 mg/kg per day.

1.2.2 Results of phase I clinical trial of Fruquintinib (preliminary results as of October 30, 2012)

1.2.2.1 Maximum tolerated dose (MTD) of Fruquintinib in subjects with advanced malignant solid tumor

In accordance with modified Fibonacci method, the dose groups of Fruquintinib 1mg, 2mg, 4mg, 5mg and 6mg were designed in the trial.According to the study design, the grade 4 hematological toxicity, grade 3 neutropenia with fever, grade 3 thromocytopenia with hemorrhage and/or > grade 3 non-hematological toxicity during the first cycle of treatment (28 days) were judged to be dose limiting toxicity (DLT), however, grade 3 asthenia, diarrhea, vomiting, headache and hypertension that were recovered after standard care for 7 days were excluded.

No DLT occurred after evaluation of the safety and pharmacokinetics of Fruquintinib single and consecutive administration for 4 weeks in three dose groups (1.0mg, 2.0mg and 4.0mg) according to dose escalation.In the subsequent 6.0mg dose group, 2/3 subjects were judged to have DLT, who had grade 3 hand-foot syndrome on Day 14 and Day 28 respectively.Thus, the dose escalation was discontinued and 3 subjects were added for the tolerability test for the intermediate dose of 5.0mg, as to determine the MTD.2 subjects in 5.0mg dose group were judged to have DLT and had grade 3 platelet count decreased with hemorrhagic tendency and grade 3 hand-foot syndrome on Day 24 and Day 28, respectively. The toxicity in both of them was recovered to grade 1 within one week after discontinuation of the drug.Therefore, 3 subjects were added for the tolerability test in the previous 4.0mg dose group. 1/3 subjects added had grade 3 hyperbilirubinemia on Day 28, which was judged to be DLT.Multi-dose of 4.0mg QD was thus determined to be the maximum tolerated dose of Fruquintinib in treatment of advanced malignant solid tumors in this trial.

Meanwhile, different administration modes of 3-week on/1-week off were also explored in treatment of advanced solid tumors, and the judgement window of DLT was the first two cycles of treatment (56 days).The safety and pharmacokinetic evaluation of 5.0mg and 6.0mg, 3-week on/1-week off was completed totally.No DLT occurred in 5.0mg dose group (3 subjects), 1/3 subjects in 6.0mg dose group had grade 3 asthenia on Day 28 that was judged as DLT; no DLT occurred in the subsequently added 3 subjects in this dose group.Then another 5 subjects were added in 5.0mg dose group, where no DLT occurred.As the administration modes of 3-week on/1-week off, 5.0mg and 6.0mg were both tolerable doses, and higher doses were not explored due to certain risks.

Exposure in each dose group was provided in Table 1, and list of subjects with DLT was provided in Table 2.

Table 1. Dose exposure

Dose Group	No of subjects	Number of subjects with DLT
1.0mg QD	1	0
2.0mg QD	3	0
4.0mg QD	16	1
5.0mg QD	3	2
6.0mg QD	3	2
5.0mg, 3-week on/1-week off	8	0
6.0mg, 3-week on/1-week off	6	1
Total	40	6

Table 2 List of subjects with DLT

Subject Number	*Dose Group*	*Time of DLT*	*Administration day when DLT occurred*	*Completion of the study*	*Correlation*	*Description in detail*
008	6.0mg QD	2011-11-28	D28	*Yes*	Definitely related	Grade 3 hand-foot syndrome
009	6.0mg QD	2011-11-21	D14	Premature withdrawal	Definitely related	Grade 3 hand-foot syndrome
011	4.0mg QD	2012-01-04	D28	*Yes*	Possibly unrelated	Grade 3 hyperbilirubinemia *
014	5.0mg QD	2012-02-27	D28	*Yes*	related	Grade 3 hand-foot syndrome
015	5.0mg QD	2012-02-24	D24	*Yes*	Possibly related	Grade 3 thrombocytopenia with hemorrhagic tendency.
105	6.0mg, 3-week on/1-week off	2012-06-27	21 days on/1 week off	*Premature withdrawal*	Possibly related	Asthenia

* Note: Subject 011 had grade 3 hyperbilirubinemia and grade 3 elevation of aminotransferase on Day 28 after multiple doses, the grade 3 hyperbilirubinemia was judged by investigator to be possibly related with the study drug and as DLT; in the following further examination, treatment and follow-up, it was found the hyperbilirubinemia was related with the patency of biliary drainage and considered to be caused by biliary tract obstruction and possibly not related with the study drug but with the disease.

1.2.2.2 Safety and tolerability

Adverse events:

1) **Occurrence of adverse events in dose escalation period (including the washout period) for single dose (n＝13)**

Except 1.0mg dose group, adverse events occurred in all the dose groups, most

of which were unrelated or possibly unrelated with the study drug.The adverse events related with the study drug were diarrhea (15.38%, 2), decreased white blood cell count (7.69%, 1) and hemoglobin decreased (7.69%, 1).All of them were grade 1-2 and recovered in the washout period with no treatment.

2) Occurrence of adverse events in the DLT window period after multiple doses

✧ Occurrence of adverse events in the first 28 days of dose escalation tolerability test for consecutive administration (n＝26)

The incidence of adverse event was 100% in each dose group, most of which were related with the study drug and recovered or relieved after symptomatic and supportive treatment.The most common adverse event was hand-foot syndrome (42.3%), followed by hypertension and elevated TSH (each 38.4%), stomatitis (34.6%), diarrhea and proteinuria (each 30.7%), leukocytopenia and hoarseness (each 26.9%), thromocytopenia (19.2%), asthenia and rashes (each 15.4%), most of which were related with the study drug. The incidence of the other adverse events was low and less than 10%.

The majority of adverse events were graded as NCI 1-2.There were a total of 11 grade 3 adverse events as following respectively: hand-foot syndrome 3 (11.5%), diarrhea 3 (11.5%), hypertension 2 (7.7%), blood phorsphorus decreased 1 (3.8%), platelet count decreased 1 (3.8%), hemorrhage of artificial anus 1 (3.8%). As judged by investigators, the causality of the above grade 3 adverse events with the study drug could not be excluded.No grade 4-5 adverse events occurred in each dose group.

No serious adverse event occurred in the dose escalation tolerability test for consecutive administration for 28 days, and only 1 subject (No. 009 in 6.0mg group) interrupted the dose due to DLT (grade 3 hand-foot syndrome) after consecutive administration for 13 days.

✧ Occurrence of adverse events in the first 56 days in the expansion part of administration for 3-week on/1-week off (n＝14)

The incidence of adverse events was 100% in the two dose groups, most of which were related with the study drug and recovered or relieved after symptomatic and supportive treatment.The most common adverse event was hand-foot syndrome (13, 92.8%), followed by stomatitis (8, 57.1%), leukocytopenia (7, 50%), elevated TSH (6, 42.8%), hypertension, hoarseness and asthenia (each 4, 28.6%), elevation of total bilirubin and pain (3, 21.4%), change of nail color (2, 14.3%), most of which were related with the study drug. The incidence of the other adverse events was low and less than 10%.

The majority of adverse events were graded as NCI 1-2.There were a total of 4 grade 3 adverse events, as below: asthenia 1, small intestinal obstruction 1, hypertension 1 and thrombocytopenia 1.Except the thrombocytopenia that could not be excluded to be associated with the previous chemotherapy with mitomycin, the causality of all the above grade 3 adverse events with the study drug could not be excluded as judged by investigators.No grade 4-5 adverse events occurred in each dose group.

A total of 1 DLT and 1 SAE occurred in the first 56 days in the expansion part of administration for 3-week on/1-week off.1 subject in 6mg dose group (No. 105) had grade 3 asthenia after consecutive administration for 21 days that was not relieved in the discontinuation period whilst symptomatic treatment was given, thus it was judged to be DLT on Day 28.Meanwhile, small intestinal obstruction occurred 14 days after discontinuation of the drug, which was reported as SAE.It was judged by the investigator to be possibly related with the study drug.

In 5.0mg dose group, the most common adverse events for administration for 3-week on/1-week off (n=8) were hand-foot syndrome (8), stomatitis (5), leukocytopenia (4), hoarseness (3), T wave change in ECG (2), elevation of serum thyroid-stimulating hormone (2), asthenia (2) and thrombocytopenia (2).Except the grade 3 thrombocytopenia that could not be excluded to be associated with the previous chemotherapy with mitomycin in one subject (No. 110), all the events were grade 1-2.No DLT or SAE occurred.

3) Occurrence of adverse events throughout the trial (n=40)

Throughout the trial was defined as the time from the informed consent of the 1st subject on January 26, 2011 to this statistical analysis and summary by October 30, 2012.The incidence of adverse events was 100% for each dose group throughout the trial, most of which were related with the study drug.The majority of them were recovered after symptomatic and supportive treatment, but still lasted in only a few patients on medication, for example, hypertension, stomatitis, hoarsenss, hand-foot syndrome or elevation of serum thyroid-stimulating hormone, which are under onging follow up and observation.

The most common adverse event throughout the trial was hand-foot syndrome (75%), followed by elevation of serum thyroid-stimulating hormone and stomatitis (each 50%), hypertension (42.5%), decreased white blood cell count (40%), proteinuria (37.5%), hoarseness, ECG abnormality and asthenia (each 35%), rashes and diarrhea (30%), thrombocytopenia and pain (27.5%), elevated aminotransferase and neutropenia (each 17.5%), elevated bilirubin (15%), edema, cough, change of nail color and blood phorsphorus decreased (each 12.5%).They were mostly related with the study drug. The incidence of the other adverse events was low and less than 10%.

The majority of adverse events were graded NCI 1-2.There were a total of 36 grade 3 adverse events, most of which occurred in 4.0mg and above dose groups, including: hand-foot syndrome 6 (15%), hypertension 6 (15%), diarrhea 3 (7.5%), prolonged menstrual period 3 (7.5%), platelet count decreased 3 (7.5%), proteinuria 2 (5%), elevated aminotransferase 2 (5%) and asthenia 2 (5%); additionally, grade 3 blood phorsphorus decreased, hyperbilirubinemia and blood alkaline phosphatase increased one each (2.5%) occurred in 4.0mg dose group, grade 3 blood sodium decreased and blood chloride decreased one each (2.5%) occurred in 5.0mg dose group, grade 3 hemorrhage of artificial anus 1 (2.5%) occurred in 6.0mg dose group, and grade 3 small intestinal obstruction 1 (2.5%) occurred in 6.0mg 3-week on/1-week off group.≥ grade 4 adverse events included

grade 4 coma and grade 5 death onc each (2.5%) in 2.0mg dose group (No. 003)； grade 4 bilirubin increased one (No. 011) (2.5%), grade 5 death onc (No. 013) (2.5%) and grade 4 thrombocytopenia one (No. 022) (2.5%) in 4.0mg dose group.As analyzed and judged by investigators, the grade 4 bilirubin increased in No. 011 subject and death in No. 013 subject were possibly unrelated with the study drug among the grade 3-5 adverse events, whereas the causality of the others with the study drug could not be excluded.Occurrence of > grade 3 adverse events in each dose group was provided in Table 3.

In the 5.0mg 3-week on/1-week off group, the adverse events occurred that were related with the study drug were mostly grade 1-2, the common adverse events included hand-foot syndrome (8), stomatitis (6), leukocytopenia (5), ECG abnormality (4), hoarseness (3), asthenia (3), elevation of serum thyroid-stimulating hormone (3), paronychia (2), diarrhea (2), change of nail color (2), irregular menses (2), thrombocytopenia (2), elevated aminotransferase (2) and bilirubin increased (2).There were a total of 5 grade 3 adverse events, including hand-foot syndrome (1), hypertension (1), thrombocytopenia (1) and irregular menses (2), which were judged by investigators to be related or possibly related with the study drug.No grade 4 or above adverse events or SAEs occurred.

The dose limiting toxicity for this study included hand-foot syndrome (3), and thrombocytopenia with hemorrhagic tendency, hyperbilirubinemia and asthenia (1 each).

Serious adverse events occurred in a total of 7 patients during the study,including acute hepatic impairment in one patient (grade 4 hyperbilirubinemia and grade 3 elevated aminotransferase), which was initially judged to be related with the study drug.Multiple hepatic metastases and intrahepatic bile duct obstruction were found in the subsequent CT, ERCP and surgical procedure, the grade 4 hyperbilirubinemia was finally considered by the investigator to be resulted from the progression of disease and possibly not related with the study drug, whilst the grade 3 elevated aminotransferase was possibly related with the study drug.1 subject had coma and discontinued the drug for suspected brain metastasis as central nervous system (CNS) symptoms occurred during the treatment, coma occurred on Day 4 after discontinuation of the drug, and the subject died of respiratory failure later.As no defined brain metastasis was diagnosed in the brain MRI, the relationship with the study drug could not be excluded.It was worth noting that no hemorrhage, infarct and reversible posterior leukoencephalopathy (RPLS) were found in the brain MRI, which was different with the CNS adverse events observed for similar anti-VEGFR targeted therapeutic agents, thus it was worth of further exploration of the relationship between this adverse event and the study drug.1 subject had thrombocytopenia that was judged by the investigator to be related with the study drug and recovered after treatment.1 subject had small intestinal obstruction that was judged by the investigator to be possibly related with the study drug and eventually not recovered due to progression of concurrent disease and poor state.Additionally, there were 2 upper respiratory tract infections and 1 hydrocele of tunica vaginalis, which were judged by the investigator to be possibly unrelated with the study

drug.List of patients with SAEs was provided in Table 4.

There were 12 adverse events leading to dose reduction or interruption; 9 leading to permanent discontinuation of the drug throughout the trial, including 1 adverse event in 2.0 mg dose group, 2 in 6.0mg dose group, 1 in 5.0mg 3-week on/1-week off group, 1 in 6.0mg 3-week on/1-week off group, and 4 in 4.0mg (MTD) dose group.List of patients with dose interruption and permanent discontinuation of the drug due to adverse events was provided in Table 5-6 in detail.8 subjects received the drug for ≥8 months during the trial (as of October 30, 2012), including 8 months in subject No. 002 (advanced rectal cancer), 10 months in No. 005 (advanced colon cancer), 11 months in No. 006 (advanced gastric cancer), 8 months in No. 012 (breast cancer), No. 016(non-small cell lung cancer) and No. 019 (colorectal cancer), more than 8 months in No. 017 (colorectal cancer) and No. 018 (adrenal pheochromocytoma), and were currently still on-treatment and followed up, which was indicative of a good safety and tolerability of long-term use of the study drug.

Table 3. List of > grade 3 adverse events in each dose group

AE term	CTC grade	2mg	4mg	5mg	6mg	5mg 3-week on/1-week off	6mg for 3-week on/1-week off	Total
Hand-foot syndrome	3	0	2	1	2	1	0	6
Hypertension	3	0	4	0	0	1	1	6
Thrombocytopenia	3	0	1	1	0	1	0	3
	4	0	1	0	0	0	0	1
Diarrhea	3	0	2	0	1	0	0	3
Irregular menses	3	0	1	0	0	2	0	3
Proteinuria	3	0	1	1	0	0	0	3
Transaminase increased	3	0	2	0	0	0	0	2
Asthenia	3	0	1	0	0	0	1	2
Upper respiratory tract infection	3	0	2	0	0	0	0	2
Hyperbilirubinaemia	3	0	1	0	0	0	0	1
	4	0	1	0	0	0	0	1
Blood phosphorous decreased	3	0	1	0	0	0	0	1
Phosphatase alkaline increased	3	0	1	0	0	0	0	1
Blood sodium decreased	3	0	0	1	0	0	0	1
Blood chloride decreased	3	0	0	1	0	0	0	1
Hemorrhage of artificial anus	3	0	0	0	1	0	0	1

AE term	CTC grade	2mg	4mg	5mg	6mg	5mg 3-week on/1-week off	6mg for 3-week on/1-week off	Total
Small Intestinal Obstruction	3	0	0	0	0	0	1	1
Coma	4	1	0	0	0	0	0	1
Death	5	1	1	0	0	0	0	2
Total	3	0	19	5	4	5	3	36
	4	1	2	0	0	0	0	3
	5	1	1	0	0	0	0	2

Table 4. List of patients with SAE

Subject No.	Dose Group	AE term (PT)	NCI-CTC grade	Relatedness to the study drug	Outcome
003	2mg	Coma	4	Possibly related	AE ongoing
		Death	5	Possibly related	AE ongoing
011	4mg	Hyperbilirubinaemia	4	Possibly unrelated	AE ongoing
013	4mg	Upper respiratory tract infection	3	Possibly unrelated	AE resolved
		Death	5	Possibly unrelated	AE ongoing
017	4mg	Acquired hydrocele of tunica vaginalis	3	Possibly unrelated	AE ongoing
022	4mg	Thrombocytopenia	4	Related	AE resolved
026	4mg	Upper respiratory tract infection	3	Possibly unrelated	AE ongoing
105	6mg 3-week on/1-week off	Small intestinal obstruction	3	Possibly related	AE ongoing

Table 5. List of subjects with AE leading to dose interruption

Subject No.	Dose Group	AE term (PT)	NCI-CTC grade	Relationship with the study drug	Outcome
13	4.0mg	Proteinuria	3	Related	AE resolved
		Pericardial effusion	2	Possibly unrelated	
14	5.0mg	Hand-foot syndrome	3	Related	AE resolved
		Platelet count decreased	2	Related	AE resolved
		Blood sodium	3	Possibly related	AE resolved

Subject No.	Dose Group	AE term (PT)	NCI-CTC grade	Relationship with the study drug	Outcome
		decreased			
15	5.0mg	Platelet count decreased	3	Possibly related	AE resolved
		Proteinuria	3	Related	AE resolved
17	4.0mg	Hand-foot syndrome	3	Related	AE resolved
		Acquired hydrocele of tunica vaginalis	3	Possibly unrelated	AE ongoing
19	4.0mg	PROTEINURIA	2	Possibly related	AE ongoing
21	4.0mg	Hand-foot syndrome	3	Possibly related	AE resolved
		Mouth ulcer	2	Possibly related	AE resolved
22	4.0mg	Thrombocytopenia	2	Related	AE ongoing
26	4.0mg	Upper respiratory tract infection	3	Possibly unrelated	AE ongoing
101	5mg 3-week on/1-week off	Hand-foot syndrome	2	Related	AE resolved
102	5mg 3-week on/1-week off	Hand-foot syndrome	3	Related	AE resolved
103	5mg 3-week on/1-week off	Thrombocytopenia	2	Related	AE resolved
106	6 mg 3-week on/1-week off	Stomach discomfort	2	Possibly related	AE resolved

Table 6. List of subjects with AE leading to permanent discontinuation of the drug

Subject No.	Dose Group	AE term (PT)	Start Date	End date	NCI-CTC grade	Relationship with the study drug	Outcome
3	2.0mg	Restlessness	2011-5-13	-	3	Possibly unrelated	AE ongoing
3	2.0mg	Coma	2011-5-14	-	4	Possibly related	AE ongoing
3	2.0mg	Hiccups	2011-5-14	-	2	Possibly unrelated	AE ongoing
3	2.0mg	Petit mal epilepsy	2011-5-16	2011-5-16	2	Possibly unrelated	AE resolved
3	2.0mg	Death	2011-5-21	2011-5-21	5	Possibly related	Death
8	6.0mg	Hand-foot syndrome	2011-11-28	2011-12-12	3	Definitely related	AE resolved
9	6.0mg	Hand-foot syndrome	2011-11-21	2011-12-11	3	Definitely related	AE resolved

Subject No.	Dose Group	AE term (PT)	Start Date	End date	NCI-CTC grade	Relationship with the study drug	Outcome
11	4.0mg	Hyperbilirubinaemia	2012-1-4	-	3	Possibly unrelated	AE ongoing
11	4.0mg	Alanine aminotransferase increased	2012-1-4	2012-1-19	3	Possibly related	AE resolved
11	4.0mg	Aspartate aminotransferase increased	2012-1-4	2012-1-19	3	Possibly related	AE resolved
11	4.0mg	Blood alkaline phosphatase increased	2012-1-4	-	3	Possibly related	AE ongoing
13	4.0mg	Asthenia	2012-5-4	2012-5-10	3	Possibly related	AE resolved
22	4.0mg	Thrombocytopenia	2012-5-3	2012-5-12	4	Possibly related	AE resolved
26	4.0mg	pulmonary infection	2012-9-13	-	3	Possibly unrelated	AE ongoing
105	6.0mg 3-week on/1-week off	Asthenia	2012-6-21	-	3	Possibly related	AE ongoing
110*	5.0mg 3-week on/1-week off	Thrombocytopenia	2012-8-24	2012-8-30	3	Possibly related	AE resolved

* Note: No. 110 subject had grade 3 thrombocytopenia on Day 11 after multiple doses, as the incomplete recovery of the hematological toxicity from the last chemotherapy could not be excluded (grade 3 thromocytopenia with hemorrhagic tendency could appear after use of mitomycin) (more than 4 weeks but less than 6 weeks from the last chemotherapy), the association with the study drug could not be excluded as well.

Laboratory examination, vital sign and physical examination:

Abnormal changes occurred in each parameter of the complete blood cell count, routine urinalysis, fecal occult blood, coagulation function, thyroid function and immunological test in the laboratory examinations for partial patients during the trial, and part of them were judged to be adverse events. Abnormal change of vital signs was mainly characterized by increased blood pressure.The blood pressure was ranged from 85～168/48～130 mmHg for each subject; the heart rate was ranged from 45～129 beats/min; the respiratory frequency was ranged from 9～36/min; and the temperature was ranged from 36.0℃～38.3℃.Change of body weight was within 10% for most subjects, and only 2 subjects in 4.0mg

dose group had change of body weight of more than 10%.Part of the subjects had abnormal findings during the monitoring of echocardiography, however, these findings were judged to be non-adverse events.The ECOG score was decreased from baseline (≥2) in 4 subjects.

1.2.2.3 Observation of clinical efficacy:

Efficacy in ITT population (n=40) as below：

Overall efficacy: 13 PRs (not confirmed for 1), 15 SDs, 6 PDs, 6 not evaluable. The objective response rate was 32.5% and the disease control rate (PR+SD) was 70%.

In the daily administration dose group: 1 subject in 1.0mg group had SD, 2 subjects in 2.0mg group had SD, 13/15 subjects in 4.0mg group had SD (≥8 weeks) or above (7 PRs and 6 SDs), 3 subjects in 5.0mg group had SD or above (1 PR and 2 SDs), 1 subject in 6.0mg group had PD, 4 subjects withdrew from the trial prematurely for toxicity and were not evaluable for efficacy.

In the administration for 3-week on/1-week off group: 6/8 subjects in 5.0mg group had SD (≥8 weeks) or above (4 PRs and 2 SDs), 3/6 subjects in 6.0mg group had SD or above (1 PR and 2 SDs).2 subjects withdrew from the trial prematurely for toxicity and were unevaluable for efficacy.

Efficacy in the 34 evaluable patients as below：

Evaluable patients included those who received the efficacy evaluation after completion of at least two cycles of treatment and those who had progression of disease at any time.

Overall efficacy: 13 PRs (not confirmed for 1), 15 SDs, 6 PDs. The objective response rate was 38.23% and the disease control rate (PR+SD) was 82.3%. 1 subject receiving efficacy evaluation in 1.0mg dose group had SD, 2 subjects receiving efficacy evaluation in 2.0mg group had SD, 13/15 subjects receiving efficacy evaluation in 4.0mg group had SD or above (7 PRs and 6 SDs), 3 subjects receiving efficacy evaluation in 5.0mg group had SD or above (1 PR and 2 SDs), and 1 subject receiving efficacy evaluation in 6.0mg group had PD.6/7 subjects receiving efficacy evaluation in 5.0mg 3-week on/1-week off group had SD or above (3 PRs, one uncomfirmed PR, 2 SDs), 3/5 subjects receiving efficacy evaluation in 6.0mg 3-week on/1-week off group had SD or above (1 PR and 2 SDs).

Efficacy in 4mg dose group (MTD) as below:

Throughout the trial, among the 15 subjects with evaluable tumor in 4.0mg dose group, 7 were evaluated as PR, 6 were SD and only 2 were PD, the objective response rate was 46.66% and disease control rate (PR+SD) was up to 86.66%.

Efficacy in 5mg 3-week on/1-week off group as below：

Throughout the trial, in the 7 subjects with evaluable tumor in 5mg 3-week on/1-week off group, there were 4 PRs (not confirmed for 1 as the conformatory period was not reached yet), 2 SDs and only 1 PD. The objective response rate was 57.14% and disease control

rate (PR+SD) was up to 85.71%.

1.2.2.4 Recommended dose for phase Ib clinical trial

In combination with the clinical tolerability test and pharmacodynamic results for Fruquintinib, the recommended doses for the phase Ib clinical trial are 4.0mg qd daily and 5.0mg qd 3-week on/1-week off.

1.2.2.5 Pharmacodynamics results

Preliminary results from the phase I pharmacokinetic clinical study of Fruquintinib in treatment of advanced solid tumors:

The very low clearance rate and low tissue distribution of Fruquintinib in humans allowed a high plasma exposure after oral administration even at a low dose, and Fruquintinib had a long elimination phase half-life (about 42 hours).Fruquintinib was well and rapidly absorbed after oral administration of one single dose of 1mg to 6mg, which was in line with the linear pharmacokinetic profile, and its exposure was linearly increased with the increase of dose.A steady state was considered after consecutive administration of Fruquintinib for 14 days; the exposure at the steady state was linearly increased with the increase of dose over the range of 1mg - 6mg; the long half-life of Fruquintinib supported the administration method of once daily, and this method could make the plasma drug concentration at steady state fluctuate within a very small range but produce about 3～4 of accumulation at steady state, which was resulted from the linear superposition of the effect after multiple doses. This method was still in accordance with the linear pharmacokinetic profile after multiple doses.

Study on the effect of food on the pharmacokinetics of Fruquintinib (preliminary results):

It was preliminarily found in one monocentric, randomized, open-label, single-dose, two-period, cross-over clinical study on the effect of food on the pharmacokinetics of Fruquintinib capsule in healthy volunteers that food had no statistically significant effect on the exposure (AUC) and peak concentration (Cmax) of 4mg Fruquintinib capsule after single dose in healthy males, however, the time to reach the peak (Tmax) was delayed by food.

1.2.3 Results from long-term toxicology study of Fruquintinib

1.2.3.1 Long-term toxicity study in SD rats given Fruquintinib orally for 26 weeks followed by 4-week recovery period

Under the conditions in this study, SD rats were given Fruquintinib orally for consecutive 182 days at dose of 0 (excipient), 0.5/0.25, 1.5/0.75, 5/3/1.5 and 10/6 mg/kg. Death could be caused at the dose of no less than 1.5/0.75 mg/kg Fruquintinib. The main toxicities observed at the dose of no less than 0.5/0.25 mg/kg included anodontia, perinasal pollution, eye secretion, watery stool, reduced activity, slowed or reduced body weight gain, decreased food intake, increased ALT, ALP, CHOL, TG and BUN, decreased GLU,

hypoproteinemia, positive urine protein and white blood cell, bone marrow proliferation inhibition, bile duct dilation/thickening, pathological changes of liver, kidney, adrenal gland, thymus, spleen and femur. The above toxicities could not be completely recovered 4 weeks after discontinuation of the drug. The main target organs of toxicity were liver, kidney, adrenal gland, thymus, spleen, bone marrow (sternum) and femur. No drug accumulation was seen after repeated doses on Day 91 and 182. The lowest observed adverse effect level (LOAEL) was 0.5/0.25 mg/kg (C_{max} and $AUC_{0\text{-}24\ hr}$ on D182 was 327 ng/mL and 3676 hr*ng/mL, respectively).

1.2.3.2 Long-term toxicity study in Beagle dogs given Fruquintinib (drug product) orally for 39 weeks followed by 4-week recovery period

Under the conditions in this study, Beagle dogs were given Fruquintinib orally for consecutive 39 weeks at dose of 0.01, 0.03, 0.1/0.06 and 0.2/0.12 (dose reduced to 0.06 and 0.12 mg/kg from Day 15 of administration, respectively), the recovery period was 4 weeks. 0.2/0.12 mg/kg Fruquintinib could lead to death, and the main toxicities included reduced activity, watery stool, red feces (hemafecia), decreased food intake, decreased body weight, slowed HR, decreased WBC, hypoproteinemia, hepatotoxicity (vacuolation, swelling of hepatocytes, granulomatous inflammation, hepatocyte pigmentation and increased Kupffer cells), nephrotoxicity (vacuolation of tubular epithelial cells and positive urine protein), immunosuppression (thymus atrophy, decreased lymphocytes at the marginal zone of splenic white pulp, decreased lymphocytes in the cortex of mesenteric lymph nodes) and thickening of femoral metaphyseal growth. The main target organs of toxicity were gastrointestinal tract, liver, kidney, immune system (thymus, spleen and lymph node) and femur. No significant gender difference or drug accumulation was seen after repeated doses. The no observed adverse effect level (NOAEL) was 0.03 mg/kg ($AUC_{0\text{-}24hr}$ was 343±55 hr*ng/mL on Day 273), corresponding to 0.4 of the equivalent effective dose in dogs.

2 Study objectives

2.1 Primary objectives

To compare the safety and tolerability of two different administration methods of Fruquintinib (4mg qd daily vs. 5mg qd 3-week on/1-week off) in treatment of patients with advanced colorectal cancer who have failed with standard care; and to further observe and evaluate the safety and efficacy (progression-free survival, PFS) of Fruquintinib 5mg qd 3-week on/1-week off in treatment of patients with advanced colorectal cancer who have failed with standard care during expansion period.

2.2 Secondary objectives

- To compare the objective response rate, disease control rate (DCR), duration of response (according to RECIST 1.1 criteria), progression-free survival (PFS) and overall survival (OS) between two different administration methods of Fruquintinib in treatment of patients with advanced colorectal cancer who have failed with standard

care; and to further evaluate the disease control rate (DCR), objective response rate (ORR) and overall survival (OS) using the administration method of 5mg 3-week on/1-week off in treatment of patients with advanced colorectal cancer who have failed with standard care during the expansion period.

- To explore the pharmacokinetic profile of two different administration methods of Fruquintinib in treatment of patients with advanced colorectal cancer who have failed with standard care.

3 Trial Design

3.1 SUMMARY OF EXPERIMENTAL DESIGN

3.1.1 Rationale for Study Design

This is a bicentric, open-label, phase Ib clinical trial comparing the safety and efficacy of two different administration methods of Fruquintinib in patients with advanced colorectal cancer after failure of standard care, as to further determine the recommended dose and administration method for phase II/III clinical studies.

This trial is comprised of two parts: randomized comparison part and expansion part.

Randomized comparison part: patients will be randomized into Group A or B after meeting the screening criteria.

Group A: Fruquintinib 4mg qd po, daily, until progression of disease, unacceptable toxicity or voluntary withdrawal from the trial in advance.

Group B: Fruquintinib 5mg/ qd po, 3-week on/1-week off**,** until progression of disease, unacceptable toxicity or voluntary withdrawal from the trial in advance.

28 days is one cycle of Fruquintinib treatment.Information on the subsequent antitumor therapy will be collected for the patient after progression of disease, unacceptable toxicity or voluntary withdrawal from the trial, and the patient’s survival status will be followed up until death.

Expansion part: the recommended dose for phase II/III studies is determined for the expansion part based on the randomized comparison part.

3.1.2 Rationale for Dose Selection.

(1) Safety dose:

Phase I clinical tolerability study of Fruquintinib: A single dose within the range of no more than 6.0mg could be tolerated, and higher doses were not explored. Multi-doses of Fruquintinib once daily were given for consecutive 28 days in the phase I tolerability clinical trial, the main dose limiting toxicity (DLT) was hand-foot syndrome. Grade 3 hand-foot syndrome limited the subjects to receive 6.0mg dose and **the maximum tolerated dose was 4.0mg.**

Expansion part of administration for 3-week on/1-week off: 5.0mg and 6.0mg could be

tolerated, and higher doses were not explored. Only one DLT of asthenia occurred in 6.0mg dose group.

(2) Efficacy during the whole phase I tolerability trial:

In the 15 evaluable patients in 4.0mg dose group, 13 subjects reached SD or above (7 PRs, 6 SDs ≥8 weeks; patients with partial response had colorectal cancer, gastric cancer, lung cancer and breast cancer) and the objective response rate was up to 46.66%.Although 2 grade 3 hepatic impairment, 2 grade 3 hand-foot syndrome, 1 grade 3 proteinuria, 1 grade 3 asthenia and 1 grade 3 stomatitis that were possibly related with the study drug, and 2 >grade 3 thrombocytopenia that were related with the study drug occurred in this dose group, all the symptoms were resolved or relieved after discontinuation of the drug and active treatment. Therefore, significant antitumor activity and good tolerability were shown in 4.0mg dose group.

In the 7 evaluable patients in 5.0mg 3-week on/1-week off group, 6 subjects reached SD or above, including 4 PRs, the objective response rate was up to 57.14%, and patients with partial response had breast cancer, neuroectodermal malignancy, nasopharyngeal carcinoma and submandibular gland carcinoma.Only 1 grade 3 hand-foot syndrome that was related with the study drug occurred in this dose group, and was resolved after transient discontinuation of the drug and active treatment.Thus a significant antitumor activity and good tolerability were shown in 5.0mg 3-week on/1-week off group as well.

In combination with the preliminary results on safety and efficacy from the tolerability clinical trial and expansion part of Fruquintinib, the recommended doses for phase Ib clinical trial are 4.0mg qd po daily and 5.0mg qd po 3-week on/1-week off.

(3) Preliminary results of the interim analysis of phae Ib trial (as of Sep. 20, 2013):

A total of 40 (1:1) subjects were enrolled in the randomized phase Ib clinical trial comparing two different administration methods of Fruquintinib as 3rd-line or above therapy for advanced colorectal cancer, including 27 males and 13 females; 24 colon carcinoma and 16 rectal cancer.

The main adverse reactions occurred during the trial included hand-foot syndrome, hoarseness, proteinuria, hypertension, stomatitis and asthenia.Grade 3 and above adverse events occurred in more than 10% subjects were hypertension (22.5%) and hand-foot syndrome (17.5%).17 subjects in QD consecutive administration group (n=20) had grade 3 or above adverse events, 6 had grade 3 hand-foot syndrome, 6 had SAE and 7 discontinued the drug permanently for toxicity.8 subjects in administration for 3-week on/1-week off (3/1) group (n=20) had grade 3 or above adverse events, 1 had grade 3 hand-foot syndrome, 4 had SAE and 4 discontinued the drug permanently for toxicity.

Efficacy in both the two groups: the DCR was 76.4%, there were 2 patients with PR+MR and mPFS had not reached 3.7m in QD group (17 evaluable patients); the DCR was 83.3%, there were 3 patients with PR+MR and the mPFS had reached 3.7m in 3/1 group (18

evaluable patients).

Preliminary conclusion: the administration method of 5mg 3/1 was not inferior to that of 4mg QD in terms of efficacy (in particular DCR and PFS), whilst it had a certain advantage over 4mg QD in terms of the occurrence of grade 3 or above adverse events, occurrence of grade 3 hand-foot syndrome, occurrence of SAEs and occurrence of AEs leading to permanent discontinuation of the drug.Therefore, the administration method of 5mg 3/1 was selected for the expansion part of phase Ib trial.

3.2 Number of subjects

The total sample size is 60 subjects.

Randomized comparison part: 20 subjects in each group, randomized (1: 1) into two dose groups.

Expansion part: 20 subjects.

3.3 Study Sites

Two study sites: Fudan University Shanghai Cancer Center, Sun Yat-sen University Cancer Center

4 Study Population

4.1 Target Population

Histologically confirmed advanced colorectal cancer after failure of 2nd-line or above standard therapy

Note: treatment failure is defined as progression of disease.

a: Each line of therapy for progressive disease includes one or multiple drugs used for ≥1 cycle or longer time

b): Early adjuvant/neoadjuvant therapy is allowed.If recurrence or metastasis occurs during or within 6 months after the completion of adjuvant/neoadjuvant therapy, the adjuvant/neoadjuvant therapy is considered as one first-line early systemic chemotherapy for one progressive disease.

c: The early therapy allowed is defined as chemotherapy combined with targeted drugs except VEGFR.

4.2 Enrollment criteria

Subjects can be enrolled only when all the following criteria are met in accordance with the guidance on the antitumor drug clinical trial:

1. Fully understanding of this study and voluntary signature of the informed consent form;
2. Histologically and/or cytologically confirmed advanced colorectal cancer after 2nd-line or above treatment failure;

3. No systemic antitumor treatment or radiotherapy, immunotherapy, biological or hormone therapy in the past 4 weeks; no treatment with VEGFR inhibitor;
4. Age = 18-70 years
5. Body weight >40KG;
6. Performance status score (ECOG score) ≤ 1 (0-1) with no exacerbation within 7 days;
7. Evaluation of cardiac function: left ventricular ejection fraction ≥50% (echocardiography);
8. Definitely measurable lesion;
9. Expected survival > 12 weeks.

4.3 Exclusion Criteria

Patients meeting any of the following conditions must be excluded from this study plan:

1. Absolute neutrophil count (ANC) <1.5×109/L, platelet count <80 ×109/L or hemoglobin < 9g/dL in the laboratory test performed within one week prior to enrollment (based on the normal value at clinical study site);
2. Serum total bilirubin, glutamic-pyruvic transaminase, glutamic-oxaloacetic transaminase and alkaline phosphatase exceeding normal range (based on the normal value at clinical study site); alkaline phosphatase more than 2.5×ULN for patients with liver metastasis;
3. Serum creatinine exceeding the upper limit of normal range (based on the normal value at clinical study site) or creatinine clearance lower than 50ml/min;
4. Systolic blood pressure ≥150mmHg and/or diastolic blood pressure ≥100mmHg regardless of the use of antihypertensive agent;
5. Serum potassium exceeding normal range with clinical significance, regardless of the supplementation of potassium preparation;
6. Serum calcium or magnesium exceeding normal range with clinical significance, regardless of any supplementation;
7. Urine protein 2+ or above, or 24-hour amount of urine protein ≥1.0g/24h;
8. Evidence of intestinal cavity infiltration (with possibility of potential bleeding);
9. Unrecovered toxicity from previous anticancer therapy (＞ Grade 1 toxicity in accordance with CTCAE4.0), not completely recovered from previous surgery or less than 4 weeks from the last surgery;
10. Central nervous system (CNS) metastasis in subject who is considered by investigator not suitable for participation in the study;
11. Clinically uncontrolled active infection, such as acute pneumonia, active phase of

hepatitis B;

12. Dysphagia or known malabsorption to drugs;
13. Current digestive diseases such as duodenal ulcer, ulcerative colitis, or other conditions to possibly cause digestive tract bleeding or perforation as judged by investigators;
14. Bleeding tendency or history of bleeding within 2 months prior to enrollment, regardless of the severity;
15. Development of stroke within 12 months prior to enrollment, including transient ischemic attack;
16. Activated partial thromboplastin time (APTT) or/and INR and prothrombin time (PT) exceeding normal range with clinical significance (based on the normal value at clinical study site);
17. Incomplete healing of skin wound, severe mucosal ulcer or bone fracture;
18. Acute myocardial infarction, severe/unstable angina pectoris or coronary bypass surgery within 6 months prior to enrollment；history of arterial thrombosis or deep venous thrombosis
19. Pregnant or lactating women or positive pregnancy test prior to the first dose of the drug in women with childbearing potential;
20. Unsuitability to participating in the clinical study due to any clinical or laboratory abnormality as considered by investigators;
21. Severe metal or psychological illness, and inadequate compliance with the clinical study could be anticipated;
22. Participation in clinical trial of other drug within the last 4 weeks.

4.4 Criteria for treatment termination

Subjects will prematurely terminate the study and written description will be provided when the following conditions occur.

Criteria on completion of the trial:

1. Progression of disease
2. Death
3. Inability to recover to ≤NCI CTC AE Grade 1 or baseline within 14 days after dose interruption
4. NCI CTC AE Grade 4 (nephrotic syndrome) in routine urinalysis or 24-hour urine protein quantification; CTC AE Grade 4 abnormal hepatic function and hemorrhage

Criteria on premature discontinuation：

1. The subject becomes pregnant.
2. The investigator judges the termination of the study in the subject's best interest;
3. The subject or his/her legal representative requests withdrawal from the study;
4. Subject losses to follow-up
5. Serious violation from the study protocol

4.5 Exclusion Criteria

Never used the drug;

Serious violation from the study protocol judged by investigator and/or sponsor;

All the subjects with at least one medication record should be included in the ITT set.Reasons for exclusion should be described and the CRF/eCRF should be maintained for further check.

4.6 End of Trial

End of the whole trial is defined as 9 months after enrollment of the last patient.

There are a few situations for end of trial for each subject as below:

1 Death

2 The subject or his/her legal representative requests to withdraw from the study and refuses to be followed up.

3 The subject is lost to to follow-up

4 End of the whole trial

The database will be locked after the end of the whole trial.For subjects who have not reached PFS event, the sponsor will continue to provide the product if they are willing to continue the administration after investigator's evaluation, and they will be followed up in accordance with the original schedule for treatment period (including safety evaluation, tumor assessment, drug counting) until progression of disease, death or loss of follow-up, or voluntary withdrawal from the trial.The sponsor is responsible for paying the cost for safety evaluation and tumor assessment but not for collection of data.If any SAE occurs during treatment, one SAE form needs to be filled in by investigators and reported to the sponsor.Survival of all the subjects will not be followed up after the end of the whole trial.

5 Treatment

5.1 Dosage and administration regimen of study drug

Randomized comparison part:

All the subjects will be randomized (1: 1) into Fruquintinib group A (4mg qd daily) and group B (5 mg qd 3-week on/1-week off).

Both of the groups will receive the treatment in a cycle of 4 weeks, until progression of disease, unacceptable toxicityor withdrawal of the informed consent.

Expansion part：

According to the preliminary results of the randomized comparison part, the administration method and recommended dose for phase II/III studies are determined as group B (5 mg qd 3-week on/1-week off) for the expansion part, using 4 weeks as one therapeutic cycle as well, until progression of disease, unacceptable toxicity or withdrawal of informed consent.

5.2 Source, strength and shelf-life of the product

Fruquintinib is provided by Hutchison Medi Pharma (Shanghai) Co., Ltd. Hutchison Medi Pharma (Shanghai) Co., Ltd. sub-contracted Yiling Pharmaceutical Co., Ltd for production and packaging, and performed technical guidance and quality control by itself.

Product Name	Form	Strength	Administration method	Batch No.	Shelf-life
Fruquintinib	Capsules	1.0mg	p.o.	120602	2 years
Fruquintinib	Capsules	1.0mg	p.o.	120901	2 years
Fruquintinib	Capsules	5.0mg	p.o.	120603	2 years
Fruquintinib	Capsules	1.0mg	p.o.	1309051	2 years
Fruquintinib	Capsules	5.0mg	p.o.	1309061	2 years

5.3 Product Labeling

Fruquintinib is provided as the form of capsule in a bottle and maintained at the study site after meeting the acceptance criteria by the user. The study nurse is responsible for storage, maintenance, dispension and counting of the study drug. The sponsor will recover the remaining study drug after the end of study.

Information in the package labelling of the study drug contains the following: study No., storage conditions, contents, dosage, administration method, batch number, shelf-life and standard text “only for clinical studies” and “kept away from reach of children”.

5.4 Drug storage

All of the study drugs must be stored according to specifications, and placed in a safe and sealed place under room temperature or in a shade place.

The storage temperature needs to be noted and kept in appropriate document.

The study drug is only for study and limited to the content specified in this protocol.

5.5 Grouping method and drug dispension

The investigator or study nurse will dispense the study drug as required.The investigator should fill in the Summary Sheet of Drug Dispension promptly and accurately at the

dispension.

5.6 Dosage and administration method

Randomized comparison part:

Group A: dose of Fruquintinib: 4mg qd po (under fasting state), daily.

Group B: dose of Fruquintinib: 5mg qd po (under fasting state), 3-week on/1-week off.

Expansion part: group B is selected for this part in accordance with the results from the randomized comparison part.

5.7 Administration method

It is taken orally with 200ml warm water. The accurate time of actual medication should be recorded each time. Fasting administration is preferred, i.e., 1 hour prior to breakfast or 2 hours after that. Subjects who could not tolerate fasting administration due to gastrointestinal reaction can change to postprandial administration after evaluation by investigators.

Every effort should be made to ensure subject's compliance with the study protocol during the study. The missed dose could be made up before 10pm on that day when the subject misses his/her dose in the morning. However, if the subject misses the scheduled dose and fails to make up the dose on that day, he/she must take the next dose as specified, however, the missed dose is not required to make up.The missed dose must be reported to investigators and recorded on CRF/eCRF. If vomiting occurs within 30 minutes after administration, the administration could be resumed.

Strenuous exercise should be avoided during the trial; use of alcohol, smoking or beverage containing caffeine are forbidden.Intake of grapefruit, shaddock or beverage containing the above fruits should be avoided during the trial.

5.8 Accountability

Registration and record of the study drug need to be managed by designated person.The study site should establish one set of systemic processing and operational procedure as to ensure the receipt of the study drug by one designated person, accurate record of drug dispension, proper use and storage of the study drug.

- Drug registration form; ①type of study drug received from the sponsor; ② dosage form and dose; ③batch number and shelf-life; ④ dosage form, dose, numers and date of drug delivery (signed by both parties) sent to the investigators.
- The investigators should provide the registration form of the study drug: to register the subject's name, package number, time of medication, administration and dosage, signature by the person who administered the drug, and recycle the package.
- Subject's medication, lost and missing dose as well as misuse should be recorded carefully.

- Unused drugs should be stored in accordance with the requirement for storage, and the stored drugs should be inspected if it is in accordance with the requirement by designated person on a regular basis.
- The recovery, registration and record of the remaining drugs should be well performed after the completion of the trial, and the study drugs should be disposed in accordance with GCP requirement.

5.9 Principle and method for management of toxic and side effects after administration

5.9.1 Principle on management of toxic and side effects during the study

If relevant toxic and side effects occur during the study, the management of toxic and side effects, dose interruption and dose reduction need to follow the following principles.Management of the toxic and side effects that are possibly related with Fruquintinib is provided in 5.9.2.

- When grade 4 hematological toxicity or grade 3 non-hematological toxicity and relevant toxic and side effects listed in Table 7 occur, the dose of Fruquintinib should be interrupted, and could be resumed or reduced to the next lower dose if the toxicity could be recovered to grade 1 or below within 14 days; the medication needs to be permanently discontinued if it could not be recovered within 14 days.
- Symptomatic management and treatment could be given actively for controllable nausea, vomiting, asthenia, diarrhea, hypertension, pain, fever with defined cause (eg., infection or tumor) and elevation of alkaline phosphatase in non-hematological toxicity, rather than dose interruption or dose reduction;
- When several AEs occur simultaneously, the dose should be adjusted according to the most severe AE;
- It is inappropriate to discontinue this category of drug for too long, in principle the dose could be resumed when the toxicity is relieved to grade 1;
- Dose adjustment could be performed at any time according to intolerable toxicity in each cycle of administration.The dose must not be uptitrated to its previous level after reduction; 2 dose adjustments are allowed at most (ie., 5mg 3/1 could be downtitrated to 4mg 3/1 for the first adjustment and 3mg 3/1 for the second adjustment; 4mg QD could be downtitrated to 3mg QD for the first adjustment and 2mg QD for the second adjustment)); dose adjustment is not allowed but dose interruption is allowed after the dose is adjusted to 3mg 3/1 or 2mg QD.

5.9.2 Management of the expected adverse events of Fruquintinib

If the toxic and side effect is judged by the investigator to be possibly related with Fruquintinib, management of the effect needs to comply with the principle in Section 5.9.1 and relevant interventions should be given.The phase I tolerability test showed the adverse reactions related with Fruquintinib mainly included hypertension, proteinuria, hand-foot

syndrome, diarrhea, stomatitis and hypothyroidism or elevated TSH.

Management of acute hypertension

Management of mild-to-moderate hypertension in accordance with the following procedure：

1. No treatment is required for asymptomatic transient (<24 hours) increase of diastolic pressure >20mmHg or >150/100mmHg (previously normal range), and study drug is continued.
2. One or more antihypertensive agents could be added for recurrent or persistent (>24 hours) increase of diastolic pressure >20mmHg or >150/100mmHg (previously normal range) or symptoms.Use of the study drug is continued while blood pressure is monitored.
3. If the blood pressure keeps >160/100mmHg for more than one week (previously normal range) after antihypertensive therapy, interruption of Fruquintinib is considered with continuation of antihypertensive therapy and close monitoring.If the hypertension could be recovered to grade 1-2 within 14 days, reuse of Fruquintinib is decided by the investigator together with the sponsor (use of antihypertensive agent will be continued).
4. If the blood pressure increases to >160/100mmHg after the dose of Fruquintinib is resumed, it is decided by the investigator and the sponsor whether to downtitrate the dose of Fruquintinib to the next level.
5. If the blood pressure still increases to >160/100mmHg and lasts for more than one week at the dose level lower than the previous one when in combination with concomitant antihypertensive agent, the study drug should be terminated.

Principle on treatment with mild-to-moderate antihypertensive agent:

- Fruquintinib might lead to elevation of blood pressure;
- Calcium channel antagonist is the preferred drug, and β-receptor blocker is the 2nd choice when use of calcium channel antagonist is contraindicated;
- Angiotensin converting enzyme inhibitor and angiotensin II receptor blocker are mainly used for treatment of hypertension combined with proteinuria
- If the blood pressure is >140/90mmHg after treatment, the dose of drug could be considered to increase to adequate dose and administered in combination with selective β-receptor blocker, thiazide diuretics, angiotensin converting enzyme inhibitor and angiotensin II receptor blocker as required.If the blood pressure is still >140/90mmHg after 24 hours, the dose of combined drug could be considered to increase to adequate dose, or combined with the 3rd antihypertensive agent;

Principle on management of severe hypertension in accordance with the following procedure：

When the diastolic pressure increases to ≥110mmHg or the systolic pressure increases to ≥180mmHg, the management is performed in the following procedure:

1. Interrpution of the study drug and hospitalization;
2. Two antihypertensive agents combined therapy (including one dihydropyridine calcium channel blocker), or adjustment of the antihypertensive agent previously used;
3. Intravenous antihypertensive therapy whilst oral therapy when target organ impairment occurs;
4. Stabilization of the blood pressure, even rescue through request to relevant experts and personnel;
5. When the blood pressure decreases to <140/90mmHg, reuse of the study drug is considered, refer to the aforementioned step 4.

Management of proteinuria

If urine protein 2+ is found in consecutive two urinalyses during the treatment, please collect 24-hour urine.If the urinalysis or 24-hour urine protein quantification is graded as NCI CTC-AE 1, keep close observation; keep close observation for grade 2 and 24-hour urine protein quantification <2g; interrupt the dose for 24-hour urine protein quantification ≥2g, and reduce the dose if it could be recovered to grade 1 or below within 2 weeks; if the urianlysis or 24-hour urine protein quantification is graded as NCI CTC-AE 4 (nephrotic syndrome), discontinue the study drug permanently.

Management of hand-foot syndrome

If the subject develops ≤2 grade hand-foot syndrome, symptomatic treatment could be given.Avoid friction, pressure and contact with heat objects of hands and feet.Keep the skin of hands and feet moist or use urea cream or lanolin cream-containing lotion appropriately, which will be helpful for relieving the symptom and healing of the lesion. Patients with serious symptoms (in particular those with pain) could use ointment for relieving burning and pain or oral voltaren appropriately, which will be helpful for relieving the symptoms.

For subjects with grade 3 or above hand-foot syndrome (serious cutaneous reactions, including exfoliation, blister, hemorrhage, edema with pain, affecting daily life), please interrupt the dose in accordance with the principle in Section 5.9.1. The dose could be resumed or reduced if the toxicity could be recovered to grade 1 or below within 14 days.

Diarrhea

If the subject develops grade 1-2 diarrhea, close observation or use of drugs for improving intestinal function could be given; if grade 3 diarrhea occurs, Imodium or other drugs for improving intestinal function could be given, the toxicity could be recovered generally within 3 days and the dose could be continued; dose interruption is required if it lasts for more than 3 days.

Mucositis oral

If the subject develops grade 1-2 stomatitis (including oral ulcer, mucositis, gingivitis, laryngeal discomfort, angular stomatitis), local antibiotics (including those for anaerobic bacteria) and antifungal drugs, mucosa protective agent and local anesthetics, as well as oral Xiguashuang spray and Yinhuangjiedu tablet could be given, and the administration could be continued.Use of iodine-containing product and long-term use of hydrogen peroxide should be avoided; soft and non-irritable food is proposed in terms of diet, and spicy, acid or irritable food should be avoided.If the patient could not swallow food or liquid diet, parenteral fluid or nutritional support might be needed.

If the stomatitis could not be recovered and affects food intake and body weight after treatment, the dose needs to be interrupted; if it could be recovered to grade 1 within 14 days, continuation of the administration with the original dose or dose reduction could be considered.

Hypothyroidism and elevated TSH

If the subject develops clinically significant hypothyroidism (with or without clinical symptoms), or the TSH keeps increasing with clinical symptoms (with or without decreased T4), he/she is advised to receive hormone replacement therapy.

Platelet count decreased

If the subject develops grade 2-3 thrombocytopenia (<75×10^9/L), he/she is advised to interrupt the dose, and receive recombinant human interleukin -11 for injection for platelet-increasing therapy; apheresis platelets could be transfused when grade 4 toxicity occurs.

For the management of other toxic and side effects, the subject's benefit should be sufficiently considered and proper management and treatment are provided under the above principle on management of toxic reactions.

5.9.3 Provisions on dose adjustment resulted from toxicity：

When drug related toxicity occurs, the treatment of Fruquintinib needs to be performed in accordance with the procedure in Table 7.

Table 7 Provisions on dose adjustment resulted from drug related toxicity

Type of AE and CTCAE grade	Measures	Regimen for dose reduction
Study drug related adverse events	dose interruption until recovery to ≤CTCAE grade 1 or baseline;<br>resume the administration with the reduced dose in accordance with the protocol;<br>If the patient could not be recovered to	administration method of 3-week on/1-week off:<br>downtitrated to<br>4 mg for the first adjustment; to 3 mg for the second adjustment in 5mg dose group;<br>or:<br>administration method of
● Neutropenia<br>CTCAE grade 3 (<1 x10^9/L)		
● Thrombocytopenia<br>CTCAE grade 2 (<75 x10^9/L)		
● Hand-foot syndrome<br>CTCAE grade 3, affecting walking and joint movement		

<table>
<tr><td>Type of AE and CTCAE grade</td><td>Measures</td><td>Regimen for dose reduction</td></tr>
<tr><td>● Hypertension
CTCAE grade 3, still persisting for more than 48 hours after pharmacotherapy</td><td rowspan="6">≤CTCAE grade 1 or baseline within 14 days, the study drug must be permanently discontinued.</td><td rowspan="6">once daily for consecutive administration:
downtitrated to 3 mg for the first adjustment; to 2 mg for the second adjustment in 4mg dose group.
If the dose after the second adjustment could not be tolerated, the study drug must be discontinued regardless of the administration method.</td></tr>
<tr><td>● Stomatitis;
CTCAE grade 2, still persisting for 3 days or more and intolerable after pharmacotherapy</td></tr>
<tr><td>● Proteinuria;
CTCAE grade 2 (≥2.0g/24-hour quantification)</td></tr>
<tr><td>● Liver injury;
total bilirubin >2×ULN and /or aminotransferase >3×ULN in patients with normal baseline;</td></tr>
<tr><td>● Diarrhoea;
CTCAE grade 3, still persisting for 3 days or more after proper antidiarrheal and symptomatic treatment</td></tr>
<tr><td>● Any drug-related AE≥TCTAE grade 3</td></tr>
</table>

5.9.4 Management of Overdose

Symptomatic and supportive treatment should be given in case of overdose (defined as more than 1 dose administrated within 24 hours).

If the dose of study drug exceeds the dose specified in the study protocol, and no relevant symptoms or signs occur, it is not necessary to record it in the case report form.However, the overdose itself must be recorded in the Overdose Report Form of Clinical Studies. For overdose accompanied by a serious adverse event, the SAE diagnosis/symptoms must be recorded in the Serious Adverse Event Form. If there is overdose with adverse event, and the adverse event is serious, this adverse event should be recorded in the Adverse Event Form.In addition, the overdose must also be recorded in the Overdose Report Form of Clinical Studies.

5.10 Concomitant therapy

Subjects cannot simultaneously receive other antitumor therapy at the entry in the study and throughout the study, including cytotoxic agent (except non-antitumor chemotherapy), radiotherapy, biological therapy, endocrine therapy, or any other study drugs, and traditional Chinese medicine with antitumor activity cannot be used either during the study.Systemic antitumor therapy or therapy with other study drugs must be ended 4 weeks prior to the enrollment in this study.

If it is necessary for subjects to receive radiotherapy for tumor during the study, the Fruquintinib therapy will be discontinued.If local radiotherapy is indicated for the subject (eg., local radiotherapy for relieving carcinous osteodynia), the investigator should sufficiently evaluate the possibility of tumor progression, the subject could resume the study drug 7 days after the end of local radiotherapy but the following conditions need to be met:

- Recovery from radiotherapy related and < NCI CTC-AE grade 2 toxicity;
- No progression of tumor.

5.11 Other concomitant treatments

Investigators need to follow the following guidelines and use concomitant medications carefully during the study, as to ensure the subject's safety to the maximum extent.

- The best supportive therapy is allowed during the study, but preventive use of antiemetic drug is not allowed. Other drugs that can trigger or exacerbate the clinical symptoms cannot be used concomitantly, and should be intensively monitored when their use is essential.
- It was demonstrated in the preclinical study that Fruquintinib was metabolized via hepatic cytochrome P450 3A4. The potent inducers of CYP3A4 enzyme, such as phenytoin, phenobarbital and rifampicin (not limiting to the aforementioned drugs) and the potent inhibitors of CYP3A4 enzyme, including ketoconazole, itraconazole, fluconazole, indinavir, erythromycin (not limiting to the aforementioned drugs) could significantly affect the metabolism of Fruquintinib in humans. Cautions should be exercised in enrollment of the subjects concomitantly given known CYP3A4 inducers and CYP3A4 inhibitors. If such drugs are administered concomitantly in the study, cautions need to be exercised in its use, and exposure and adverse reactions should be closely monitored.
- Subjects are allowed to take anticoagulant (e.g., warfarin), but relevant coagulation parameters, such as INR, need to be monitored; if it is required for treatment, low molecular heparin sodium can be used.
- The investigator decides the use of other concomitant medications, as to ensure the subject's benefit and safety.

All the drugs including the study drug must be recorded in the case report form.

5.12 Treatment compliance

The investigators should record the amount of the study drug dispensed and recovered with the date and the actual dose administered promptly and accurately for each subject, the actual dose administered should be in line with the dose required by the protocol. The compliance with the drug will be judged according to the drug counting dispensed, returned and lost in each cycle of treatment and at withdrawal from the study.

The drug inventory list should be inspected by the sponsor or its assigned monitors at any time.Patients are requested to return all the used or unused bottles of Fruquintinib to the study site, as to evaluate their compliance. At the end of study, all the materials provided, including partially used or empty drug bottle, must be returned to the study monitor.

6 Variables, evaluation plan and steps

Attached tables (Evaluation form)

Attached Table 1

Study Flowchart

Date / Item	Screening	Treatment period									¹Û²ìÆÚ	Follow-up period
		Cycle 1				Cycle 2		Cycle 3		Cycle 3+		
	-14 D 743D	Day 7	Day 14	Day 21	Day 28 (+1 day)	Day 42 (+2 days)	Day 56 (+2 days)	Day 70 (+3 days)	Day 84 (+3 days)	Follow-up every 4 weeks (+3 days)	Within 30 day post-discontinuation	Telephone follow-up every 3 months(+7 days)
Fruquintinib treatment[8] and PK collection	Group A	Xa(4mg，qd, daily)				Xa		Xa		Xa		
	Group B	Xb(5mg，qd 3-week on/1-week off)				X[b]		X[b]		X[b]		
Expansion part		X[b]								X[b]		
Signing Informed Consent	X											
Randomization	X											
Tumor assessment[1]	X						X			X		
Inclusion/exclusion criteria	X	X										
Previous disease/history of surgery	X											
Concomitant medication details	X	X	X	X	X	X	X	X	X	X	X	
ECOG score/ weight	X	X	X	X	X	X	X	X	X	X	X	
Vital signs[2]	X	X	X	X	X	X	X	X	X	X	X	
Complete physical examination	X	X	X	X	X	X	X	X	X	X	X	

Date / Item	Screening	Treatment period									¹Û²ìÆÚ	Follow-up period
		Cycle 1				Cycle 2		Cycle 3		Cycle 3+		
	-14 D 743D	Day 7	Day 14	Day 21	Day 28 (±1 day)	Day 42 (±2 days)	Day 56 (±2 days)	Day 70 (±3 days)	Day 84 (±3 days)	Follow-up every 4 weeks (±3 days)	Within 30 day post-discontinuation	Telephone follow-up every 3 months(±7 days)
Hematological examination[3]	X	X	X	X	X	X	X	X	X	X	X	
Clinical biochemistry[4]	X	X	X	X	X	X	X	X	X	X	X	
Urinalysis and 24-hour urine protein quantification[5]	X	X	X	X	X	X	X	X	X	X	X	
Fecal occult blood test	X	X	X	X	X	X	X	X	X	X	X	
12-lead ECG[6]	X	X	X	X	X	X	X	X	X	X	X	
Echocardiography	X				X							
Thyroid function test	X				X		X		X	X	X	
Carcinoembryonic antigen (CEA)	X						X			X		
Pregnancy test (as required)[7]	X				X		X		X	X		
Admission to and discharge from hospital		X										
Collecting AEs	X	X	X	X	X	X	X	X	X	X	X	X9
Information on subsequent treatment												X
Survival:											X	X

Notes:

1. The baseline tumor assessment will be done within 2-3 weeks prior to administration, four sites (neck, chest, abdomen and pelvis) are evaluated in accordance with

RECIST1.1 criteria. Using the radiological (CT/MRI) examination, the baseline evaluation and post-treatment efficacy evaluation are performed with the same method and by the same investigator; tumor radiological assessment (CT or MRI) is performed every two cycles of treatment (8 weeks), until progression of disease. If the tumor is assessed as complete response or partial response (CR or PR), it needs to be re-confirmed 4 weeks later. If the subject receives the drug for less than 56 days, tumor assessment could be performed at the end of medication when necessary, according to investigator's judgement.

2. The vital signs include blood pressure, heart rate, respiratory frequency and temperature, and are monitored 2 hours(±1 hour) after administration;
3. Hematological examination: red blood cell, hemoglobin, platelet count, neutrophil and its differential categories; prothrombin time (PT), activated partial thromboplastin time(APTT), thrombin time(TT), fibrinogen quantification(FIB) and INR. If the neutrophil is ≤1×109/L or platelet count is ≤25×109/L, the frequency of repeated testing needs to be increased (once/2-3 days);
4. Serum chemistry: if 3 times elevation of ALT or AST occur during the trial, the frequency of repeated testing needs to be increased (2-3 times/week advised); the creatinine clearance at baseline is calculated based on the creatinine level (formula: Ccr=(140-age)×body weight(kg)/[72×Scr(mg/dl)] or Ccr=[(140-age)×body weight(kg)]/[0.818×Scr(umol/L)], the unit of creatinine should be noted during the calculation of creatinine clearance, the result for female is the calculated result×0.85);
5. Routine urinalysis is used for measurement of urine protein, and needs to be repeated when urine protein ++ occurs once, 24-hour urine protein quantification is required for urine protein++ in consecutive two measurements;
6. 12-lead ECG is performed in screening period, 4 hours(±1 hour) after administration on Day 7, 14, 21 and 28; and by the frequency of follow-up from the 2nd cycle of therapy;
7. Pregnancy test is performed in screening period, prior to administration, after administration for 28 days and after the end of each cycle of treatment;
8. The detailed administration method of Fruquintinib is provided in Section 5.1 in the protocol.
9. Adverse event is collected to 30 days after the last dose, and adverse event or abnormal laboratory findings that could not be recovered or explained need to be collected until they are recovered or explainable.

6.1 Screening examination and eligibility screening form

All the patients must be able to provide the written informed consent form prior to any specific study evaluation and procedure.

Investigators need to complete the eligibility screening form, where the information on screening of all the patients in accordance with the enrollment criteria and subsequent inclusion in or exclusion from the study are recorded.

6.2 Clinical Assessment

6.2.1 Effectiveness

6.2.1.1 Effectiveness endpoint

All the effectiveness endpoints in the randomized comparison study are secondary endpoints.

Secondary endpoints:

Objective response rate (ORR) determined in accordance with RECIST1.1

Disease control rate (CR+PR+SD), where SD ≥8 weeks

Duration of response

Progression-free survival (PFS)

Overall survival (OS)

Primary effectiveness endpoint in the expansion part is the progression-free survival at Week 16

The secondary endpoints are DCR, ORR and OS

6.2.1.2 Assessment of effectiveness

The tumor is evaluated in accordance with RECIST1.1 criteria, and the detailed evaluation criteria are provided in Appendix 2.The radiological evaluation of tumors could use CT or MRI as decided by investigators, however, the apparatus and technical parameters should keep consistent throughout the study; contrast agent should be used in case of no contraindications.If the tumor evaluation has been performed 14-21 days before the first dose, and the same method and apparatus are used in the same hospital, this evaluation could be used as the baseline tumor evaluation.The baseline tumor evaluation should include neck, chest, abdomen, pelvis and any other sites that are suspected to have tumor lesions.Bone scan should be used for follow-up of the disease in patients with bone metastasis.

Record of target lesions: number, site and description of lesions, the maximum diameter measured for each lesion (except lymph node) and the minimum diameter measured for lymph node, sum of diameters including all the target lesions;

Subjects will receive the first tumor evaluation after 2 cycles of treatment, and one evaluation every two cycles subsequently, until progression of disease.If complete response or partial response occurs, it needs to be confirmed after 4 weeks.If the patient interrupts the dose for adverse drug reaction or other reasons, the tumor evaluation will be performed once every 8 weeks as originally scheduled.If the patient discontinues the study drug within 56 days, the tumor evaluation should be performed as early as possible after discontinuation of the drug.

6.2.2 Performance status

For ECOG PS score, evaluation of ECOG PS is advised to be performed by the same investigator throughout the study.See Appendix 1 for the details.

6.2.3 Clinical safety evaluation

6.2.3.1 Safety endpoints

The safety in both of the groups is comprehensively evaluated through the severity and incidence of adverse events, and graded in accordance with NCI CTCAE Version 4.0. Safety endpoints include:

Overall incidence of adverse events

Incidence of grade 3/4 adverse events, incidence of serious adverse events

Incidence of adverse events leading to permanent discontinuation and dose interruption/dose reduction

6.2.3.2 Evaluation of adverse events and safety laboratory parameters

Throughout the study, the clinical safety of the investigational therapy should be evaluated in accordance with NCI-CTC-AE V4.0.

Occurrence of adverse event must be evaluated at each clinical visit.

Complete history needs to be recorded at screening (including demographic profile; collection of history; symptoms recorded such as decreased appetite, asthenia, cough, dyspnea, hemoptysis, pain; record concurrent disease and concomitant medication, previous history of surgery; histologically or cytologically confirmed pathological diagnosis; as well as ECOG physical performance score)

Physical examination needs to be performed at baseline and each visit, or more frequently according to clinical indications.

12-lead ECG is performed at baseline, and whenever clinically indicated in the study.

The clinical serum chemistry, blood and urine tests are performed at baseline, after treatment and during follow-up period according to the study plan.

The detailed evaluation items include:

- ✓ Hematological examination: red blood cell, hemoglobin, platelet count, neutrophil and differential leukocyte count; thrombin time(TT), prothrombin time(PT), activated partial thromboplastin time(APTT), fibrinogen(FIB) and INR.
- ✓ Routine urinalysis: pH, specific gravity, protein, cast, red blood cell, glucose and urine acetone bodies
- ✓ Fecal occult blood test
- ✓ Clinical biochemistry: total protein (TP), albumin (ALB), globulin (G), A/G, glucose, urea nitrogen, creatinine, alkaline phosphatase (ALP), lactate dehydrogenase (LDH), total bilirubin, glutamic-oxaloacetic transaminase (AST), glutamic-pyruvic transaminase (ALT), calcium, phosphrous, magnesium, potassium, sodium, chlorine, amylase, uric acid
- ✓ Routine thyroid function test: at least free triiodothyronine(FT3), free thyroxine(FT4) and TSH
- ✓ 12-lead ECG: P-R interval, QRS interval, Q-T interval, QTc and diagnosis
- ✓ Echocardiography: in particular pay attention to left ventricular ejection function
- ✓ Tumor markers: carcinoembryonic antigen(CEA)

6.2.4 Measurement of drug concentration and pharmacokinetic study

Determination of the drug and its metabolites (metabolite HM5013199 and HM5012569) in the blood sample is performed by Covance pharmaceutical R&D (Shanghai) Co., Ltd., the specific determination method for the plasma drug concentration is established by the testing unit under the assistance from Hutchison Medi Pharma (Shanghai) Co., Ltd., the method and course of experiment are described in detail in the clinical study report. The Drug Metabolism and Kinetics from Hutchison Medi Pharma (Shanghai) Co., Ltd. is responsible for the calculation and analysis of the PK parameters.

Evaluation of the pharmacokinetic profile is performed for all the subjects in the randomized study, the blood collection schedule for the pharmacokinetic (PK) study during the administration is provided in Table 8 and 9.

2.5ml blood is collected each time, a total of about 50ml blood is estimated to be required for PL evaluation.

The sampling time and actual time should be well recorded on the PK sampling page of eCRF/CRF. Any important information related with sampling or blood collection (eg., loss of blood sample, delayed sampling, hemolysis) should also be recorded in the column of note.

The guidelines on PK sample processing and transportation are provided in Appendix 5.

The basic schedule for blood sampling in Group A (4mg qd daily) is provided in Table 8. If there is no change in the dose in the full-range of PK study (in the first 3 cycles of therapy), only the blood samples on D1/2 and D21/22 need to be collected for PK analysis.If the dose

is changed (dose interruption or dose reduction), the PK sampling should comply with the principle of ensuring collection of the new steady state after dose change and resumption of administration. Details are listed below:

1) All the PK sampling days must be the follow-up days scheduled in the protocol.

2) If the subject interrupts or reduces the dose for adverse reaction 22 days after administration, the subject is hospitalized on the first follow-up day (eg., one of Day 42, 56, 70 or 84) after administration for ≥14 days (reaching the steady state) after dose reduction, and the blood samples at 0, 1, 2, 4, 8 and 24 hours after administration on that day (until before administration on the following day) are collected.

3) If the dose is changed prior to Day 21, the originally scheduled plan for blood collection on Day 21 is given up. The remaining blood collection plan refers to the above Item 2), i.e., the subject is hospitalized on the first follow-up day (eg., one of Day 28, 42, 56, 70 or 84) after administration for ≥14 days (reaching the steady state) after resumption of the administration, and the blood samples at 0, 1, 2, 4, 8 and 24 hours after administration on that day (until before administration on the following day) are collected. Blood samples from only 3 intensive time periods are collected at most for each patient.

Table 8. Blood collection schedule in Group A (4mg qd daily):

Pharmacokinetic Sampling Times						
**Time**	10 minutes before administration	**Administration** (in the morning)	1 h	2 h	4 h	8 h
Day 1	√		√	√	√	√
Day 2	√					
D21#	√		√	√	√	√
D21#	√					
After the dose is adjusted to 3mg or 2mg, the blood samples at 0,1,2,4,8 and 24 hours on the first follow-up day (any one of D28,D42,D56,D70 or D84) are collected after consecutive administration for 14 days (reaching the steady state)						
D28/D42/D56/D70/D84*	√		√	√	√	√
D29/D43/D57/D71/D85*	√					

Note：√ represents need of blood collection;
* represents blood collection on that day only when required. Only one of the 5 time periods, i.e., D28/29, D42/43, D56/57, D70/71 and D84/85 is selected for blood collection;
# represents if the dose is changed 21 days ago, the plan for blood collection on D21/22 is given up. The subsequent blood collection day is one day marked *, the principle on decision is provided in the detailed rules above.

The basic schedule for blood collection in group B (5mg qd 3-weeks on/1-week off) is provided in Table 9. If there is no change in the dose in the full-range of PK study (in the

first 3 cycles of therapy), only the blood samples on D1/2 and D21/22 need to be collected for PK analysis. If the dose is changed (dose reduction), the PK sampling should comply with the principle of ensuring collection of the new steady state after dose change and resumption of administration. Details are listed below:

1) If the dose is changed, the administration period needs to be re-calculated; according to the clinical study protocol, the patient needs to return to the hospital for follow-up on Day 14 in the new administration period.

2) If the subject reduces the dose for adverse reaction 22 days or more after administration, the subject is hospitalized on Day 14 after consecutive administration (reaching the steady state) after dose reduction, and the blood samples at 0, 1, 2, 4, 8 and 24h after administration on that day (before administration on the following day) are collected.

3) If the dose is changed prior to Day 21, the originally scheduled plan for blood collection on Day 21 is given up. The remaining blood collection plan refers to the above Item 2), i.e., the subject is hospitalized on follow-up on Day 14 after resumption of the administration, and the blood samples at 0, 1, 2, 4, 8 and 24 hours after administration on that day (until before administration on the following day) are collected. Blood samples from only 3 intensive time periods are collected at most for each patient.

Table 9. Blood collection schedule in group B (5mg qd 3-week on/1-week off):

Pharmacokinetic Sampling Time						
**Time**	10 minutes before administration	administration(in the morning)	1 h	2 h	4 h	8 h
Day 1	√		√	√	√	√
Day 2	√					
D21#	√		√	√	√	√
D21#	√					
After the dose is adjusted to 4mg 3-week on/1-week off or 3mg 3-week on/1-week off, the blood samples at 0, 1, 2, 4, 8 and 24 hours on Day 14 of consecutive administration after dose reduction are collected.						
Day 14 of consecutive administration after dose reduction*	√		√	√	√	√
Day 15 of consecutive administration after dose reduction *	√					

Note：√ represents need of blood collection;
* represents blood collection on that day only when required.
# represents if the dose is changed 21 days ago, the plan for blood collection on D21/22 is given up. The subsequent blood collection day is one day marked *, the principle on decision is provided in the detailed rules above.

6.2.5 Subsequent antitumor therapy

Information on subsequent antitumor therapy needs to be collected and recorded on the eCRF/CRF for patients with progression of disease, including name, dose of the drug and duration of administration. Any information on the radiotherapy for progression of disease will also be collected, including the site of radiotherapy, Gy dose, start and end time.

7 Safety evaluation

7.1 Safety parameters and definition

7.1.1 Adverse event

Adverse event (AE) is defined as any adverse medical event which occurs during administration of study drug but not necessarily correlates with the therapy. Therefore, AE can be any adverse and unwanted signs (including abnormal laboratory findings), symptoms or diseases, which has temporary correlation with the medication but is not necessarily correlated with the medication.

In addition, AE also includes the complications caused by the intervention specified in the protocol, for example, invasive procedure including biopsy; and exacerbation of previous disease during the reporting period of AE, as considered by investigators (except tumor progression).

7.1.2 Serious adverse event

Serious adverse event (SAE) is defined as the adverse event meeting at least one of the following criteria:

—**death:** the adverse event leads to the subject’s death, except that resulted from progression of disease;

—**life-threatening:** the investigator considers the subject is at the direct risk of death when experiencing the adverse event, rather than that it might lead to death on the premise that the event becomes more serious;

—**hospitalization or prolonged length of hospital stay:** the adverse event leads to hospitalization (not including seeing a doctor in emergency room or outpatient department), or occurs during the hospitalization and prolongs the length of hospital stay;

—**permanent or significant disability or loss of function:** the adverse event leads to the substantial impairment in the subject’s activities of daily living (not including relatively minor events in terms of medical significance for loss of function, such as headache, nausea, vomiting, diarrhea, influenza and accidental trauma (eg., ankle sprain));

—**congenital deformity or birth defect:** the neonate (fetus) that is born or aborted by the female subject who has been exposed to the drug or the female partner of the male subject who has been exposed to the drug has congenital deformity or birth defect;

—**significant medical event:** the significant medical event might not cause threat to life, lead to death or hospitalization immediately, but would jeopardize the subject or require medical or surgical treatment to prevent occurrence of any of the above results (subject's death, life-threatening, hospitalization, prolonged length of hospital stay, permanent or significant disability or loss of function, congenital deformity).

7.1.3 Protocol-defined special events

The sponsor will keep persistent and close monitoring of the drug induced hepatic impairment although it is rare.

The serum chemistry should be closely monitored when the following conditions occur

- For subjects with normal transaminase at baseline who have more than 3 times elevation in ALT or AST of normal, or those with elevated transaminase at baseline who are found to have more than two times elevation in ALT or AST of baseline, serum chemistry should be closely monitored (ALT, AST, ALP and TBL) and the frequency of monitoring should be increased (2-3 times/week);
- For subjects with early symptoms of hepatic impairment before they are found having abnormal serum chemistry (eg., decreased appetite, nausea, vomiting, right upper abdominal discomfort, asthenia), serum chemistry test should be performed immediately, and the frequency of monitoring must be increased when the above abnormalities occur.

The following events are the protocol-defined special events:

- Subjects with normal hepatic function at baseline (ALT, AST and bilirubin within normal range) have AST and/or ALT elevated $>3\times$ULN combined with serum total bilirubin elevated $>2\times$ULN in the same blood sample collected.
- Subjects with elevated transaminase at baseline have AST and/or ALT elevated >2 times of baseline value combined with serum total bilirubin elevated $>2\times$ULN in the same blood sample collected.

7.1.4 Progression of disease

In accordance with RECIST criteria 1.1, if the event is confirmed to be consistent with the expected progression pattern of the primary tumor, it should not be considered as AE.Hospitalization resulted only from progression of the disease should not be considered as SAE either.When the symptom could not be completely confirmed to be induced by the progression of disease, or is not consistent with the expected progression pattern of tumor, the relevant clinical symptom could be judged as AE.

The tumor related symptoms could be considered as AE only in the following conditions:

1. Newly occurring (ie., not present at baseline) and no clear correlation with the primary tumor and metastastic lesion, and/or

2. The investigator considers the exacerbation of tumor related symptom is directly correlated with the study drug.

When the event could not be determined to be induced only by the progression of disease, it should be considered as AE, and be reported as when SAE criteria are met.

7.2 Collection and evaluation of safety parameters

7.2.1 Definition of adverse event reporting period

Time Period	Reporting requirements
from the signature of informed consent to before administration of the first dose of study drug	Report SAE caused by the protocol-defined interventions (eg., invasive procedure including the biopsy, drug elution, no treatment preparation period)
from the first dose to within 30 days after the last dose of study drug	Report all the AE, including SAE except progression of disease, and all the death.
post-treatment period (30 days after discontinuation of the study drug)	Only report the SAE related with the study drug.<br>Only report the death as SAE when it is considered to be related with the study drug.

7.2.2 Intensity assessment of adverse events

The intensity of all the AEs is graded in accordance with NCI criteria on common terminology for adverse event (CTCAE) version 4.0 and judged in 5 grades (Grade 1-5).The AE not listed in NCI CTCAE is judged via the following table.

Table 10. Criteria on judgment of the intensity of adverse event

CTC grade	Equal to	Definitions
1	Mild	discomfort observed but no impact on normal daily activities
2	Moderate	the discomfort adequate to reduce or affect daily activities: no use of treatment or medical intervention although they could improve patient's quality of life or relieve symptoms
3	Serious	inability to work or complete daily activities, treatment or medical intervention used to improve patient's quality of life or relieve symptom, delayed treatment would not bring patient in direct threat to life.
4	Life-threatening or disable	direct threat to life or resulting in permanent mental or physical impairment, inability to work or perform daily activities, treatment or medical intervention must be performed to maintain life.
5	Death	AE leading to death

Note: the severity and intensity of a adverse event are different.The intensity is defined as intense degree of adverse event (eg., mild, moderate or severe headache), however, the event itself might be of only minor clinical significance (e.g, severe headache), and could not be judged as SAE, unless it meets the criteria on SAE.Therefore, when reporting AE/SAE, the severity and intensity should be evaluated independently.

7.2.3 Drug-event relationship

The relationship between the study drug and adverse event, or its effect in the adverse

event could be judged using the following category and criteria:

1 unrelated: the adverse event is determined to be caused by other reasons (disease, environment, etc.) after careful medical judgment;

2 possibly unrelated: the adverse event is considered to be possibly unrelated with the study drug after careful medical judgment;

- no temporal relationship between the occurrence of adverse event and use of the drug
- The adverse event might be caused by other factors, including change in the course of disease, environment, or administration of other drugs.
- The adverse event is unrelated with the known properties of the drug
- No more occurrence or exacerbation of the adverse event after resumption of the administration

3 possibly related: (must have the first 2 items) the adverse event is considered to be possibly related with the study drug after careful medical judgment:

- temporal relationship between the occurrence of adverse event and use of the drug
- The occurrence of adverse event could not be excluded to be related with the course of disease, environment, toxicity or administration of other drugs.
- The adverse event is consistent with the known properties of the drug.

4 Related: (must have the first 3 items) the adverse event is considered to be related with the study drug after careful medical judgment:

- strong temporal relationship between the occurrence of adverse event and use of the drug
- It could not be explained with the reasons of course of disease, environment, toxicity and administration of other drugs.
- The adverse event is resolved or relieved after discontinuation of the drug or dose reduction, and recurred after resumption.
- The adverse event is consistent with the known properties of the drug.

When reporting SAE, if the investigator judges it is possibly related, possibly unrelated or unrelated, the alternate reasons causing the SAE must be provided by the investigator.

7.3 Recording and reporting of safety parameters

7.3.1 Recording of adverse events.

During the reporting period of protocol-defined adverse events, the investigator is responsible for collecting all the AEs (including SAE) and recording them in the

CRF/eCRF.When recording the AE, the investigator should use proper and standard medical terms, and avoid spoken language and abbreviations.

7.3.2 Expedited reporting of serious adverse events

During the study, any serious adverse event regardless of the correlation with the study drug, serious adverse events judged by the principal investigator or assistant investigator, or the serious adverse events meeting the above criteria should be reported.The investigator should fax the completed serious adverse event report form to the food and drug administration in relevant province, autonomous regions and municipalities directly under the central government, China Food and Drug Administration, the sponsor and ethics committee within 24 hours after awareness of the serious adverse event.The clinical monitor shall review the written report within 5 business days after receiving it. The report should include demographics, history of medication, adverse events and concomitant medication.The investigator should record all the known information related with the event in the first report.The investigator should complete the follow-up report in the same time limit after acquisition of the follow-up information. If a non-serious AE progresses to serious, this and other relevant follow-up information must be reported within 24 hours as described above.Regardless of the correlation with the study drug, all the serious adverse events should be managed appropriately, until the investigator juges the patient has been recovered from the event or the event turns to be chronic or stable disease.

In order for the sponsor to obtain the safety data in a timely and comprehensive manner, the investigator should fill in the SAE report form in clinical study provided by Hutchison Medi Pharma (Shanghai) Co., Ltd., and fax it to the company within 24 hours, in addition to the serious adverse event report form from CFDA, for all the serious adverse events. If the subject is hospitalized for serious adverse event, the diagnostic and therapeutic information provided by the hospital should be faxed to Hutchison Medi Pharma (Shanghai) Co., Ltd. as soon as possible.

- China Food and Drug Administration Fax:**010-68586295**
- Hutchison Medi Pharma (Shanghai) Co., Ltd. Fax: **021-50793900**

7.3.3 Reporting of protocol-defined special events

The protocol-defined special events, regardless of SAE or the correlation with the study drug, need to be reported to Hutchison Medi Pharma (Shanghai) Co., Ltd.

- those meeting SAE criteria need to be reported in accordance with the expedited reporting procedure for serious adverse events (as shown in 7.3.2);
- those not meeting SAE criteria only need to complete the SAE report form in clinical study provided by Hutchison Medi Pharma (Shanghai) Co., Ltd. (select “protocol-defined special event” in the column of severity criteria), and fax it to the sponsor within 24 hours.

7.3.4 Recording and reporting of abnormal laboratory examinations

All the results from laboatory examinations could be recorded on the laboratory examination page in CRF/eCRF.

Any treatment-emergent sudden abnormal laboratory result should be recorded as one diagnosis on the AE page of CRF/eCRF when it is clinically significant and for example, meets at least one of the following conditions:

- ♦ with clinical symptoms;
- ♦ leading to change of study drug (eg., dose adjustment, interruption or permanent discontinuation);
- ♦ requiring to change the concomitant therapy (eg., concomitant medication, addition of treatment or management, interruption, discontinuation or other changes)

This is applicable for the results of laboratory examinations for safety and efficacy described in or out of any protocol after the first dose, if they are out of the reference range and meet the criteria on clinical significance.

This is not applicable for the results of laboratory examinations that are out of the reference range but don’t meet the criteria on clinical significance (only analyzed and reported as laboratory abnormality).

Please note: any abnormal laboratory result should be reported as SAE if it meets the criteria on SAE, and recorded as AE in the CRF/eCRF.

The procedure for all the AE/SAE reporting is provided in Appendix 3.

7.4 Follow-up of subject

7.4.1 Follow-up of Adverse Event(s)

The investigator should follow up all the AEs, until occurrence of any of the following conditions:

- AE is relieved or improved to baseline level;
- No further improvement is expected, as confirmed by the investigator;
- Subject dies;
- loss of contact with the subject;
- no correlation between the AE and investigational therapy, as confirmed by the investigator;
- start of new anticancer therapy;
- No more collection of clinical or safetydata, or final closure of database.

The final outcome of each AE (including the date when AE is relievd or the subject dies) must be recorded in the CRF/eCRF.

7.4.2 Follow-up of Serious Adverse Event(s)

All the SAEs should be followed up as much as possible, until they are relieved or improved to baseline level, subject’s death, loss of contact or the investigator finally confirms the SAE is unrelated with the investigational therapy.The investigator should fill the follow-up information of SAE as the follow-up report or summary report in the Serious Adverse Event Report Form, meanwhile fill in the SAE Report Form of Clinical Studies provided by the sponsor, and report it in accordance with the expedited reporting procedure for serious adverse event (as shown in 7.3.2).

The sponsor could obtain more information about the event from the investigators via telephone, email, fax or monitoring visit for part of the SAEs.

7.4.3 Follow-up of protocol-defined special events

In addition to the routine follow-up of SAE, all the protocol-defined special events need to be followed up in accordance with the Clinical Assessment of Hepatic Impairment provided in Appendix 4 of the clinical study protocol, as to obtain more valid information for evaluation of this event.

7.4.4 Follow-up of abnormal laboratory results

The test should be performed again and followed up for any abnormal laboratory results with medical significance but unclear explanation until it is recovered to normal, baseline and/or adequate explanation is found.The clear explanation should be recorded on the CRF/eCRF if it is available.

7.5 Pregnancy and management of pregnancy

Female subjects must be informed that any pregnancy during the study must be reported to the investigator immediately, and the study drug must be discontinued.Any pregnancy within 30 days after the end of therapy should also be reported to the investigators.

The pregnancy itself is not an adverse event, unless it could affect and interfere with the efficacy of contraceptive agent.However, once pregnancy occurs, the investigator must report it to the sponsor within 24 hours after awareness of the event.At the same time, the investigator should sincerely advise the subject and discuss with her the risk of continuous pregnancy as well as the potential effect on the fetus.The pregnancy in the female partner of male subject during the study needs to be reported as well.In summary, the partner of a subject should be also advised sincerely and followed up.

The event of pregnancy should be followed up until the end of pregnancy.Outcome of all the pregnancies (spontaneous abortion, artificial termination of pregnancy, normal birth or congenital abnormality) must be followed up and recorded, even though the subject

has withdrawn from the study.Outcome of all the pregnancies must be reported through pregnancy outcome form.

All congenital abnormalities and birth defects are SAEs.Abortion should be reported as SAE regardless of therapeutic or spontaneous abortion (important medical event).

8 Statistics and analysis plan

8.1 Primary and secondary research variables

Primary variables:

Overall incidence of adverse events

Incidence of Grade 3/4 adverse events

Incidence of serious adverse events

Incidence of adverse events leading to permanent discontinuation

Incidence of adverse events leading to interruption/reduced dose

16-week progression-free survival rate

Secondary variable:

Effectiveness:

"Event occurrence date" variable: Calculated since the day when the informed consent is signed.If no event is observed, there is no date of end, and a so called cutoff observation has to be considered for analysis.The cutoff date is: The last date, such as for "last tumor measurement", "last medication registration", or "last visit".

Secondary efficacy variables include objective response rate, disease control rate, duration of response, progression-free survival, and overall survival.

Objective response rate (ORR): defined as the incidence of confirmed complete response (CR) or partial response (PR), the target lesions and non-target lesions are evaluated with validated radiological method and judged in accordance with RECIST criteria 1.1.

Progression-free survival (PFS): defined as the time from the signature of informed consent form to the first recording of progression of disease (PD) or date of death, whichever comes first. If no PD is observed, the censoring date should be the date of the last tumor measurement.

Overall survival (OS): defined as the time from the signature of informed consent form to date of death for any reason. If no death is observed, the last follow-up date is regarded as the censoring date.

Disease control rate (DCR): defined as the incidence of confirmed complete response (CR), partial response (PR) and stable disease (SD), the target lesions and non-target lesions are

evaluated with validated radiological method and judged in accordance with RECIST criteria 1.1.

Duration of response: defined as the time from the first recording of CR or PR to the first finding of PD for patients who have achieved response. For those with no record of PD after CR or PR, the censoring date is the date of the last tumor measurement.

Safety and Tolerability:

Sfety and tolerability of the treatment are evaluated by incidence and severity of adverse events, laboratory tests, vital signs, ECG, LVEF, and ECOG performance status.

8.2 Statistical and analytical methods

8.2.1 Data Set Analyzed

Intent-to-treat analysis set (ITT set): According to the intent-to-treat analysis principle, ITT set includes all randomized patients, and is analyzed by the groups into which the patients are assigned for randomization,whether they receive the investigational treatment or not.

Safety Analysis Set (SAS set): The SAS set includes all randomized patients who receive at least 1 dose of the investigational drug.

8.2.2 Statistical Methods

Efficacy Analysis:

Randomized comparison part-secondary efficacy endpoint analysis:

The inter-group difference of objective response rate is compared by CMH Chi-square test.

Secondary efficacy endpoint analysis: Descriptive statistical analysis.

Progression-free survival is analyzed by Kaplan-Meier method;

Overall survival is analyzed by Kaplan-Meier method;

The inter-group difference of disease control rate is compared by CMH Chi-square test;

The duration of response is analyzed by Kaplan-Meier method; the median time to response and its 95% confidence interval are listed for the two groups.

Expansion part- primary efficacy endpoint analysis:

The 16-week progression-free survival rate is calculated in patients with advanced colorectal cancer who have failed with standard care and are receiving drug administration of 5 mg qd 3-week on/1-week off in the study. Secondary endpoints are analyzed by descriptive statistical analysis.

At the end of the trial, the survival (including median survival and 9-month survival rate) in patients with advanced colorectal cancer who have failed with standard care and are receiving drug administration of 5 mg qd 3-week on/1-week off is analyzed.

Safety analysis:

Safety analysis population: The safety analysis population includes the patients signing the informed consent who receive at least one dose of the treatment.

All safety parameters are summarized, and are tabulated by the safety population.

The frequency table (overall and by intensity) of adverse events is listed by system organ class. In the table of the overall incidence of adverse event, subjects with the same adverse event more than once is only counted once in the frequency table.

Shift table and frequency table are used simultaneously for summary of laboratory data according to sampling timepoint.

All the AEs and abnormal laboratory variables are evaluated according to CTCAE v4.0 grading system.

ECOG performance status will be summarized by descriptive statistics.Vital signs, ECG, and echocardiography will be described by tabulation.

During the randomized comparison part, inter-group differences are compared by CMH Chi-square test, based on the primary safety endpoints.

The overall incidence of adverse events, incidence of G3/4 adverse events, and severe adverse events are compared between the two groups;

The ratios of permament discontinuation due to adverse events, and the ratio of interruption or reduced dose due to adverse events are compared between the two groups.

PK parameters:

WinNonliln enterprise version software is used for noncompartmental model analysis of plasma drug concentration.

Univariate analysis of variance is used for Cmax, $AUC_{0-\infty}$ and AUC_{0-t} during the study using dose group as the fixed factor.

8.2.3 Statistical Software and General Requirements

Data are analyzed by the internationally recognized statistical software (such as SAS 9.2 software (SAS Institute Inc., Cary, NC, USA)), and pharmacokinetic parameters are calculated by WinNonlin software or other recognized softwares.

A complete, detailed statistical analysis is described in the statistical analysis plan, and the statistical analysis plan is finalized prior to locking the database.

8.2.4 Interim Analyses

A interim analysis is performed when at least 30 subjects complete 4 cycles and 40 subjects complete 2 cycles of evaluation in the randomized comparison part of this study as to compare the safety and tolerability of two different administration methods of Fruquintinib. The primary parameters are statistically analyzed and combined with efficacy parameters

for comprehensive evaluation to determine the recommended dose group in phase Ⅱ/Ⅲ study for expansion part.

8.3 Sample size

About 60 subjects are planned to be enrolled.

Here, 40 subjects are enrolled into the randomized comparison part, and approximately 20 more subjects are enrolled into the single-arm expansion part.

Randomized comparison part:

Group A (4 mg, qd, daily) consists of 20 patients;

Group B (5 mg, qd, 3-week on/1-week off) consists of 20 patients;

Expansion part:

Group B (5 mg, qd, 3-week on/1-week off) consists of approximately 20 patients.

9 Data Quality Assurance

Electronic data management system (DASforeCDM5.0) is used for this trial.

Establishment of electronic case report form (eCRF): eCRF is constructed by data administrator according to study protocol and medical record.

Allocation of authorities: the data administrators create the account respectively according to different identities, including investigator, sponsor, monitor and auditor, and are authorized different permissions to access eCDM. For example, investigators at each site can only find the content at their site and have the right to modify the data, the sponsor is limited only to look through all the cases; monitor and auditor can read the cases at each site, insert remarks or questions but have no right to modify the data.

Data entry: clinical investigators or their designated data entry clerk (clinical coordinator) enter the data from the medical records in the eCRF promptly and accurately. eCRF is not used as the original record, its content originates from medical records.

Questions on the data and answers: monitors monitor the data through eCDM and raise questions on line at any time when they find questions, the investigators answer the questions on line and modifiy wrong data, monitors could repeat their questions when necessary.

Data lock and export: the data are locked by the data administrator after each subject complete the trial and all the data are confirmed correct by the monitor, until the lock of the data from the last subject. The data are exported into the designated database by the data administrator after complete data lock, and submitted to statisticians for analysis.

eCRF is printed for archive at the end of study, if required. Data management center will maintain the electronic data until 5 years after its launch in the market. The data management center can open the system at any time after appointment during this period if

SFDA needs to check.

EDC System Emergency Plan

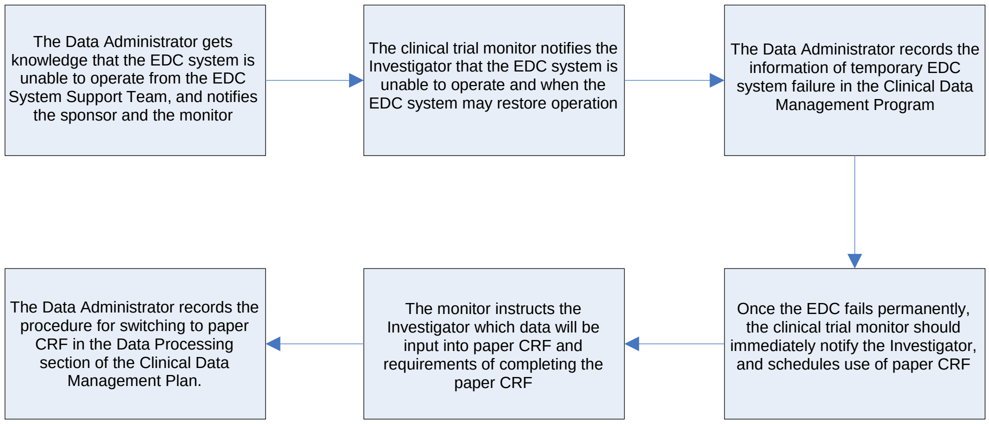

10 Study committee

This study will establish a Data Monitoring Committee (DMC).The Committee consists of and is charged by the medical monitor of the sponsor, safety data monitor, and the principal investigator.

DMC will review safety data.Safety data include: Demographic data, adverse events, severe adverse events, and laboratory abnormal values.

After data review, DMC will provide sugestions whether the study will be continued, and whether it is required to revise the protocol or to discontinue the study.These will be determined eventually by Hutchison Medi Pharma (Shanghai) Co., Ltd.

11 Ethics

11.1 Local Regulations/Declaration of Helsinki

The Investigator will guarantee that the study will be conducted in strict accordance with the principle of the "Declaration of Helsinki" and/or with the laws and regulations of the country where the study is conducted, and provide maximized protection for patients.The study must adhere to the principles in the ICH Guideline for "Good Clinical Practices" (since Jan 1997) or local laws, to provide maximized protection for patients.

11.2 Informed Consent

The responsibilities of the Investigator or the Investigator Designee (if allowed by local laws) are to explain adequately the purpose, method, expected benefits, and potential hazards of the study, and obtain the informed consent from each subject participating in the study.For a patient who is not qualified to or cannot provide a legal informed consent, a written informed consent must be obtained from his/her legal guardianIf a patient and his/her legal guardian cannot read, then the whole discussion course of informed consent must

be witnessed by a notary.After the patient and his/her guardian agree verbally to participate in the study, then the notary signs in the informed consent form, to prove that the information of the informed consent is explained and understood accurately.The Investigator or his/her designee must also explain that, the patient is free to refuse to participate or discontinue the study in any reason at any time.The content of case report form (including some part that are used to record patient's informed consent) of this study must be filled correctly.If a new safety information result in any obvious change of the risk/benefit evaluation, then the informed consent must be reviewed and updated if necessary.All patients (including those being treated) shall be informed of such new information, and provided with a revised informed consent, and continue the study if they give the corresponding informed consent.

11.3 Independent Ethics Committee/Institutional Review Board

The protocol and any relevant materials provided to patients (such as patient's manual or study description), as well as advertisement and compensation given to patients will be submitted to Independent Ethics Committee by the Investigator.Prior to start of the study, it must be approved by the Ethics Committee, and is recorded in the form of Letter to the Investigator, including the dates for conference of the Ethics Committee, and for granting approval.

After receiving the approval document from the Ethics Committee, if any change is made to the protocol, investigator shall also submit it to the Ethics Committee accordance with the procedure and the regulatory requirements.

12 Conditions to Change the Protocol

The protocol and the study flow cannot be changed, without mutual agreement by the Principal Investigator and Hutchison Medi Pharma (Shanghai) Co., Ltd.

If the protocol must be revised, the revised content or the new version of the protocol (revision) must be submitted to the Ethics Committee for review to obtain a written approval.If required, it is also submitted to a local regulatory authority or is approved, and must follow the requirements of the local regulatory authority.

If the revision of the protocol requires to change the informed consent in study site, then Hutchison Medi Pharma (Shanghai) Co., Ltd. and the Ethics Committee of the study site must be notified.A written approval must be obtained from Hutchison Medi Pharma (Shanghai) Co., Ltd. and the Ethics Committee for application of the revised informed consent.

Hutchison Medi Pharma (Shanghai) Co., Ltd. will distribute the protocol revision and the new version to each principal investigator, and then the Principal Investigator will distribute such documents to the corresponding Ethics Committee and other study personnel.

13 Condition to End the Protocol

The sponsor and the Investigator have the right to terminate the study at any time.If it is necessary to terminate the trial, it must be ended through mutual insepection and negotiation.To discontinue the study, Hutchison Medi Pharma (Shanghai) Co., Ltd. and the Investigator will ensure to maximize patients' benefits.

14 Record, Storage, and Access to the Study Documents

14.1 Storage of Investigator's Documents/Records

The Investigator shall agree to keep all research data, including confirmation of all participating subjects (which may check efficiently different recorded data, such as eCRF/CRF and hospital original records), original informed consent forms of all patients, eCRF/CRF, and detailed records for drug dispension, etc.

All data of the clinical trial are property of Hutchison Medi Pharma (Shanghai) Co., Ltd., and shall not be disclosed to any person unrelated with this trial in any form, without agreement by the sponsor.The related data of the study can only be published after agreement by the sponsor.

14.2 Original Documents and Background Information

The original documents may prove existence of patients, as well as integrity of collected data.The original data are archived in the study site of the Investigator.

The data in eCRF/CRF transferred from the original documents must be consistent with the original documents, and any difference must be explained.According to the trial, the Investigator may require previous medical records of transferral records, or may require current medical records.

All data in eCRF/CRF must be originated from original documents.

14.3 Direct Access to Original Data and Documents

The Investigator/Institution will agree to carry out trial-related examination, audits, IRB/IEC review, as well as examination by the regulatory authority, and provide direct access to all relevant original data/documents.eCRF/CRF as well as all source documents, including pathography records, as well as copies of laboratory tests and medical examinations, must be available for examination by the clinical monitor, auditor, and health regulatory authority at any time.Clinical Research Associate (CRA) as well as the auditor may review all eCRF/CRFs as well as written informed consents.

15 Study monitoring

Prior to enrollment of the first subject, the authorized monitor on behalf of Hutchison Medi Pharma (Shanghai) Co., Ltd. shall be at the study site:

- ♦ Ensure the facilities and equipment available;

- ♦ Discuss with the Investigator (or other study personnel) the responsibilities of the research members and monitors in the study, and sign relevant agreements;
- ♦ During the study, the monitor shall visit the study site regularly, and keep in close contact with the Investigator;
- ♦ Provide sufficient information and support to the investigators;
- ♦ Ensure that the research facilities and equipment in place;
- ♦ Ensure that the group follows the protocol, and the data are recorded accurately in eCRF/CRFs, and study drugs are counted;
- ♦ Ensure to complete verification of the data (verify that the data in eCRF/CRFs are consistent with the hospital's medical records, and other records related with the study).It is required that the original records of each subject may be obtained.

If the Investigator requires more information and suggestions, the monitor and other representatives of Hutchison Medi Pharma (Shanghai) Co., Ltd. will visit the study site.

16 Confidentiality of the Trial Documents and Patients' Records

The Investigator must ensure to maintain patients' anonymity and prevent to disclose a patients' identity to any unauthorized party.Patients cannot be identified with a patient's name but only with his/her identification code in CRF/ECRFs or other documents submitted to the sponsor.The Investigator shall record an enrollment registry form of patients, to display a patient' code, name, and address.The Investigator shall store strictly certain documents such as a patient's informed consent, and never submit to Hutchison Medi Pharma (Shanghai) Co., Ltd.

17 Publication of the Data, and Protection of Business Secrets

The results of the study may be possibly published or issued in a scientific conference.If planned, then the Investigator shall agree to submit all subscripts or abstracts to Hutchison Medi Pharma (Shanghai) Co., Ltd. befor submissionIn this way, it may protect the sponsor's proprietary information, and meanwhile the sponsor may give some comments to the Investigator as the Investigator may not know information about other studies.

According to the standard publication and ethical principles, Hutchison Medi Pharma (Shanghai) Co., Ltd. supports generally publication of the data from multiple centers on the whole other than those from a single center.In such case, a coordinating investigator is designated after mutual consent.

18 References

1. National Comprehensive Cancer Network (NCCN) 结直肠癌临床实践指南, Version 2012.

2. Hurwitz H, Fehrenbacher L, Novotny W, et al: Bevacizumab plus irinotecan, fluorouracil, and leucovorin for metastatic colorectal cancer. N Engl J Med 350:2335-2342, 2004
3. Demetri GD, Desai J, Fletcher JA, et al: SU11248, a multi-targeted tyrosine kinase inhibitor, can overcome imatinib (IM) resistance caused by diverse genomic mechanisms in patients (pts) with metastatic gastrointestinal stromal tumor (GIST). J Clin Oncol 22:3001, 2004
4. Axel Grothey, Alberto F. Sobrero, Salvatore Siena,et al: Results of a phase III randomized, double-blind, placebo-controlled, multicenter trial (CORRECT) of regorafenib plus best supportive care (BSC) versus placebo plus BSC in patients (pts) with metastatic colorectal cancer (mCRC) who have progressed after standard therapies. J Clin Oncol 30, 2012 (suppl 4; abstr LBA385)
5. 郝希山。2011 年第十三届中国科协年会分会“癌症流行趋势和防控的策略研讨会”

Appendix 1 ECOG performance status

The patients are graded according to ECOG performance status, and the criteria are described as follows:

grades	ECOG
0	Fully active, able to carry on all pre-disease performance without restriction
1	Restricted in physically strenuous activity but ambulatory and able to carry out work of a light or sedentary nature, e.g., light house work, office work
2	Ambulatory and capable of all selfcare but unable to carry out any work activities. Up and about more than 50% of waking hours
3	Capable of only limited selfcare, confined to bed or chair more than 50% of waking hours
4	Completely disabled. Cannot carry on any selfcare. Totally confined to bed or chair
5	Death

Appendix 2 RECIST version 1.1 for Tumor Evaluation

RECIST version 1.1

(Response Evaluation Criteria in Solid Tumors RECIST Version 1.1)

As RICIST version 1.1 is not published in any formal Chinese version, and this version is an internal translation. Please refer to the English version for more detailed information.

1 Background

1.1 History of the RECIST Criteria

To evaluate any change of tumor burden is an important feature in clinical evaluation of cancer treatment.The times to tumor reduction (objective response) and progression of disease are important judgment endpoints in a clinical trial of cancer.To screen new antitumor drugs, the evidences from studies of many years supports that tumor reduction is used as an endpoint for Phase II trial.Those studies suggest that, for solid tumors, a drug that may promote tumor reduction in some patients may be later proved possibly (imperfect though) to increase a patient's overall survival or allow him/her to have an opportunity to enter event evaluation in a Phase III randomized trial.Presently, among the variables to evaluation treatment effects in Phase II screening trials, objective response is more reliable than any other biomarkers.Moreover, among Phases II and III clinical trials for drug development, the time to progression of disease (or progression-free survival) is gradually used as an endpoint to judge effectiveness in clinical trials in severe disease, and is based on anatomic measurement of tumor size.

However, the two judgment endpoints i.e. objective response and time to progression of disease are valuable only when the widely accepted and easy-to-use criteria and rules are established on the basis of tumor burden anatomy.In 1981, the World Health Organization (WHO) first published the criteria for response in tumors, which have been mainly used in trials in which tumor response is a primary endpoint.The WHO criteria introduced the overall evaluation concept of tumor burden by measuring and totaling the 2-dimensional of tumor, and judged treatment response by evaluating a change during treatment since baseline.However, in a dozen years after publication of the criteria, the Cooperative Group using the criteria and pharmaceutical companies modified them to adapt new technologies or proposed unclear points in the original literatures, leading to confusion in interpreting the results of a trial.In fact, the application of various response criteria resulted in distinct differences of treatment effects by the same therapeutic method.For those questions, an international work group was established in the mid-19th century to standardize and simplify the criteria for response.The new criteria, also known as RECIST (Response Evaluation Criteria in Solid tumors) was published in 2000.The initial key features of RECIST included: Determination of minimum measurable lesions; description of lesions to be followed (10 lesions at maximum; 5 lesions per organ at maximum); use of 1 dimension other than 2 dimensions; and overall evaluation of tumor burden.Later, those criteria have been used widely by academic communities, cooperative groups, and the pharmaceutical

industry. The initial endpoints of the criteria are objective response or progression of disease.Moreover, the competent authority accepts RECIST as suitable criteria for those evaluation.

2. Purpose of the Guideline

The Guideline describes a standard method for measuring solid tumors, and clarifies the objective judgment criteria for change of tumor size, used in clinical trials in adults and pediatric patients.It is expected that those criteria will be used in trials in which objective response is used as the primary study endpoint, as well as in trials using steady-state disease evaluation or tumor progression or analyzing time to progression as variables, as the measurement of all therapeutic effects are based on evaluation of anatomical tumor burden and its change.In this article, the proportion of patients meeting the inclusion criteria are not hypothesized. The patients adopt one trial endpoint that may predict effectiveness of one drug or therapeutic regimen whose definiation is dependent on type of tumor in the ongoing trial and special drugs under investigation.The trial protocol must include an appropriate statistics section, where the sample size of the trial and the effectiveness parameters on which the inclusion criteria are based are defined.This Guideline provides the definition and criteria to judge tumor response, and also proposes suggestions for reporting criteria of the results of a clinical trial using tumor response as the trial endpoint.

Although those guidelines may be used in research of malignant cerebral tumors, the criteria for evaluation of response have been published separately in this field.As the international rules for evaluation of lymphoma response have also be published separately, this Guideline is not used in research of malignant lymphoma.

Finally, many oncologists follow patients' malignant disease by multiple imaging investigations in their daily clinical practices, and determine further treatment regimen on the basis of both objective and symptom criteria.Those RECIST guidelines may play an important role only when the treatment oncologist judge them as rational.

3. Baseline Tumor Measurement

3.1 Definition

At baseline status, tumor lesion/lymph nodes are divided into measurable and immeasurable categories as follows:

3.1.1 Measurable

Neoplastic lesion: It must measure accurately to at least one size of not less than the lower limit (of instrument detection) (the maximum diameter in the measuring instrument will be recorded):

- A 10-mm size is scanned by CT (in a thickness of not less than 5 mm; see Appendix III, Guideline for Imaging).

- For clinical test, 10 mm is measured by a caliper (a lesion that cannot be measured by a caliper is recorded as immeasurable).
- 20 mm is examined by chest X-ray radiography.

Malignant lymph nodes: When evaluated by CT scanning (CT scanning layer thickness is not more than 5 mm as suggested), it is considered as pathological expansion and measurable lesion only when the short axis of lymph nodes must be up to 15 mm.The short axis length is measured and followed in preoperative and subsequent practices.The data for measurement of lymph nodes may be obtained from notes under "Preoperative Documents for Target and Non-target Lesions".

3.1.2 Immeasurable (Tumor)

All the other lesions, including small lesions (the maximum diameter is less than 10 mm, or the short axis of pathological lymph nodes ranges between 10-<15 mm), as well as truly immeasurable lesions.The lesions considered as truly immeasurable lesions include: Pharmacologically confirmed meningeal diseases, ascites, pleural effusion or pericardial effusion, inflammatory breast diseases, lymph vessel-involved skin or pulmonary, abdominal masses/abdominal organ gigantisms, which cannot be measured by imaging reconstruction technique.

3.1.3 Special Considerations for Measurable Lesions

Special attention has to be paid to bone lesions, cystic lesions, or lesions previously treated locally:

Bone lesions:

- Bone scanning, PET scanning, or plain radiography are considered inadequate for measuring bone lesions.However, those techniques may be used to confirm pharmacologically presence or absence of bone lesions.
- If the soft tissue part meets the definition of the above measurable lesions, lytic bone lesion with discernible soft tissues, or mixed acute lytic bone lesion may be evaluated by cross imaging techniques such as CT or MRI, then they are considered as measurable lesions.
- Osteogenic lesions are immeasurable.

Cystic lesions:

- Simple cysts meeting the X-ray definition of the inclusion criteria should not be considered as malignant lesions (they are not measurable and are not immeasurable), as they are simple cysts by the definition.
- Meeting the above definition of measurable lesion, "cystic lesions" characterized by cystic metastasis may be considered as measurable lesions.However, in case of

noncystic lesions present in the body of the same patient, the target lesions are included preferentially.

Lesions previously treated locally:

- The lesions located in the previous irradiation area or at the site receiving other local treatment are usually considered as measurable, unless it has proved that the lesions still continue.The protocol should explain under what conditions those lesions are considered measurable.

3.2. Specification for Measuring Methods

3.2.1. Measurement of Lesions

For clinical evaluation, the lesions are measured by a caliper (vernier caliper), and all measurements are recorded in metric unit.All baseline evaluations must be carried out as close to initiation of treatment as possible, and cannot be as early as 4 weeks.

3.2.2. Measuring Methods

Each reported lesion shall be described by the same judgment method and technique during the baseline and follow-up periods.Generally they are evaluated by imaging measurements other than clinical examination, unless it is found that the lesions are not suitable for imaging measurements during follow-up.

Clinical lesion: Only superficial lesions (such as small subcutaneous nodes) with a diameter of more than 10 mm, as measured by vernier caliper, are considered measurable.For patients with skin lesions, record by color photographs is suggested, in which the proportion for measuring lesion size is annexed.As previously mentioned, when the lesions may be examined clinically or by imaging technique, then it is examined by imaging technique, as imaging evaluation is more objective and may be used for final review of clinical investigation.

Chest X-ray radiography: Between chest CT and chest X-ray radiography, chest CT is selected preferentially, especially when progression of disease is used as an important endpoint variable, as CT scanning is more sensitive in identifying new lesions than X-ray radiography.However, if X-ray radiography visualizes clear border of lesions, surrounded by inflated lung, the lesions are considered measurable. See Appendix II for details.

CT and MRI: Presently, CT is the most efficient and of good repeatability test method used to evaluate lesions.As defined in the Guideline, measurable lesions are based on a layer thickness of not more than 5 mm, if CT scanning is used.As shown in Appendix II, when the CT layer thickness is more than 5 mm, the minimum measurable lesion is 2 times the layer thickness.MRI may also be used in some cases (such as whole body scanning).See Appendix II for more comments on response evaluation in solid tumors by CT and MIR examinations.

Ultrasonography: Ultrasonography is not suitable to evaluate lesion size, and should not be used for measuring methods.Ultrasonography cannot be reproduced completely between two adjacent observations; moreover, the results are dependent on the examiner. Therefore same technique and same measurement results cannot be assured from one to another examination (see Appendix II for details).If new lesions are found by ultrasonography during research, it is suggested to confirm by CT or MRI.Upon worrying irradiation by CT, MRI may be used instead to examine the lesions.

Endoscopy and laparoscopy: it is not suggested that those techniques are used for response evaluation of tumors.However, they are useful in confirming complete pathological response by biopsy or in determining complete response or relapse after surgical resection.

Tumor marker: Tumor markers cannot be used alone to evaluate objective response of tumors.However, when tumor markers are higher than the normal upper limit, they must be standardized if they are used to judge complete response of a patient.As tumor markers are disease-specific, the description for the measurement technique should mark the records of baseline measurement for a special disease.The special guidelines on CA-125 changes (in relapse of ovarian carcinoma) and PSA change (in relapse of prostatic carcinoma) have been published.Moreover, the International Group (Intergroup) of Gynecological Oncology has formulated the criteria for CA125 progression, of which all will be used as firstline criteria for objective evaluation of tumors during trials in ovarian carcinoma.

Cytology and histology: If required by the clinical study protocol, those techniques may be used to distinguish partial response and complete response in individual patients (for example, residual benign tumor lesion in the tumor type of germ cell tumor).When it is known that exudate is a potential serious adverse consequence during treatment (such as some paclitaxel chemotherapeutic drugs or angiogenesis inhibitors), to distinguish effectiveness (such as stable disease) and progression of disease, attention must be paid to development or exacerbation of any new exudate, as proved cytologically during treatment, even though measurable tumors meet the criteria for effectiveness or stable disease.

4. Response Evaluation in Tumors

4.1 Evaluation of All Tumors and Measurable Lesions

To evaluate objective response or incoming potential progression, it is necessary to carry out a baseline evaluation for total tumor burden of all tumor lesions, for reference to subsequent measurements.In clinical protocol with objective response as primary treatment endpoint, only the patients with measurable lesions at baseline may be enrolled.Measurable lesions are defined as presence of at least one measurable lesion.For trials with progression of disease (time to progression of disease or progression degree on fixed date) as primary treatment endpoint, it must be clearly defined in the inclusion criteria in the protocol that it is restricted to only patients with measurable lesions or those with immeasurable lesions may be enrolled.

4.2 Baseline Records of Target and Nontarget Lesions

If there is more than 1 measurable lesions at baseline evaluation, all lesions are recorded and measured; not more than 5 lesions in total (not more than 2 lesions per organ) are used as target lesions, representing all involved organs (that is to say, for a patient with only 1 or 2 involved organs, 2 or 4 target lesions at maximum may be selected as lesions for baseline measurements).

Target lesions must be selected on the basis of their size (maximum diameter), and can represent all involved organs; moreover, measurement must be of good repeatability.Sometimes when the maximum lesion cannot be measured repeatedly, a second maximum lesion may be selected for repeated measurement.

As lymph nodes are normal tissues, they may be visualized even without any tumor metastasis, therefore they must be concerned particularly.Pathological lymph nodes defined as measurable nodes or even being target lesions must comply with the following criteria: The short diameter measured by CT is ≥15 mm.At baseline, only short diameter may be measured.A radiologist usually judges whether a node is with metastasis by the short diameter of the node.The size of a node is generally expressed by the 2-dimensional data of imaging examination (by axial plane in CT, or by one of axial plane, sagittal plane, or coronal plane in MRI).The minimum value is used as short diameter.For example, one abdominal node at 20 mm x 30 mm with a diameter of 20 mm may be considered as a malignant, measurable node.In this example, 20 mm is the measurement of the node.Nodes in a diameter of ≥10 mm but <15 mm should not be considered as target lesion.Nodes in a diameter of <10 mm do not belong to the category of pathological nodes, and are not recorded or subject to further observation.

The sum (containing the maximum diameter of nonnodular lesions, and short diameter of nodular lesions) of the diameters of all target lesions will be used as sum of baseline diameter of lesions and reported.If the diameter of a lymph node is included, as mentioned above, only short diameter are counted in the sum.The sum of baseline diameter will be used as reference value for the baseline level of disease.

Other all lesions including pathological lymph nodes may be considered as nontarget lesions, and need not be measured; however, they should be recorded at baseline evaluation.For example, they are recorded as "present", "absent", or "clearly progressed" in extremely rare cases.Extensively present target lesions may be recorded together with target organs (such as massively proliferated pelvic lymph nodes or large-scale metastasis to liver).

4.3. Criteria for Response Evaluation

In this section, the criteria for measuring objective response rate of target lesions of tumors are defined.

4.3.1. Response Evaluation of Target Lesions

Complete response (CR): All target lesions disappear, and the short axis value of any pathological lymph nodes (whether they are target lesions or not) must be <10 mm.

Partial response (PR): Using the overall diameter at baseline as reference, the sum of the diameter of all lesions is reduced by at least 30%.

Progression of disease (PD): Using the sum of the diameter of minimum lesions (including the sum of the diameter of lesions at baseline, if it is minimum), the sum of the diameter of all target lesions are increased by 20%.Moreover, in addition to the sum of the diameter is increased relatively by 20%, the absolute value of the sum must be increased by at least 5 mm (Note: one or more new lesions occurred may also be considered as progression of disease).

Stable disease (SD): Using the minimum sum of the diameter of the lesions during the study as reference, the lesion reduction does not meet PR, and lesion increase does not meet PD.

4.3.2. Precautions for Response Evaluation of Target Lesions

When the target lesions are lymph nodes:

Generally the measurement of its actual short axis (in the same anatomic plane as baseline) is recorded, even though all lymph nodes are subsided to a size of less than 10 mm.This means when the target lesions are lymph nodes, even meeting the criteria for complete response, the sum of the diameter of target lesions is not 0, as the lymph nodes in a short axis value <10 mm are defined as normal lymph nodes.Nodular target lesions may be recorded seperately in design of the Case Report Form, or the collection method of other data. To judge whether there is complete response, it must meet that the short axis value of each node is < 10 mm.For PR, SD, and PD, the sum of the diameter of target lesions will include the measurement of the short axis of nodes.

Immeasurable target lesions due to too small size:

For all lesions (nodular and non-nodular lesions) recorded at baseline during the study, their measurements must be recorded in subsequent evaluations, despite too small (such as 2 mm).

However, the signals in CT scanning are sometimes too weak when lesions or lymph nodes are recorded at the critical value, and a radiologist may be possibly unwilling to give an accurate measurement, and reported as "too small to be measured".

In such case, it is very important to record the next measurement in the Case Report Form.If a radiologist considers that the lesion may disappear possibly, the measurement may be recorded as 0 mm.If the lesion is present definitely but the signal is too weak, it may be recorded as the default value 5 mm (this rule is not suitable for lymph nodes, as normal lymph nodes are in a definite size value and are often enwrapped by fatty tissues, such as retroperitoneal lymph nodes. However, if the lymph nodes are present definitely but the

signal is too weak and is difficult to measure, they may also be recorded as the default value 5 mm).

The default value 5 mm is originated from the thickness of CT scanning layers (if the thickness is changed, the default value shall not be changed).The measurements of such lesions (too small to be measured) may have no repeatability, and so a default value may prevent any mistaken measurement from which it is evaluated as false cure or false exacerbation.It is emphasized again that if a radiologist may provide an actual measurement, it shall also be recorded as 5 mm, even less than 5 mm.

Ruptured or fused lesions during treatment

According to the notes in Appendix II, when a non-nodular lesion is "ruptured", the sum of the diameter of the target lesions must be calculated by adding up the maximum diameter of all fragments.Similarly, when the lesions are fused, the maximum diameter between them may be kept; in this way, it may help obtain the maximum diameter value of each lesion prior to combination.If the lesions are fused completely and are no longer separated, the maximum diameter vector is the maximum diameter of the fused lesion.

4.3.3 Evaluation of Non-target Lesions

In this Section, the criteria for response of non-target lesions are defined.Although some non-target lesions are measurable actually, it is not necessary to measure them, and it is OK to carry out a qualitative evaluation at the timepoints specified by the protocol.

Complete response (CR): All non-target lesions disappear, and tumor markers are recovered to normal.All lymph nodes are of non-pathological size (the short diameter is <10 mm).

Incomplete response/non-progression of disease (non-CR or non PD): There is one or more non-target lesions and/or the level of tumor markers exceeds persistently the normal level.

Progression of disease (PD): A clear progression occurs in pre-existing non-target lesions.Notes: Occurrence of one or more new lesions may also be regarded as progression of disease.

4.3.4 Special Precautions for Evaluation of Non-target Lesion Progression

The definition of non-target lesion progression is explained additionally as follows: When a patient has measurable non-target lesions, even the target lesions are evaluated as stable disease or partial response, a definition of clear progression made on the basis of non-target lesions must meet the criteria that the whole exacerbation degree of non-target lesions has reached a level that the treatment must be terminated.However, a commonly increased size of one or more non-target lesions cannot reach frequently the criteria for progression; therefore, if the target lesions are stable or are of partial response, it is very rare that the whole tumor progression is defined on the single basis of any change of non-target lesions.

When a patient's non-target lesions are immeasurable: In some Phase III trials, when it is not specified that a measurable lesion is mandatory in the inclusion criteria, such case may occur.The whole evaluation is still carried out with reference to the above criteria; however, as there is no measurable data in such case,it is difficult to evaluate exacerbation of non-target lesions (according to the definition, it is mandatory that all non-target lesions are really immeasurable); therefore, when the change of non-target lesions results in increase of whole disease burden to a level equivalent to occurrence of progression of disease of the target lesions, it is required to establish an effective test method for evaluation, if the definition of clear progression is made, based on non-target lesions.For example, it is described as increased tumor burden equivalent to additional increase by 73% of the volume (equivalent to increase by 20% of the diameter of measurable lesions).For another example, peritoneal exudate is changed from "minor" to "massive", lymphatic disorder is changed from local to "extensively diffusive", or it is described as "sufficient to change the therapeutic method" in the protocol.The examples include: Peritoneal exudate changing from trace to massive, lymph involvement changing from primary site to distant diffusion, or "it is necessary to change the therapeutic regimen" as described in the protocol.If a clear progression is found, the patient is regarded as progression of disease at the timepoint as a whole.It is better to have an evaluation that the objective criteria is suitable for immeasurable lesions. Note: The newly added criteria must be reliable.

4.3.5 New Lesions

Occurrence of new malignant lesions heralds progression of disease; therefore, some evaluation aiming at the new change is very important.Presently, there are no specific criteria for radiological measurement of lesions. Nevertheless, the finding of a new lesion must be clear.For example, progression cannot be attributed to different radiological techniques, change of imaging morphology, or other lesions except tumor (such as some so-called new bone lesions are just healing of original lesions, or relapse of the original lesions).When the baseline lesions develop partial or complete response, this is very important; for example, some hepatic lesion necrosis may be defined as new cystic lesions in the CT report, and are not actually true.

Any lesion detected during follow-up but not detected at baseline is regarded as a new lesion, and suggests progression of disease.For example, a patient is found with visceral lesions at baseline examination, and is found with metastatic lesions at CT or MRI cranial examinations; thus the cranial metastatic lesion of the patient is regarded as evidence for progression of disease, even though he/she does not receive any cranial examination at baseline.

If a new lesion is unclear, for example, due to its small morphology, then a further treatment and follow-up evaluation is required to confirm whether it is a new lesion.If a reexamination confirms that it is really a new lesion, then the time to progression of disease shall be counted from the time when it is found initially.

An FDG-PET evaluation of lesions generally requires additional examinations for supplementary confirmation; the combination of FDG-PET examination and supplementary CT examination results for evaluation of progression (especially new suspected disease) is rational.If a new lesion may be confirmed by FDG-PET, it is carried out in accordance with the following procedure:

The results of FDG-PET examination at baseline are negative, and the results of subsequent FDG-PET examination during follow-up are positive, suggesting progression of disease;

No FDG-PET examination at baseline is conducted, and the results of subsequent FDG-PET examinations are positive;

If a new lesion found by the positive results of FDG-PET examination are consistent with the results of CT, then it is confirmed as progression of disease.

If a new lesion found by the positive results of FDG-PET examination is not confirmed by CT, then another CT has to be conducted (if it is confirmed, then the time to progression of disease is counted from the time when the abnormality is found by the previous FDG-PET examination).

If the positive results of FDG-PET examination are consistent with an existing lesion as indicated by CT, and the lesion is not progressive in radiological examination, then the disease is not progressive.

4.4 Optimal Overall Response Evaluation

Optimal overall response evaluation is the optimal response records from start of the trial to end of the trial, and meanwhile any essential conditions are considered for confirmation.Sometimes response effect occurs at the end of treatment; therefore in the protocol, it shall be clearly specified whether the response evaluation at the end of treatment is considered in the optimal overall response evaluation.In the protocol, it must be clearly specified how the new treatment affect optimal response effects prior to any progression.A patient's optimal response effects are mainly dependent on the results of target lesions and non-target lesions, as well as manifestations of new lesions.Moreover, they are also dependent on trial nature, protocol requirements, and measurement criteria of the results.Specifically speaking, in non-randomized trials, response effect are the primary goal, and PR or CR response confirmation is required, to confirm which is the optimal overall response effect.

4.4.1 Timepoint Response

Suppose that response effects may occur at specific timepoints in each protocol.The summary of overall response effects at each timepoint in the patient population with measurable disease at baseline is provided in Table 1.

See Table 2 for the evaluation, if the patients have no measurable lesions (without target lesions).

4.4.2 Explanation for missing evaluation or unevaluability

If lesion imaging or measurement cannot be conducted at a specific timepoint, then the patient is not evaluable at the timepoint.If only some lesions may be evaluated in an evaluation, generally such case is regarded as not evaluable at that timepoint, unless there is evidence that missing lesions do not impact the response effect evaluation at the specified timepoints.Such case may occur during progression of disease.For example: One patient may have 3 lesions with a sum of 50 mm at baseline; however, only 2 lesions are evaluable later, with a sum of 80 mm. Then the patient will be evaluated as with progression of disease, no matter how much impact the missing lesion has.

4.4.3 Optimal Overall Response: All Timepoints

Once all data of a patient are available, its optimal overall response may be determined.

Evaluation of the optimal overall response when it is not required to confirm complete or partial response effects in the study: During the trial, the optimal response effect is the optimal effect at all timepoints (for example, one patient is evaluated as with SD during response evaluation in the cycle 1, PR in cycle 2, and PD in the final cycle, so his/her optimal overall response is evaluated as PR).When the optimal overall response is evaluated as SD, it must meet the minimum time counted from baseline, as specified in the protocol.If it does not meet the criteria for the minimum time, it is not acceptable even the optimal overall response is evaluated as SD, and the optimal overall response of the patient will be determined, based on subsequent evaluations.For example, one patient is evaluated as with SD in cycle 1 and PD in cycle 2; however, it does not meet the requirements for the minimum time of SD, so his/her optimal overall response is evaluated as PD.Meanwhile, if a patient is evaluated as with SD, and then is lost to follow-up, the patient will be regarded as not evaluable.

Evaluation of the optimal response when it is required to confirm complete or partial response effects in the study: It may be stated as complete response or partial response only when each subject complies with the criteria for partial or complete response, as specified in the trial, and a further response confirmation is conducted at subsequent timepoints (generally 4 weeks later) as mentioned in the protocol.In such case, see the explanations in Table 3 for the optimal overall response.

4.4.4 Special Hints for Response Evaluation

When nodular lesions are included in the evaluation of total target lesions, and meanwhile the node size is reduced to "normal" size (<10 mm), they will have a scanning report for lesion size.To avoid overestimation of the condition reflected on the basis of increased node size, the measurement results will be recorded, even though the node is normal.As mentioned previously, this means subjects with complete response will not be recorded as 0 in CRF/ECRF.

If it is required to confirm response during trial, optimal response evaluation will be complicated by repeated "immeasurable" timepoints.The analysis plan of the trial must

clarify that, those missing data/evaluations may be explained clearly in determining response.For example, in the majority of trials, the PR-NE-PR response of a patient may be regarded as response confirmation obtained.

When the whole healthy condition of a subject develops exacerbation and it is required to discontinue medication, but there is no objective evidence, it will be reported as symptomatic progression.Even after termination of treatment, objective progression shall be evaluated as far as possible.Symptomatic progression is not an evaluation description of objective response: It is the reason for discontinuation of the treatment.The objective response in those subjects will be evaluated by target and non-target lesions as shown in Tables 1-3.

Those defined as early progression, early death, or non-evaluability are particular cases of the study, and shall be described clearly in each protocol (depending on treatment interval and treatment cycle).

In some cases, it is difficult to differentiate local lesions from normal tissues.If the evaluation of complete response is based on this definition, we recommend to carry out a biopsy prior to response evaluation of complete response of local lesions.When the abnormalities in the radiological examination results in some subjects are regarded as representing lesion fibrosis or scar formation, FDG-PET is taken as a similar evaluation standard to biopsy, and is used to confirm response of complete response.In such case, FDG-PET use shall be described prospectively in the protocol, and meanwhile the reports from specialized medical literatures aiming at such case are used as supportive evidence.However, it must be realized that, it will result in false positive results of complete response evaluation, due to restrictions of FDG-PET and biopsy (including extent of resolution and sensitivity for both of them).

Table 1 Timepoint Response: Subjects with Target Lesions (including non-target lesions or not)

Target Lesion	Non-Target lesions	New Lesions	Overall Response
CR	CR	No	CR
CR	Non CR/Non PD		PR
CR	Not evaluable		PR
PR	Non-progressive or not completely evaluable	No	PR
SD	Non-progressive or not completely evaluable	No	SD
Not completely evaluable	Non-progressive	No	NE
PD	Any situation	Yes or No	PD
Any situation	PD	Yes or No	PD
Any situation	Any situation	Yes	PD

CR = complete response	PR = partial response	SDStable disease	PD = progression of disease<br>NE = not evaluable

In SD patients, at least one follow-up measurement after enrolment must meet the SD criteria, and follow-up and enrolment are separated by at least 6-8 weeks.

Table 2 Timepoint Response - Subjects with only Non-target Lesions

Non-Target lesions	New Lesions	Overall Response
CR	No	CR
Non CR or non PD	No	Non CR/Non PD
Not completely evaluable	No	Not evaluable
Unclear PD	Yes or No	PD
Any situation	Yes	PD

Notes: For non-target lesions, "non CR/non PD" are responses better than SD.As SD has been used more and more as endpoint variable, it is aimed at the situation there is no measurable lesions, to set up the non CR/non PD response.
For unclear progression findings (such as very small, undetermined new lesions, or cystic degeneration or necrosis or original lesions), the treatment may be continued to the next evaluation.If progression of disease is confirmed in the next evaluation, then the progression date is that date when the suspected progression occurs.

Table 3 Optimal Overall Response for confirmation of CR and PR

Overall Response at the first timepoint	Overall Response at subsequent timepoints	Optimal Overall Response
CR	CR	CR
CR	PR	SD, PD or PR[a]
CR	SD	If SD persists for sufficient time, then it is SD, otherwise it is PD.
CR	PD	If SD persists for sufficient time, then it is SD, otherwise it is PD.
CR	NE	If SD persists for sufficient time, then it is SD, otherwise it is NE.
PR	CR	PR
PR	PR	PR
PR	SD	SD
PR	PD	If SD persists for sufficient time, then it is SD, otherwise it is PD.
PR	NE	If SD persists for sufficient time, then it is SD, otherwise it is NE.
NE	NE	NE

Notes: CR is complete response, PR is partial response, SD is stable disease, PD is progression of disease, and NE is not evaluable.Superscript "a": If CR occurs actually at the first timepoint, then for any disease occurs at subsequent timepoints, its response evaluation is still PD at subsequent timepoints (as the disease will occurs again after CR), even though the response evaluation of the subject does not meet the PR criteria.Optimal response is dependent on whether SD occurs at the minimum treatment interval.Nevertheless, sometimes it is evaluated as CR, but a scan suggests that small lesions seem to occur still at subsequent timepoints; therefore actually, the patient's response is PR other than CR at the first timepoint.In such case, the first CR judgment shall be revised as PR, and meanwhile the optimal response is PR.

4.5. Frequency for Tumor Reevaluation

During treatment, the frequency of tumor reevaluation is dependent on the therapeutic regimen, and is consistent with the treatment type and schedule.However, in Phase II

trials with unclear treatment benefits, follow-up every 6-8 weeks (the time designed at the end point of one cycle) is rational, and the time interval length may be adjusted in special regimens or situations.In the protocol, it shall clearly indicate tissue sites requiring evaluation at baseline (generally those tissue sites closely related possibly with metastasis of the investigated tumor type), as well as the frequency of repeated evaluation.Under normal circumstances, target and non-target lesions shall be evaluated in each evaluation. Under some optional situations, some non-target lesions may be evaluated in a relatively low frequency. For example, bone scan may be repeated only when the response evaluation of target lesions are confirmed as CR or bone disease is suspected.

At the end of treatment, tumor reevaluation is dependent on whether the response rate or the time to a certain event (progression/death) is used as an endpoint of the clinical trial.If the time to a certain event (such as TTP/DFS/PFS) is used as an endpoint, then it requires regular repeated evaluation as specified in the protocol.Especially in randomized comparative trials, the predefined evaluations shall be listed in the schedule (such as 6-8 weeks during treatment, or 3-4 months after treatment), and be not impacted by other factors, such as delayed treatment, administration interval, or any other events that may result in imbalance between treatment arms in terms of selection of disease evaluation times.

4.6. Confirmation of Response Evaluation/Duration of Response

4.6.1. Confirmation

For non-randomized clinical studies with response as the primary study endpoint, PR and CR responses must be confirmed, to guarantee that responses are not resulted from mistaken evaluation.This also allows rational interpretation of the results if historical data are available; however, the responses in the historical data of the trials should have also been confirmed.Under all the other circumstances, such as randomized trials (Phase II or III) or studies with stable disease or progression of disease as primary study endpoints, response confirmation is not required again, as this is valueless for interpretation of the trial results.Nevertheless, to abolish the requirement for response confirmation, the central review for prevention of bias becomes more important, especially in non-blinded trials.

In case of SD, at least one measurement complies with the specified SD criteria at the minimum time interval (generally not less than 6-8 weeks) after start of the trial.

4.6.2 Overall Response Period

The overall response period is the period from the time when it complies with the CR or PR criteria (whichever is first measured) to the time when disease relapse or progression of disease is first recorded actually (the minimum measurement recorded during the trial is used for reference of progression of disease).The overall duration of response is the period from the time when the measurement first meets the CR criteria to the time when disease or progression of disease is first recorded.

4.6.3. Stable Disease Period

The stable disease period is the period from the start of treatment (from randomization time in randomized trials) to progression of disease; the minimum sum during the trial is used for reference (if the sum at baseline is minimum, then it is used for reference for calculation).The clinical relevance during stable disease period varies in different studies and for different diseases.If, in a certain trial, the proportion of patients maintaining the minimum stable disease time is used as study endpoint, then the protocol shall describe particularly the minimum time interval between two measurements in the definition of SD.

Notes: The response period and the stable disease period are impacted by follow-up frequency after baseline evaluation.To define a standard follow-up frequency, it does not fall in the range of this Guideline.Multiple factors should be considered for follow-up frequency, such as disease type and stage, treatment cycle, and standard specification, etc.If it requires a comparison between trials, restrictions for accuracy of those measurement endpoints should be taken into account.

4.7. PFS/TTP

4.7.1. Phase II Clinical Trails

This guideline is mainly focused on Phase II clinical trials with objective response as study endpoints.Under some circumstances, the response rate is possibly not the optimal selection for evaluation of potential antitumor activities of a new drug/new regimen.Under such circumstances, PFS/PPF at the demarcation point may be regarded as an appropriate surrogate variable to provide original signals of biological activities of a new drug.However, it is obvious that, in a non-controlled trial, those evaluations will be questioned, as seemingly valuable observations may be related with biological factors such as screening of patients, other than the effects of drug intervention.Therefore, randomized, controlled design had better be made in Phase II clinical trials with those variables as study endpoints.However, some tumors are of consistent manifestations (generally poor condition), and so non-randomized trials are also rational.However under such circumstances, it requires to record carefully response evidences during evaluation of predicted PFS or PPF, due to absence of positive control.

See the English version for subsequent contents, including Phase III evaluation endpoints, independent evaluation, reporting of results, Appendix I, and Appendix II.

Appendix 3 Sketch of Safety Data Reporting

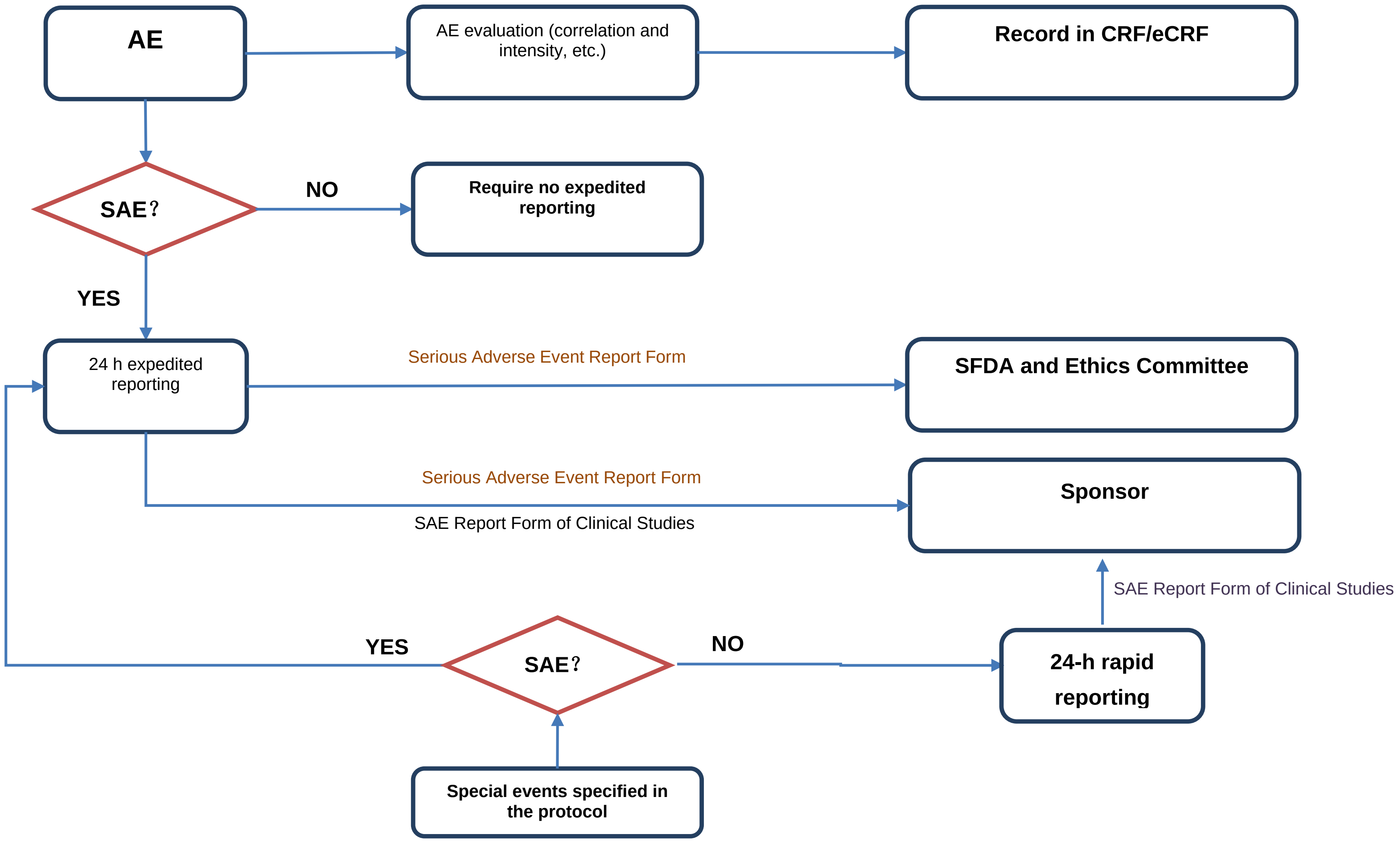

Appendix 4 Clinical Evaluation of Hepatic Impairment

As described in Section 7.1.3, for special events as specified in the protocol, such as concurrent increased ALT/AST + increased total bilirubin, repeating blood biochemistry and increasing monitoring frequency are required, to further describe change trend of biochemical variables.Moreover, it is necessary for the Investigator to rule out other reasons leading to abnormalities of the variables, by inquiring medical history, physical examination, and relevant auxiliary examinations.

Common reasons causing hepatic impairment need to be considered are:

- Acute viral hepatitis
- Alcoholic and autoimmune hepatitis
- Biliary disorder
- Cardiovascular reasons

It is also necessary to consider other uncommon reasons.

It is suggested that the Investigator shall obtain the following information, to further evaluate, follow up and complete clinical data:

- ♦ Obtain medical history
- ✧ Detailed history of current symptoms, as well as concurrent diagnosis and medical history
- ✧ Prior medical history (virus hepatitis, alcoholic hepatitis, autoimmune disease, biliary disorder, and cardiovascular disease, etc.)
- ✧ History of concurrent medications (including over-the-counter drugs and prescription drugs, herbal medicines, as well as dietary supplements), alcohol consumption, recreational drugs, as well as special diets
- ✧ Exposure history to chemical substances
- ♦ Complete the following laboratory tests:
  - ✧ Blood routine
  - ✧ Clinical Chemistry:

    ALT, AST, bilirubin (including total bilirubin and direct bilirubin), alkaline phosphatase, albumin, PT or INR, amylase, fasting blood glucose, cholesterol, and triglycerides
  - ✧ Serum:

    Hepatitis A (Anti-IgM, Anti-IgG), hepatitis B (HbsAg, Anti-HBs, DNA), hepatitis C (Anti-HCV; if positive, it requires RNA test), hepatitis D (Anti-IgM, Anti-IgG),

hepatitis E (Anti-HEV, Anti-HEV IgM), and antinuclear antibodies

- Complete relevant auxiliary examinations:

- For those with concurrent increased ALT/AST + increased total bilirubin as confirmed, an abdominal ultrasonography or other radiological examinations clinically applicable must be performed with 48 hours (to rule out biliary, pancreatic, or intrahepatic reasons, such as biliary stones or tumor); obtain radiological results of liver.If, based on imaging, it cannot determine the reasons (such as biliary, pancreatic, or intrahepatic disease cause) for abnormalities of liver test, it is suggested that a puncture pathology test is conducted after obtaining consent by the subject;

- For those suspected from cardiovascular reasons, it is suggested to carry out an echocardiography to rule out cardiovascular dysfunction (including right heart failure, etc.);

Long-term follow-up: The subjects are monitored closely by repeating ALT, AST, and bilirubin (including total bilirubin and direct bilirubin), once per week, until laboratory ALT and/or AST abnormalities are stabilized or recovered to normal, and then proceed in accordance with the protocol.Those data are reported by electronic case report form.

Appendix 5 Shipment and Processing of Pharmacokinetic Samples

Blood sample

The blood sampling at baseline is performed within 10 minutes after administration according to the time point indicated in the schedule. The accurate sampling time, date of collection and sampling tube number should be recorded in CRF/eCRF at each intravenous sampling. The label of test tube contains the following information: protocol number, subject number, abbreviation of subject's name, administration period, study day and sampling time (hour). 2.5 ml blood will be collected each time, transferred into a test tube with heparin lithium anticoagulant or treated with heparin and labeled, and adequately mixed.

Sample processing and storage:

The blood collected is placed in the ice bath immediately, the plasma is separated after centrifugation for 10 minutes at a rate of 3000rpm under 4℃, the separated plasma is split into two clean 1.5ml sample tubes immediately (about 0.5ml plasma in each tube, the sample tube used shall comply with the requirement), light exposure shall be avoided during operation, the tubes are transferred to -80℃ or -20℃ depot for storage immediately after the lid is tightened (and ensure the transfer to the test center within 14 days). The process of the whole blood sampling and plasma collection is completed within 30 minutes.

Sample Shipment:

When the samples need to be transferred, they must be shipped in the thermotank with dry ice.
