# Supplementary material for: Safety and efficacy of fruquintinib in patients with previously treated metastatic colorectal cancer: a phase Ib study and a randomized double-blind phase II study
Source: J Hematol Oncol. 2017 Jan 19;10:22. doi: 10.1186/s13045-016-0384-9 (PMC5244709; doi:10.1186/s13045-016-0384-9)
Supplement: Additional file 2: — Protocol for phase II study (NCT 02196688). (PDF 1031 kb) [file 13045_2016_384_MOESM2_ESM.pdf]

Front Page of Clinical Study Protocol of Fruquintinib
CFDA Approval No.: 2013L01502, 2013L01503

Study Protocol

Study Title: A randomized, double-blind, placebo-controlled, multicenter Phase II clinical trial to compare the efficacy and safety of Fruquintinib plus best supportive care (BSC) versus placebo plus BSC in patients with metastatic colorectal cancer who have progressed after second-line and above standard chemotherapy

Short Title: A randomized, double-blind, placebo-controlled, multicenter Phase II clinical trial to study the efficacy and safety of Fruquintinib in patients with advanced colorectal cancer

Protocol No.:	2012-013-00CH1
Study Drug:	Fruquintinib (HMPL-013)
Study Objectivives:	Efficacy and safety
Principal Investigator:	Jin LI
Co-principal Investigator:	Ruihua XU
Sponsor:	Hutchison Medi Pharma Ltd.

Statement of proprietary: The Protocol is a property owned by Hutchison Medi Pharma Ltd., and it shall not be spreaded, copied or published without prior authorization of the sponsor.

Clinical Study Protocol Signature Page_01

Signature of the Medical Head from the Sponsor

Your signature on this page indicated that the final Clinical Study Protocol is accepted.

Name: Title:

Signature: Date:

Clinical Study Protocol Signature Page_02

Signature of the investigator

Your signature on this page indicated that the final Clinical Study Protocol is accepted.

Name:

Signature: Date:

(Urgent) Contact Information of the Sponsor

Should emergent medical condition occur, the Project Leader of the study team can be contacted. If the Project Leader is unavailable, the Drug safety emergency contact can be called at the corresponding telephone number listed below.

	**Name**	**Tel**
Project leader	Songhua FAN	18516082311
Drug safety emergency contact	Xiaoyun XI	18601688469

Table of Content

(Urgent) Contact Information of the Sponsor 4
Table of Content 5
Abbreviations 7
Synopsis 11
Objective Response Rate (ORR), Disease Control Rate (DCR), Overall Survival (OS). 12
1 Background Information 17
1.1. Colorectal Cancer 17
1.2. Fruquintinib 18
1.2.1 Preclinical Evidence of Fruquintinib 18
1.2.2 Phase I Clinical Study Results of Fruquintinib (up to 31st Oct, 2012) 19
1.2.2.1 Maximum Tolerated Dose of Fruquintinib for Subjects with Advanced Malignant Solid Tumor 20
1.2.2.2 Safety and Tolerance 22
1.2.2.3 Clinical Efficacy Observation 27
1.2.2.4 Recommended Dose for the Phase II Clinical Trial 29
1.2.2.5 Pharmacokinetic (PK) Study Result 29
1.2.3 Long-term Toxicity Study Result of Fruquintinib 31
1.2.3.1 Long-term Toxicity Test of 26-week Fruquintinib Oral Administration in SD Rats and 4-week Convalescence 31
1.2.3.2 Long-term Toxicity Study of 39-week Oral Fruquintinib (Preparation) Administration in Beagle Dogs and 4-week Convalescence 31
2 Study Objectives 32
2.1 Primary Objective 32
2.2 Secondary Objectives 32
3 Study Design 33
3.1 Overview 33
3.1.1 Rationale of Study Design 33
3.1.2 Randomization Methods 34
3.1.3 Type of Comparison 34
3.1.4 End of Study 34
3.2 Sample Size Determination 34
3.3 Study Sites 35
4 Study Population 35
4.1 Target Population 35
4.2 Inclusion Criteria 35
4.3 Exclusion Creteria 36
4.4 End of Treatment Criteria 37
5 Evaluation Plan and Procedures 38
5.1 Screening Examination and Qualification Form 44
5.2 Subject Enrollment Procedures 44
5.3 Clinical Evaluation 45
5.3.1 Efficacy 45

5.3.1.1 Efficacy Endpoints 45
5.3.1.2 Efficacy Assessment 45
5.3.2 Performance Status 46
5.3.3 Clinical Safety Evaluation 46
5.3.3.1 Safety Endpoints 46
5.3.3.2 Evaluation of AEs, Safety Laboratory Parameters and Other Test Results 46
6 Study Drugs and Administration 48
6.1 Study Drugs 48
6.1.1. Drug Provider 48
6.1.2. Specifications and Storage Life of the Study Drugs 48
*: The appearance of the placebo is identical to that of the study drug. 48
6.1.3. Labelling of the Study Drugs 48
6.1.4. Storage of the Study Drugs 48
6.1.5. Randomization of the Study Drugs 48
6.1.6. Study Drug Count 49
6.1.7. Study Drug Disposing 49
6.2.1. Dosage and Cycle 49
6.2.2. Mode of Administration 50
6.3 Dose Adjustment during the Study 50
6.3.1. Treatment Principles for Toxicities During the Study 50
6.3.2. Handling of Toxic and Side Effects Possibly Related to Fruquintinib 51
6.3.3. Stipulations for Dose Adjustment Induced by Toxicity of the Study Drugs 54
6.3.4. Overdose 61
6.4 Blinding 61
6.5 Concomitant Treatment 61
6.6 Other Concomitant Treatments 62
6.7 Treatment Compliance 62
7 Safety 63
7.1 Safety Parameters and Definitions 63
7.1.1 Adverse Event (AE) 63
7.1.2 Serious Adverse Events (SAEs) 63
7.1.3 Special Events Stipulated in the Protocol 64
7.2.1 Definition of AE Reporting Time 65
7.2.2 AE Intensity Evaluation 65
7.2.3 Drug-event Relationship 66
7.3 Recording and Reporting of Safety Parameters 67
7.3.1. AE Recording 67
7.3.2. Expedited Reporting of SAEs 71
7.3.3. Report of Special Events Stipulated by the Protocol 72
7.4.1. Follow-up of AE 72
7.4.2. Follow-up of SAEs 73
7.4.3. Follow-up of Special Events Regulated by the Protocol 73
7.5 Emergency Unblinding 73
8 Statistical Analysis Plan 73

8.1 Primary and Secondary Endpoints 73
8.2 Statistical Analysis Method 74
8.2.1. Statistical Model 74
8.2.2. Types of Analysis 75
8.2.2.1 Efficacy Analysis 75
8.2.2.2 Safety Data Analysis 76
8.2.2.3 Interim Analysis 76
8.3 Statistical Analysis Plan 76
8.4 Sample Size 77
9 Data Quality Assurance 78
10 Data Monitoring Committee (DMC) 79
11 Ethics 79
11.1 Local Regulations/Declaration of Helsinki 79
11.2 Informed Consent 79
11.3 Independent Ethics Committee (IEC)/Institution Review Board (IRB) 80
12 Protocol Modification 80
13 Conditions of Study Termination 81
14 Study Documents, CRFs and Records Retention 81
14.1 Investigators Files/Records Retention 81
14.2 Original Documents and Background Materials 81
14.3 Direct Access to the Original Data and Documents 82
15 Study Monitoring 82
16 Confidentiality of the Study Documents and Patients' Records 83
17 Data Publication and Business Confidentiality Protection 83
18 References 83
Appendix 1 ECOG Performance Status 85
Appendix 2. Response Evaluation Criteria in Solid Tumors RECIST Version 1.1 86
Appendix 3. Restricted and Prohibited Drugs and Food 108
Appendix 4. Safety Data Report 110
Appendix 5 Clinical Evaluation of Liver Damage 111

Abbreviations

Terms and abbreviations	Definition
AE	Adverse Event
A/G	Albumin/globulin
ALB	Albumin
ALP	Alkaline phosphatase
ALT	Alanine aminotransferase/glutamic-pyruvic transaminase

ANC	Absolute neutrophil count
APTT	Activated partial thromboplastin time
AST	Aspartate aminotransferase/glutamic-oxalacetic transaminase
AUC $_{0\text{-}24\ \text{hoursr}}$	Area under the concentration-time curve from 0 to 24 hours of medication
BSC	Best supportive care
BUN	Blood urea nitrogen
CEA	Carcino-embryonic antigen
CHOL	Cholesterol
c-MET	Mesenchymal epithelial cells transforming factor
CNS	Central nervous system
CR	Complete response
CRC	Colorectal cancer
CRF/eCRF	Case Report Form/Electronic Case Report Form
CT	Computed tomography
CTC AE	Common Terminology Criteria for Adverse Event
DCR	Disease control rate
DFS	Disease free survival
DLT	Dose limiting toxicity
DMC	Data Monitoring Committee
ECG	Electrocardiogram
ECOG	Eastern Cooperative Oncology Group
EGFR	Epidermal growth factor receptor
EORTC	European Organization for Research and Treatment of Cancer
EC	Ethics Committee
EOT	End of Treatment
FDA	Food and Drug Administration
FDG-PET	Fluorodeoxyglucose positron emission tomography
FIB	Fibrinogen

Glu	Glucose
HFS	Hand-foot syndrome
IC50	Half maximal inhibitory concentration
ICF	Informed Consent Form
ICH	International Conference on Harmonization
INR	International normalized ratio
ITT	Intension to treat
IWRS	Interactive Web Response System
LDH	Lactic dehydrogenase
LOAEL	Lowest Observed Adverse Effect Level
LVEF	Left ventricular ejection fraction
MedDRA	Medical Dictionary for Regulatory Activities
mg	Milligram
mL	Milliliter
MRI	Magnetic Resonance Imaging
MTD	Maximum Tolerated Dose
NCCN	National Comprehensive Cancer Network
NCI	National Cancer Institute
NCI CTC AE	The National Cancer Institute Common Terminology Criteria for Adverse Event
NE	Non evaluable
NOAEL	No observed adverse effect level
NSCLC	Non-small-cell lung cancer
ORR	Objective response rate
OS	Overall survival
PD	Progressive disease or pharmacodynamics
PDGFR	Platelet-derived growth factor receptor
PFS	Progression-free survival
PK	Pharmacokinetics

p.o.	Per os (oral administration)
PR	Partial response
PS	Performance status
PSA	Prostate specific antigen
PT	Prothrombin time
QD	*quaque die*/once daily
RECIST	Response Evaluation Criteria In Solid Tumors
SAE	Serious Adverse Event
SD	Stable disease or Standard deviation
TG	Triglyceride
TKi	Tyrosine kinase inhibitor
TP	Total protein
TSH	Thyroid stimulating hormone
TT	Thrombin time
TTP	Time to progression
ULN	Upper Limit of Normal
VEGF	Vascular endothelial growth factor
VEGFR	Vascular endothelial growth factor receptor
WHO	World Health Organization

Synopsis

Drug Name	Fruquintinib
Protocol No.	2012-013-00CH1
Title	A randomized, double-blind, placebo-controlled, multicenter Phase II clinical trial to compare the efficacy and safety of Fruquintinib plus best supportive care (BSC) versus placebo plus BSC in patients with metastatic colorectal cancer who have progressed after second-line and above standard chemotherapy
Short Title	A randomized, double-blind, placebo-controlled, multicenter Phase II clinical trial to study the efficacy and safety of Fruquintinib in patients with advanced colorectal cancer
Sponsor	Hutchison Medi Pharma Ltd.
Principal Investigator	Jin LI
Co-principal Investigator	Ruihua XU
Sdudy Unit	Totally 8 clinical trials units including Fudan University Cancer Hospital
Planned No. of Subjects	Approximately 70 subjects are planned to be enrolled.
Study Duration	Recruiment period is estimated to be about 7 months.<br>The study duration is estimated from Feb, 2014 to Jun, 2015.
Study Objective	To evaluate the efficacy and safety of Fruquintinib in the treatment of patients with metastatic CRC who have progressed after second line or above standard chemotherapy.
Study Design	This is a randomized, double-blind, placebo-controlled, multicenter Phase II clinical trial to compare the efficacy of Fruquintinib plus BSC versus placebo plus BSC in patients with metastatic colorectal cancer. Approximately 70 subjects will be randomized to either of the following treatment group in a ratio of 2:1:<br>● Fruquintinib 5mg orally, QD, plus BSC.<br>● Placebo plus BSC.<br>Subjects will receive a cycles of 4 weeks of study treatment (1 cycle includes 3 weeks of treatment and 1 week of drug discontinuation). The condition of the tumor will be evaluated using imaging method every 4 weeks during the first 4 cycles until to disease progression (PD) and then will be evaluated every 8 weeks afterwards, until to the PD. Safety parameters include Adverse Event (AE), laboratory changes, vital signs and ECG changes. The tumor treatment and survival follow up after PD should also be recorded.

Investigational Drug/ Active Ingredient Name/ Dose/Route/ Treatment Duration	Fruquintinib Capsule (HMPL-013), 5mg, QD, orally at fastings state, take the drug for 3 weeks and then rest for 1 week.<br>Subjects should receive the treatment until the occurance of the following events:<br>PD confirmed by Response Evaluation Criteria in Solid Tumors (RECIST Version 1.1);<br>Death;<br>Patients cannot return to ≤ NCI CTC AE level 1 or the baseline value within 14 days after drug interruption;<br>Abnormal liver function of NCI CTC AE Grade 4; bleeding ≥NCI CTC AE Grade 3; artery thrombosis with any Grade ; any other Life-threatening adverse reactions of Grade 4;<br>Subjects withdrawl of the Informed Consent Form;<br>Subjects that should withdraw from the study for his/her best intest according to the investigator;<br>Pregnency of subjects during the study;<br>Serious protocol violation<br>Lost of follow up
Control Drug	Placebo
Indications	Advanced colorectal cancer(CRC)
Evaluation	
- Efficacy	**Primary Efficacy Endpoint:**<br>Progression free survival (PFS) (According to RECIST Version 1.1)<br>**Secondary Efficacy Endpoint:**<br>Objective Response Rate (ORR), Disease Control Rate (DCR), Overall Survival (OS).
- Safety	AEs are judged and graded in accordance with the NCI CTC AE Version 4.0 and the Comprehensive safety and tolerance should be evaluated by incidence, severity and outcomes of AEs.<br>The safety endpoints include the overall incidence of AEs, incidence of AEs ≥Grade 3, incidence of SAEs, incidence of AEs that lead to perrmanent drug discontinuation; and incidence of AEs that result in drug interruption or dose adjustment.
Inclusion Criteria	Patients may be entered in the study only if they meet all of the following criteria:<br>1. Fully understand the study and signed the Informed Consent Form(ICF) out of their own will;

	2. Subjects histologically or cytologically diagnosed with advanced CRC who failed at least two lines of standard chemotherapies (including Fluorouracil, Oxaliplatin, irinotecan) previously. (failed chemotherapies is defined as the progressed disease or intolerable toxicity);<br>Notes: a) Each line of treatments for advanced disease include one or more chemo drugs used for ≥1 cycle; b) earlier adjuvant/neoadjuvant therapy is allowed..If recurrence or metastasis occur during the adjuvant/neoadjuvant treatment period or within 6 months after completion of the above treatment, that adjuvant/neoadjuvant therapy is considered an first-line systemic chemotherapy for progressive disease; c) chemotherapy combined with EGFR inhibitors such as cetuximab or panitumumab or VEGF inhibitors such as bevacizumab or other target drugs is allowed as pre-treatment; d) standard chemotherapy regime must include use of fluorouracil, oxaliplatin and irinotecan.<br>3. Subject must not receive any systematically anti-tumor therapies such as chemotherapy or radiotherapy, immunotherapy, biological or hormonal therapy for the past 4 weeks, and not receive any VEGFR inhibitor treatment;<br>4. 18-75 years of age (inclusive);<br>5. Body weight ≥40Kg;<br>6. Performance Status (ECOG PS) ≤1(0-1) and did not show any deterioration in 7 days before enrollment;<br>7. Heart function test: Left Ventricular Ejection Fraction (LVEF) ≥50%(echocardiogram test);<br>8. Evident measurable lesion(s);<br>9. Expected survival of over 12-weeks.
Exclusion Criteria	Patients will not be entered in the study for any of the following criteria:<br>1. Absolute neutrophil count (ANC)<1.5×109/L, blood platelet count (PLT) <100×109/L, or hemoglobin<9g/dL; blood transfusion for the purpose of enrollment is not allowed;<br>2. Serum total bilirubin>1.5 × Upper Limit of Normal (ULN), Alanine transaminase (ALT) and/or Aspartate transferase (AST)>1.5 × ULN (subject to the normal value at each site); ALT and/or AST > 3 × ULN in patients with liver metastases;<br>3. Creatinine clearance rate < 60ml/min;<br>4. Regardless of use of any antihypertensive medication, Systolic pressure >140mmHg and /or diastolic pressure >90mmHg; or subjects requires more than two (inclusive) anti-hypertension medications.<br>5. Clinical significant abnormal electrolyte;<br>6. Urine protein detection with 2+ or above, or 24-hour urinary protein quantity ≥1.0g/24 hours;<br>7. With unrestored toxicity in the previous anticancer therapy (NCI CTC AE >Grade

	1), not full recovary from the previous surgery or the time is less than 4 weeks from the previous anticancer therapy or surgery;<br>8. Central Nervous System (CNS) metastatic disease or prior cerebral metastasis;<br>9. Subjects with other malignant tumors, cervical carcinoma in situ, or basal cell carcinoma within the recent 5 years are excluded.<br>10. Clinically uncontrolled active infection, such as acute pneumonia and active hepatitis B;<br>11. Difficulty in swallowing or known drug malabsorption;<br>12. Duodenal ulcer, ulcerative colitis, intestinal obstruction and other gastrointestinal diseases or other conditions that may lead to gastrointestinal bleeding or perforation according to the investigators' judgment; or with a history of intestinal perforation or intestinal fistula;<br>13. Have evidence or a history of bleeding tendency within two months before the enrollment, regardless of the severity;<br>14. Stroke or transient ischemic attack occurred within 12 months before the enrollment;<br>15. Activated Partial Thromboplastin Time (APTT) and/or prothrombin time (PT) > 1.5×ULN (subject to the normal value range at each site);<br>16. Skin wounds, surgical site, trauma site, severe mucosal ulcers or fracture not completely healed;<br>17. With acute myocardial infarction, severe/unstable angina or received coronary artery bypass surgery within 6 months prior to enrollment; or with a history of arterial thrombosis or deep vein thrombosis;<br>18. Female subject who is pregnant or lactating. Female subjects with childbearing potentials tested positive in pregnancy result before the first study drug treatment;<br>19. Any clinical or laboratory abnormalities unfit to participate in this clinical trial according to the investigator's judgment;<br>20. Serious psychological or psychiatric disorders which may affect the compliance of the study;<br>21. Participated in any other drug clinical trials for the past 4 weeks.
End of Treatment, (EOT)	Subjects are considered completed study or discontinued from study should the following conditions occur:<br>1. Progressive disease;<br>2. Death;

	3. Subject unable to return to ≤ NCI CTC AE Grade 1 or baseline values within 14 days after drug interruption;<br>4. NCI CTC AE Grade 4 of abnormal liver function and NCI CTC AE Grade 3 level or above of bleeding; any severity of Arterial thrombosis; Grade 4 of any other life-threatening adverse event.<br>5. Subject pregnancy;<br>6. In the opinion of the investigator, the subject should be withdrawn from the study for his/her best interest;<br>7. Subjects or their legal representatives request to withdraw from the study;<br>8. Lost to follow up;<br>9. Major Protocol violation.

Statistical Analysis and Sample Size Calculation

Statistical Analysis:

The primary efficacy endpoint of this study is to compare the Progressive Free Survival (PFS) of Fruquintinib combined with best supportive care (BSC) versus placebo plus BSC in patients with advanced colorectal cancer. This is a BSC combined placebo control study and the type of comparison is superiority test. The primary efficacy endpoint is PFS.

PFS is defined as the time interval from randomization to the recording of progressive disease (PD) or death, whichever comes first. PD will be determined by the investigator according to the evaluation result of RECIST Version 1. For patients without PD or death, the date of last tumor assessment will be the censoring date. For patients without tumor assessment after baseline evaluation, the date of randomization will be considered as the censoring date.

The comparison of PFS of the two groups is a log-rank test based upon all ITT population at a two-sided significance level of 0.05. When PFS presents statistical significance under the above-mentioned significance level, the study result can be declared as positive.

The log-rank test result of stratified factors shall be provided as well. The stratified factors include previous chemotherapy (2 vs. ≥3), previous treatment by VEGF inhibitors including Bevacizumab, etc. (yes vs. no) and liver metastasis (yes vs. no). Estimate the MST in two treatment groups by adopting Kaplan-Meier method and provide visually intuitive description to the difference between treatment groups by drawing Kaplan-Meier curve. The estimation of treatment effects will be expressed by the Hazard Ratio (HR) estimated by stratified COX model in a 95% Confidential Interval (CI).

Sample Size Determination

The number of primary endpoint (PFS events) required for the evaluation of efficacy is calculated based on the following assumptions:

- A two-sided significance level of 0.05;
- A 67% test power will be ensured when the true HR of treatment group/control group is 0.5, in other words, the median PFS time is extended from 2 months to 4 months;

- The enrollment rate is 10 subjects per month and the overall enrollment can be completed in 7 months;
- A subject withdrawal rate of 15%;

Under the premise of these assumptions, approximately 70 subjects will be enrolled in this study. 6 months after the end of the enrollment (i.e. enrollment of the last subject), and subjects unblinding will be performed for primary endpoint analysis.

1 Background Information

1.1. Colorectal Cancer

Colorectal Cancer (CRC) is a common malignancy, whose worldwide incidence ranks the third place in both males and females. Its overall incidence rate in the western countries ranks the second place, and its number of new cases and that of mortality ranks the third palce in the US in 2003. According to the International Agency for Research on Cancer (IARC), in Asia, especially economically developed regions, the CRC incidence also increased rapidly, and the number is almost close to that in the western countries. Since 2010, CRC has become the second cancer in morbidity and mortality in China, and its incidence in coastal areas has reached 56/100000, with the morbidity age becoming increasingly younger. In Shanghai, the average annual CRC incidence increase is 4.2%, ranking the second place in all cancers, accounting for 13.08% of its total population.

The most common therapy for advanced CRC is chemotherapy treatment, and combination regimens of various chemotherapy drugs such as fluorouracil/leucovorin (5FU/LV), capecitabine, irinotecan, oxaliplatin and etc. are commonly used. In recent years, monoclonal antibody bevacizumab against vascular endothelial growth factor (VEGF), and monoclonal antibodies cetuximab and panitumumab against the epidermal growth factor receptor (epidermal growth factor receptor, EGFR) are combined with chemotherapy respectively as the first-line treatment of patients with advanced CRC, and patient outcomes have been significantly improved [1-2]. Patients with CRC that are ineffectively treated by first-line therapy are treated with second-line chemotherapy, for which the mechanisms of action of these drugs varies with each other, so the treatment of choice mainly depends on the type of tumor, chemotherapy time, and side effects of the drugs, and the efficacy and safety of some drugs remains for further evaluation [2-3]. In Bayer's regorafinib, a vascular endothelial growth factor receptor (VEGFR) as selective kinase inhibitor for the treatment of advanced CRC ineffectively treated by second-line chemotherapy was approved by the FDA in 2002, with a primary endpoint of median survival 1.4 months longer compared with that of the placebo (6.4m vs 5.0m) and a hazard ratio of 0.773, $p = 0.0051$ [4].

Angiogenesis is the most critical step in the occurrence and progression of malignant tumors. According to a variety of studies, tumor angiogenesis in the tumor region to provide nutrients and deliver the metabolites, and tumor cells can be transferred to the rest of the body through new blood vessels. Therefore, effective inhibition of tumor angiogenesis region can inhibit the growth of tumor cells, and thus reducing the incidence of metastasis. Currently, anti-angiogenesis has become the most promising new strategy for cancer treatment. The development of tumor angiogenesis is

associated with a variety of vascular factors, because in the absence of oxygen, the rapid growing tumor cells can secrete a variety of angiogenic growth factors that stimulate angiogenesis. One important factor is vascular endothelial cell growth factor (VEGF). VEGF is found to be one of the main inducible factors related to tumor angiogenesis, so the VEGF / VEGFR signaling pathway is considered to be one of the most promising targets in molecular targeted therapies [5].

In recent years, small molecule targeting anticancer drugs have been developed successfully, such as highly selective VEGF monoclonal antibody drug bevacizumab for the treatment of advanced CRC and breast cancer, and Sunitinib for renal cell carcinoma and Sorafenib for liver cancer and renal cell carcinoma, not only showing the amazing market prospects, but more importantly, pushing the trend from traditional tumor treatment to individualized comprehensive ones, and many patients derive a control tumor and prolong survival benefit. Among them, the great clinical success of bevacizumab has fully proved the effectiveness of the target VEGF as well as its importance in the research and development of new drugs. Sunitinib and sorafenib are multiple targets (including VEGFR) small molecule kinase inhibitors, with simultaneous inhibition of tumor angiogenesis and cancer growth signaling kinase. But too much kinase inhibitation will result in great side effects. Therefore, the development of selective VEGFR II has become a global hotspot.

Furosemide is developed by Hutchison Medi Pharma Ltd. (hereinafter referred to as "the Sponsor"), with complete independent interllectual property right, a small-molecule compound targeting the VEGF receptor kinase family, and closely related to angiogenesis. Furosemide mainly exerts its function on the VEGFR family transmembrane receptors (VEGFR 1, 2 and 3) in the vascular endothelial cells. According to the tests, half inhibitory concentration (IC50) of Furosemide against VEGFR2, VEGFR1 and VEGFR3 are 35nM, 33nM and 0.5nM respectively, and it has no inhabitation (IC50> 3µM) on the activities of a variety of cell proliferation and cell cycle associated kinases including cyclin-dependent kinases (CDK1, 2, 5), EGFR and mesenchymal-epithelial transition factor (c-Met). Besides, Furosemide also has no significant inhabitation on platelet-derived growth factor receptor (PDGFRb) kinase activity (IC50>1µM). Therefore, with high kinase selectivity, furosemide has a good selectivity at the molecular level.

1.2. Fruquintinib

1.2.1 Preclinical Evidence of Fruquintinib

The direct killing effect of Furosemide is very weak in 13 kinds of cells including primary human umbilical vein endothelial cells (HUVEC) (IC50≥30 µM, with IC50 of 18.7µM on primary HUVEC). Compared with the VEGF-dependent proliferation

of HUVEC (with IC50 of only 1.7 nM), the difference is of more than 10,000 times. Furosemide has high efficiency and low toxicity in the enzymology and cellular levels.

In nude mice models with human colon cancer HT-29, human non-small cell lung cancer NCI-H460 and human renal cancer Caki-1, the doses of Furosemide are 0.77, 1.92, 4.8 and 12 mg/kg (2.5 times incresment)), once daily (QD) oral administration for continuous 3 weeks, furosemide showed a dose-dependent tumor growth inhibition. In refractory tumor models such as malignant melanoma A375, pancreatic BXPC-3, pancreatic Miapaca and hepatoma Bel-7402, the daily doses of 1.5, 5 and 15 mg/kg of furosemide has a significant inhibition of tumor growth. Human gastric carcinoma BGC-823 model is most sensitive to furosemide, and a daily dose of 2 mg/kg almost completely inhibit its growth.

1.2.2 Phase I Clinical Study Results of Fruquintinib (up to 31st Oct, 2012)

The clinical study enrolled a total of 40 cases of malignant subjects, most of which are gastrointestinal cancer (8 cases of CRC, 4 cases of colon cancer, and 2 cases of stomach cancer), followed by 9 cases of breast cancer, 7 cases of lung cancer, 3 cases of thyroid cancer, as well as 1 case of pheochromocytoma, melanoma, pancreatic cancer, neuroectodermal tumor, neuroendocrine carcinoma, adenocarcinoma submandibular, nasopharyngeal cancer, and gallbladder cancer respectively (including 1 case of double primary colon cancer and breast cancer). 18 Chinese males and 22 Chinese females, all of whom were of Han ethnic group, 18-70 years old, between 150-175cm in height, and 36-95Kg in weight. The subjects had a history of cancer between 0.38-11.01 years and their performance status (PS) by Eastern Cooperative Oncology Group (ECOG) were≤1 (with 10 cases of 0 and 30 cases of score 1). All the 40 cases received chemotherapy (or other standard treatment), of which 32 subjects received 3 or more chemotherapy regimens (or other standard treatments). 38 cases were with surgical treatment history while 20 cases had a history of radiotherapy. In the continuous dose group, all the subjects had completed the 28 days of continous medication treatment and safety observation except for one subject (dose level of 6mg, Subject No. 009) withdrew from the study due to dose-limiting toxicity (DLT) (hand-foot syndrome) after continuous medication for 13 days. In the group of "continuous medication for 3 weeks and 1 week of drug interruption" (referred to as 3/1 for short) dose group, 1 subject (6mg 3/1 group, Subject No. 105) withdrew from the study due to DLT (fatigue) after medication continuously for 21 days and drug interruption for 7 days; Another subject (5mg 3/1 group, subject No. 110) was considered as screening failure and withdrew from the study after continuous medication for 11 days, while all the remaining subjects completed the 56 days of treatment and safety observation. Subjects' compliance during the study was good,

and all concomitant medications were consistent with the requirements of the Protocol.

13 subjects completed the single-dose PK study and 37 subjects completed the multiple-dose PK studies, and the PK study results will be summarized separately.

1.2.2.1 Maximum Tolerated Dose of Fruquintinib for Subjects with Advanced Malignant Solid Tumor

DLT did not occur after completing safety and pharmacokinetic (PK) evaluation of both single and a continuously 4 weeks of Furosemide administration in 3 dose levels (1mg, 2mg and 4mg) in accordance with the dose escalation method. Subsequently 2 of the 3 subjects in dose level of 6mg experienced DLT (hand-foot syndrome) respectively on D14 and D28. Therefore, dose escalation was stopped, and another 3 subjects were enrolled in a tolerance study with an intermediate dose of 5mg so as to determine the maximum tolerated dose (MTD). In the 5mg dose level, 2 subjects experienced DLT of thrombocytopenia (level 3) with bleeding tendencies and hand-foot syndrome (level 3) respectively on D24 and D28, both of the toxicities recovered to level 1 within 1 week of drug withdrawal. Therefore, another 3 subjects were enrolled in the next last dose of 4mg, and among the 3 added subjects, 1 subject experienced DLT of level 3 hyperbilirubinemia. Therefore, MTD of Furosemide for QD, continuous medication was preliminarily determined as 4mg in this study for subjects with advanced malignant solid tumors.

Furthermore, considering the time of DLT in 5 cases in the tolerance study mostly occured in the 4th week of continuous medication, and that the half-life of the drug was relatively long (42 hours), the sponsor then explored the "continous medication of 3 weeks and drug interruption of 1 week" method for the treatment of patients with advanced solid tumors. DLT window period is determined as the first two courses of treatment (56 days). Safety and tolerance evaluation of dose levels of 5mg and 6mg “continous medication of 3 weeks and drug interruption of 1 week” were completed. No DLT was observed in the 5mg 3/1 dose level (3 subjects), while 1 subject experienced fatigue of level 3 after 21 days of medication in 6mg 3/1 dose level, and the fatigue did not recover within 7 days of drug withdrawal; and no DLT was observed in the subsequent 3 subjects. As the administration mode of 3/1, both 5mg and 6mg are tolerated doses. Considering the security risk to the subjects, no higher dose was explored. Therefore Furosemide of 6mg can be used as the MTD in the treatment of patients with advanced malignant solid tumors in the administration mode of QD, "continous medication of 3 weeks and drug interruption of 1 week".

In this study, 3 cases of hand-foot syndrome, 1 case of thrombocytopenia with bleeding tendencies, 1 case of increased transaminase (ALT and AST) with elevated serum bilirubin, and 1 case of fatigue were determined as DLT.

Therefore, 4mg was determined as MTD of Furosemide for QD, continuous

medication and 6mg of furosemide was determined as MTD of Furosemide for QD, “continous medication of 3 weeks and drug interruption of 1 week” in the treatment of patients with advanced malignant solid tumors.

See Table 1 for the exposures of each dose level, and Table 2 for the List of DLTs.

Table 1. Dose Exposure

Dose Level	Subjects Number	Cases of DLT
1.0mg QD continous medication	1	0
2.0mg QD continous medication	3	0
4.0mg QD continous medication	16	1
6.0mg QD continous medication	3	2
5.0mg QD continous medication	3	2
5.0mg QD continous medication of 3 weeks and drug interruption of 1 week	8	0
6.0mg QD continous medication of 3 weeks and drug interruption of 1 week	6	1
Total	40	6

DLT: dose limiting toxicity; QD: once daily.

Table 2 List of DLTs

Subject No.	Dose Level	Time of DLT	Medication Day of DLT	Study Completeness	Relationship	Detailed Description
008	6.0mg QD	2011-11-28	D28	Yes	Definitely related	Level 3 hand-foot syndrome
009	6.0mg QD	2011-11-21	D14	Subject withdrawal	Definitely related	Level 3 hand-foot syndrome
011	4.0mg QD	2012-01-04	D28	Yes	Unlikly related	Level 3 hyperbilirubinemia *
014	5.0mg QD	2012-02-27	D28	Yes	Related	Level 3 hand-foot syndrome
015	5.0mg QD	2012-02-24	D24	Yes	Possibly related	Level 3 thrombocytopenia with bleeding tendencies
105	6.0mg continous medication	2012-06-27	Continous medication for 21	Subject withdrawal	Possibly related	Fatigue

Subject No.	Dose Level	Time of DLT	Medication Day of DLT	Study Completeness	Relationship	Detailed Description
	of 3 weeks and drug interruption of 1 week		days and drug interruption for 1 week			

DLT: dose limiting toxicity; QD: once daily.

* Note: Subject No.011 experienced hyperbilirubinemia and increased transaminase of level 3 on D28 after medication. According to the investigator, the hyperbilirubinemia was possibly related with the study drug, and was considered as DLT. However after further examination, treatment and follow up, the hyperbilirubinemia is found to be related to the unobstructness of biliary drainage, and resulted from obstruction of biliary tract, so it is considered to be related to the disease instead of the study drug.

1.2.2.2 Safety and Tolerance

Adverse Event (AE):

1) The incidence of AE in single dose drug escalation study (including washout period) (n = 13)

AE were observed in all dose levels except for dose level 1.0mg, most of which are unrelated or unlikely related to the study drug. AEs related to study drug include diarrhea (15.38%), decreased white blood cell count (7.69%), lower hemoglobin (7.69%), all of which were of level 1-2, so no special treatment were given and the symptoms recovered or alleviated during the wash out period.

2) Occurance of AEs in continuous medication of dose escalation trial

2a) Incidance of AEs in QD, continous medication of 28 dyas in dose escalation group of the tolerance trial (n = 26).

AEs were observed in all the dose levels, and most of them were study drug-related, and recovered or alleviated after symptomatic supportive treatment. The most common AE was hand-foot syndrome (46.16%), followed by increased blood thyroid stimulating hormone (TSH) (38.46%), hypertension (34.61%), decreased white blood cell count, proteinuria and diarrhea (both of which were 30.77%), oral ulcers (26.92%), hoarseness (23.08%), decreased platelet count, rash and fatigue (all of which were 19.23%), decreased blood calcium (11.54%). The majority of the AEs were study drug-related and the incidence of other AEs is lower than 10%.

Most AEs were of NCI CTC AE level 1-2. While AE of level 3 were observed in 11 cases, among which only hand-foot syndrome and diarrhea had a incidence of more than 10% (both of them were 11.54%), while the incidence of all the others were below 10%. According to the investigator, the relationship of the AE with the study

drug cannot be excluded. AEs of level 4-5 were not observed in any dose level.

No SAE was observed in the dose escalation clinical trial with continous 28 days of medication, and only 1 case of DLT (level 3 hand-foot syndrome) was observed in a female subject (of 6mg dose level) after 13 days of continous medication, and the drug was stopped.

2b) AEs in the first 56 days in the administration mode of QD, “continous medication of 3 weeks and drug interruption of 1 week” (n=14)

AEs were observed in all the dose levels, and most of them were study drug-related, and were recovered or alleviated after symptomatic supportive treatment. The most common AE was hand-foot syndrome (85.71%), followed by hoarseness (42.86%), reduced white blood cell count and hypertension (both of which were 35.71%), decreased neutrophil count, ECG T wave abnormalities, joint pain, cough, and fatigue (incidence for all of which were 28.57 %), decreased ECG T wave amplitude, increased blood bilirubin, decreased platelet count, proteinuria, gum pain and diarrhea (incidence for all of which were 21.43%). And the incidence of other AEs was low.

Most AEs were of NCI CTC level 1-2. And AEs of level 3 were observed only in 5 cases, namely: 1 case of hypertension, decreased platelet count, fatigue, nausea, and small bowel obstruction respectively. According to the investigator, the relationship of the AEs with the study drug cannot be excluded. AEs of level 4-5 were not observed in any dose level.

2 subjects withdrew from the study. 1 female subject of dose level 5mg 3/1 group withdrew as screening failture after 11 days of medication due to decreased platelets which may pose impact on the last chemotherapy. Another female subject of dose level 6mg 3/1 group withdrew after 21 days of medication due to fatigue of level 3, and the toxicity did not return to normal after 7 days of drug interruption, so the dose was judged as DLT.

3) Occurance of AEs in the whole study period (N=40)

The whole study period refers to the time period from Jan 26, 2011, when the first subject signed the informed consent form (ICF) to the data summary on Oct 31, 2012. AEs were observed in all the dose levels throughout the whole study, and most of them were study drug related and recovered after symptomatic supportive treatment, while AEs (such as hand-foot syndrome, increased serum TSH, hypertension, stomatitis and hoarseness, etc.) of another 14 subjects receiving the study drug still persist, and are under further follow-up observation.

The most common drug-related AE during the whole study was hand-foot syndrome (77.5%), followed by elevated serum TSH (67.5%) proteinuria (47.5%), hypertension (42.5%), decreased white blood cell count (40%), hoarseness (37.5%), rash, diarrhea, fatigue, and ECG T-wave abnormalities (incidence for all of which were 32.5%), decreased platelet count (27.5%) oral ulcers (25%), joint pain (22.5%), decreased neutrophil count and decreased ECG T wave amplitude (both of which were 20%),

gum pain and cough (both of which were 17.5%), increased amylase and increased blood bilirubin (both of which were 15%), musculoskeletal pain and loss of appetite (both of which were12.5%). While the incidence of all other AEs was lower than 10% (including 10%).

Most AE were of NCI CTC AE level 1-2. 39 cases of level 3 study drug related AEs were observed, most of which occurred in dose levels of 4mg and above. The incidence of level 3 AEs that exceeding 10% were: hand-foot syndrome and hypertension (both of which were 17.5%), decreased platelet count (12.5%). While the incidence of all other AEs was lower than 10%. Level 4 and above study drug related AEs were rare, including 1 case (dose level of 2mg) of level 4 coma, 1 case of death (dose level of 2mg) and 1 case (dose level of 4mg) deceased platelet counts. AEs of level 3 and above were listed in Table 3.

Throughout the whole study peroid, AEs resulting in drug interruption occurred in 13 subjects and drug discontinuation occurred in 9 subjects.

8 cases of SAEs were observed in 7 subjects, namely: 1) Subject No.011 of dose level 4mg experienced acute liver injury (hyperbilirubinemia), and initially judged as drug-related by the investigator. However, through subsequent computed tomography (CT) and endoscopic retrograde cholangiopancreatography (ERCP) and other tests, it was found that the subject developed operative multiple liver metastases and intrahepatic bile duct obstruction, so the AE was considered by the investigator ultimately due to disease progression, and was unrelated to the study drug. 2) Subject No.003 of dose level 2mg experienced coma after 4 days of drug withdrawal due to the occurrence of central nervous system (CNS) symptom of suspected brain metastasis, and died due to respiratory failure. Since brain resonance imaging (MRI) findings showed no confirmed diagnosis of brain metastases, the relationship of the SAE and the study drug could not be excluded according to the investigator. 3) Subject No.022 of dose level 4mg experienced thrombocytopenia related to the study drug according to the investigator, and recovered after treatment. 4) Subject No. 105 in dose level 6mg 3/1 experienced small bowel obstruction after 2 weeks of drug discontinuation. Although the subject had a history of colonoscopy perforation and abdominal widespread metastasis half a year before the enrollment, considering the high drug exposure in the subject, the SAE was determined as possibly related to the study drug by the investigator. 5) in addition, 1 case of upper respiratory tract infection, 1 case of death, 1 case of hydrocele, and 1 case pulmonary infection respectively (both in dose level 4mg) unrelated to the study drug according to the investigator were observed. See SAE List in Table 4.

Table 3. List of AEs above Level 3 in Each Dose Level

AE	CTC Level	2mg	4mg	5mg	6mg	5mg 3 wks on/1 wk off	6mg 3 wks on/ 1 wk off	Total
HFS	3	0	2	1	2	1	0	6
Hypertension	3	0	4	0	0	1	1	6
Thrombocytopenia	3	0	1	1	0	1	0	3
	4	0	1	0	0	0	0	1
Diarrhea	3	0	2	0	1	0	0	3
Irregular menstruation	3	0	1	0	0	2	0	3
Proteinuria	3	0	1	1	0	0	0	3
Increased transaminase	3	0	2	0	0	0	0	2
Fatigue	3	0	1	0	0	0	1	2
URI	3	0	2	0	0	0	0	2
Hyperbilirubinemia	3	0	1	0	0	0	0	1
	4	0	1	0	0	0	0	1
Decreased blood phosphate	3	0	1	0	0	0	0	1
Increased alkaline phosphatase	3	0	1	0	0	0	0	1
Decreased blood sodium	3	0	0	1	0	0	0	1
Decrease of serum chlorine	3	0	0	1	0	0	0	1
Bleeding of artificial anus	3	0	0	0	1	0	0	1

AE	CTC Level	2mg	4mg	5mg	6mg	5mg 3 wks on/1 wk off	6mg 3 wks on/ 1 wk off	Total
SBO	3	0	0	0	0	0	1	1
Coma	4	1	0	0	0	0	0	1
Death	5	1	1	0	0	0	0	2
Total	3	0	19	5	4	5	3	36
	4	1	2	0	0	0	0	3
	5	1	1	0	0	0	0	2

Table 4. List of SAEs

Subject No.	Dose level	Name of AE (PT)	NCI-CTC AE Level	Correlation with the study drug	Outcome
003	2mg	Coma	4	Possibly related	AE sustained
		Death	5	Possibly related	AE sustained
011	4mg	Hyperbilirubinemia	4	Unlikely related	AE sustained
013	4mg	URI	3	Unlikely related	AE terminated
		Death	5	Unlikely related	AE sustained
017	4mg	Acquired hydrocele of tunica vaginalis	3	Unlikely related	AE sustained
022	4mg	Thrombocytopenia	4	Related	AE terminated
026	4mg	URI	3	Unlikely related	AE sustained
105	6mg 3 wks on/ 1 wk off	SBO	3	Possibly related	AE sustained

Laboratory Tests, Vital Signs and Physical Examinations, etc.:

During the course of study, abnormalities of all parameters of laboratory tests including hematology, blood biochemistry, urinanalysis, fecal occult blood, coagulation function, thyroid function and immunologic test, etc. were observed and the abnormalities in some cases were determined to be AE by the investigator. The primary manifestation of abnormal vital signs is increased BP. The BP of all subjects were within 85~180/48~130mmHg; HR were within 45~129 bpm; respiration 9~36 times/min and body temperature 36.0℃ ~38.3℃. The change of body weight in the majority of subjects was within 10%, while 9 cases presented body weight change of more than 10% (all in the 4mg and 5mg dose levels). During ECG monitoring, 20 cases (7 cases in 4mg dose level, 6 cases in 5mg 3/1 dose level, 4 cases in 6mg 3/1 dose level and 1 case in 2mg dose level, 5mg dose level and 6mg dose level respectively) presented changes with clinical significance with primary manifestations of flatness of T wave amplitude, abnormal T wave, depression in ST segment, abnormal ST segment, sinus arrhythmia, sinus bradycardia and sinus tachycardia, etc. No subject presented abnormal QT interval with clinical significance. During UCG monitoring, some subjects presented abnormalities and only the abnormalities of 2 subjects were determined as AE (pericardial effusion in both subjects) by the investigator. The ECOG PS in 9 cases decreased (≥2) compared to that of the baseline level.

1.2.2.3 Clinical Efficacy Observation

The enrolled cases of this study were all subjects with advanced solid tumor ineffectively treated with multi-line chemotherapy or other standard treatment (80% of the subjects were patients with advanced solid tumor, who were ineffectively treated by third-line and above therapies) and the majority of subjects presented metastatic lesion of multiple organs including liver and lung.

As shown in the waterfall curve of efficacy evaluation for target lesion, the majority of subjects (82.35%) presented reduced target lesion to a certain degree after receiving

treatment and 13 of them achieved reduction of target lesion up to more than 30% (32.56 – 100%) compared to that of the baseline level.

Efficacy in ITT Population

Overall efficacy: PR 13 cases (1 case not yet confirmed), SD 15 cases, PD 6 cases, unable to evaluate 6 cases, ORR 32.5%, DCR 70%.

Thereinto, in the administration method of "QD, continuous medication": in dose level 1.0mg, 1 subject was SD, in dose level 2.0mg, both of the 2 subjects were SD, in dose level 4.0mg, 13 of the 15 subjects were SD (≥8 weeks) and above (7 subjects PR and 6 subjects SD), in dose level 5.0mg, 3 subjects were SD and above (1 subject PR and 2 subjects SD), in dose level 6.0mg, 1 subject was PD, and 4 subjects withdrew from the study due to toxicity and were considered as unevaluable.

The administration method of "QD, 3 weeks of medication and 1 week of drug interruption": 6 of the 8 subjects in dose level 5.0mg were SD (≥8 weeks) and above (4 subjects PR and 2 subjects SD), 3 of the 6 subjects in dose level 6.0mg were above SD (1 subject PR and 2 subjects SD). 2 subjects withdrew from the study due to toxicity and were considered as unevaluable.

Efficacy of 34 Evaluable Cases:

Evaluable cases include cases with completion of at least 2 cycles of treatment for efficacy evaluation and cases with presence of PD at any time.

Overall efficacy: 13 cases of PR (1 case not yet confirmed), 15 cases of SD and 6 cases of PD were observed, and the ORR was up to 38.23%, and DCR of 82.3%. Thereinto, 1 subject of dose level 1.0mg and the 2 subjects in dose level 2.0mg were SD, 13 of 15 subjects in dose level 4.0mg were SD and above (7 subjects of PR and 6 subjects of SD), 3 subjects in 5.0mg group were SD and above (1 subject PR and 2 subjects SD), 1 subject in dose level 6.0mg was PD, 6 of the 7 subjects that received efficacy evaluation in the dose level of 5.0mg with 3 weeks of medication and 1 week of drug interruption were above SD (3 subjects of PR, 1 PR not yet confirmed, 2 subjects of SD), 3 of the 5 subjects participated in efficacy evaluation in the dose level of 6.0mg with weeks of medication and 1 week of drug interruption were above SD (1

subject of PR and 2 subjects of SD).

Efficacy in Subjects of 4mg (MTD) Continuous Regimen:

During the whole study, among the 15 subjects in dose level 4.0mg with evaluable tumor, the efficacy evaluation of 7 cases was PR, 6 cases was SD, and only 2 cases was PD; ORR was 46.66% and DCR rate was up to 86.66%.

Efficacy in Subjects of 5mg "3 wks on/ 1wk off":

During the whole study, among the 7 subjects in 5mg "3 weeks of continuous medication and 1 week of drug interruption" with evaluable tumor, efficacy evaluation of 4 cases was PR, 2 cases was SD, and only 1 case was PD; ORR was 57.14% and DCR was up to 85.71%.

While among the 5 subjects in 6mg "3 weeks of continuous medication and 1 week of drug interruption" with evaluable tumor, 1 case was PR, 2 cases was SD, 2 cases was PD; ORR was 20% and DCR was 60%.

Efficacy Evaluation of Subjects with CRC:

A total of 12 subjects with CRC were enrolled in the study (all in dose levels of continuous administration) and 10 of them were evaluable. Among them, 3 cases presented PR, 6 cases presented SD and 1 case presented PD. ORR was 30% and DCR was 90%.

1.2.2.4 Recommended Dose for the Phase II Clinical Trial

In accordance with the result of clinical safety, tolerance and efficacy, the sponsor considers the administration method of Fruquintinib 5mg QD "3 wks on/1wk off" presented superior safety performance and tolerance compared to other dose levels (4mg QD continuous administration and 6mg QD 3 wks on/ 1 wk off) as well as comparatively satisfactory clinical efficacy on multiple advanced tumors. As a result, the recommended dose of this product for Phase II clinical trial is 5mg, QD, 3 wks on/1 wk off.

1.2.2.5 Pharmacokinetic (PK) Study Result

HMPL-013 presents extremely low in vivo clearance rate and comparatively low

degree of tissue distribution, which enables the comparatively high plasma exposure dose with comparatively low dosage of oral HMPL-013 administration, meanwhile, HMPL-013 presents comparatively longer elimination half-life. Within the range of single administration of HMPL-013 1mg-6mg, oral administration presents satisfactory and fast absorption. Linear PK characteristics are met and the dose exposure presents linear increase with the increase of doses.

Stable state is considered after 14 days of continuous administration of HMPL-013. Within the range of 1 mg to 6 mg, the dose exposure under stable state increases linearly with the increase of dose. The comparatively long half-life of HMPL-013 supports the regimen of QD administration which is capable of stabilizing the fluctuation of blood concentration under stable state. However, 3~4 times more of drug accumulation under stable state can be produced compared to the initial administration. This type of accumulation is the result of linear effect superposition of multiple administrations and still conforms to linear PK characteristics after multiple medications.

By adopting the administration method of 3 wks on/1 wk off, the PK performance of HMPL-013 can be approximately summarized as: the first 3 weeks of continuous medication since the first administration is a regular climbing process of stable state after continuous medication and the stable state can be reached basically on Week 2 and maintained to the end of the Week 3; and almost complete clearance of HMPL-013 can be achieved at the following 1-week of drug interruption; And Drug re-administration is another climbing process of stable state, and the time of reaching stable state and the level of new stable state remain almost the same with that of the last cycle; and so is the following cycles. The 1 week of drug interruption is a time of clearance, in which the concentration of HMPL-013 gradually decreases. HMPL-013 presented long clearance time and the mean blood concentration of HMPL-013 in both 5mg and 6mg dose levels after 1-week of drug interruption remained at around 25ng/ml, accounting for a mean percentage of 6% in the peak value of blood concentration after the final medication.

Metabolite M1 was hardly detected in any plasma samples; the exposure of M2

accounts for around only 3%~5% of the exposure of original-formed HMPL-013 under whatever administration method or dosage. Therefore, M1 and M2 are not primary in vivo metabolites and rather not “disproportionate metabolite” inducing safety concerns. The in vivo metabolism of HMPL-013 requires further treatment. M2 presented no abnormal PK performance and its trend and characteristics was similar to original-formed HMPL-013.

1.2.3 Long-term Toxicity Study Result of Fruquintinib

1.2.3.1 Long-term Toxicity Test of 26-week Fruquintinib Oral Administration in SD Rats and 4-week Convalescence

Under the condition of this study, after 182-day continuous Fruquintinib oral administration in SD rats, 0 (excipient), 0.5/0.25, 1.5/0.75, 5/3/1.5 and 10/6mg/kg. Dose of Fruquintinib of no less than 1.5/0.75mg/kg can result in death of animals. The primarily observed toxic reactions under dose of no less than 0.5/0.25mg/kg included symptoms of anodontia, perirhinal pollution, eye secretions, watery stool and reduced activities, etc. as well as reduced speed of body weight increase or even decrease, reduced amount of food intake, increased ALT, ALP, CHOL, TG and BUN; decrease of GLU; hypoproteinemia, positive urine protein and leukocyte, repression of bone marrow multiplication, expanded and thickened biliary ducts; pathological changes in liver, kidney, adrenal gland, thymus gland, spleen and femur toxicity. The above-mentioned toxic reactions failed to achieve complete recovery after 4 weeks of drug discontinuation. The primary target organs of toxicity included liver, kidney, adrenal gland, thymus gland, spleen, bone marrow (sternum) and femur. Drug accumulation was not observed after 91 and 182 days of continuous medication. The minimum dose (LOAEL) resulting in observed toxic reaction was 0.5/0.25mg/kg (the C_{max} of D182 and area under curve [$AUC_{0\text{-}24hr}$] of the plasma drug concentration – time curve within 24 hours after drug administration was 327ng/mL and 3676hr*ng/mL).

1.2.3.2 Long-term Toxicity Study of 39-week Oral Fruquintinib (Preparation) Administration in Beagle Dogs and 4-week Convalescence

Under the conditions of this study, Beagle dogs received continuous oral administration of Fruquintinib for 39 weeks and doses were 0.01, 0.03, 0.1/0.06 and 0.2/0.12 (doses were decreased respectively to 0.06 and 0.12mg/kg since Day 15 of

the treatment period), with a 4-week convalescence. Dose of Fruquintinib of 0.2/0.12 mg/kg can result in death of animals. The primarily toxic reactions included symptoms of reduced activities, watery stool, red stool (hematochezia), reduced amount of food intake, loss of body weight, decreased heart rate, decreased level of leukocyte, hypoproteinemia, hepatic toxicity (vacuolization of liver cells, swelling, granulomatous inflammation, liver cell pigmentation and increased Kupffer cells), renal toxicity (vacuolization of renal tubular epithelial cells and positive urine protein), immunity suppression (thymic atrophy, decreased lymphocytes at the edge area of spleen white pulp, decreased mesenteric lymph node cortex and lymphocytes) and thickened growth at metaphysis of femur. The primary target organs of toxicity included gastrointestinal tract, liver, kidney, the immune system (thymus, spleen and lymph nodes) and femur. No significant drug accumulation or gender difference was observed after continuous medication. The dose without observed significant toxic reaction (NOAEL) was 0.03 mg/kg (the AUC_{0-24hr} on Day 273 was 343±55 hr*ng/mL), which was 0.4 times of the equivalent dose for dogs.

2 Study Objectives

2.1 Primary Objective

To compare the Progressive Free Survival (PFS) of Fruquintinib plus best supportive care (BSC) versus placebo plus BSC in patients with advanced colorectal cancer who have progressed after second-line and above standard chemotherapy.

2.2 Secondary Objectives

- To compare the Overall Response Rate (ORR), Disease Control Rate (DCR) and Overall Survival (OS) of subjects in the two groups.
- To compare the safety and tolerance of subjects in the two groups.

3 Study Design

3.1 Overview

3.1.1Rationale of Study Design

This is a randomized, double-blind, placebo-controlled, multicenter Phase II clinical trial to compare the efficacy and safety of Fruquintinib plus BSCversus placebo plus BSCin patients with advanced colorectal cancer ineffectively treated by second-line and above standard chemotherapy.

After checking eligibility criteria, subjects will be randomized into Fruquintinib plus BSC group (treatment group) or placebo plus BSCgroup (control group) in a ration of 2:1.

Treatment group: subjects will receive Fruquintinib 5mg orally, QD, plus BSCfor 3 wks on/ 1 wk off.

Control group: subjects will receive placebo 5mg orally, QD, plus BSCfor 3 wks on/ 1 wk off. The treatment cycle is 28 days. Subjects' safety evaluation and drug accountability will be made in each treatment cycle. Continuous drug safety monitoring and assessment will be performed during the whole study period (including an observation period within 30 days- after the treatment).

Patients will receive a cycles of 4 weeks of study treatment (1 cycle of study treatment includes 3 weeks of treatment and 1 week of drug discontinuation) or until the occurrence of progressive disease (PD), death, unacceptable toxicity, withdrawal of consent or other conditions that meet the End of Treatment cretieria. The condition of the tumor will be evaluated using imaging method every 4 weeks during the first 4 cycles and then will be evaluated every 8 weeks, until the occurrence of progressive disease. Safety parameters include Adverse Event (AE), laboratory changes, vital signs and ECG changes. Besides, the medication and survival follow up after progressive disease should be recorded.

This study will be divided into 3 periods, Baseline Period, Treatment Period and Follow-up Period, from the start of treatment to PD, post-PD until final death.

Table 5 Time Definition of the 3 Phases of the Study

Screening/Baseline	Treatment	Follow-up
D-21~D1(Before the 1st study drug administration	D 1 of the 1st cycle until EOT	EOT to End of Study.

on D1)

The subject can withdraw from the study in the following 4 conditions:

1. Death
2. Lost to follow-up
3. withdrawal of consent and refuse to provide information afterwards
4. End of Study

3.1.2Randomization Methods

After screening, subjects who meet the eligibility criteria will be randomized into Fruquintinib + BSC or placebo + BSC group in a ratio of 2:1 according to the bliding principles, and no stratified randomization will be performed. The Random Number of each subject will be assigned to the investigators by the interactive web response system (IWRS).

3.1.3 Type of Comparison

This is a placebo-controlled parallel study and the type of comparison is superiority test.

3.1.4 End of Study

The primary endpoint is Progressive Free Survial (PFS), unblinding will be conducted and data will be analysed in 6 months after the enrollment of the last subject.
End of Study requirements: obtain PFS events from all the subjects, or obtain OS events from 80% of the subjects. After the End of Study, the sponsor will continue to provide the study drug to subjects who do not achieve PFS , but data other than SAE will not be collected.

3.2 Sample Size Determination

The number of primary endpoint (PFS events) required for the evaluation of efficacy is calculated based on the following assumptions:

- A two-sided significance level of 0.05;
- A 67% test power will be ensured when the true Hazard Ratio (HR) of treatment group/control group is 0.5, in other words, the median PFS time is extended from 2 months to 4 months;
- The enrollment rate is 10 subjects per month and the overall enrollment can be completed in 7 months;

- A subject withdrawl rate of 15%;
- Under the premise of these assumptions, approximately 70 subjects will be enrolled in this study. 6 months after the end of the enrollment subjects will be unblinded for the final analysis of primary endpoint.

3.3 Study Sites

There will be 8 sites including Fudan University Shanghai Cancer Center.

4 Study Population

4.1 Target Population

The target population of the study is patients with histologically or cytologically confirmed advanced CRC who have progressed after second-line or above chemotherapies (including Fluorouracil, Oxaliplatin and Irinotecan).
Where ineffectively treated is definded as the occurance of PD or untolerable toxicity;
Notes: a) Each line of progressive disease treatments include one or more chemotherapy drugs used for ≥1 cycle; b) Early stage adjuvant/neoadjuvant therapy is allowed. If recurrence or metastasis occur during the adjuvant/neoadjuvant treatment period or within 6 months after completion of the above treatment, that adjuvant/neoadjuvant therapy is considered an early first-line systemic chemotherapy for progressive disease; c) Early stage therapy can be chemotherapy combined with EGFR inhibitors such as cetuximab or panitumumab or VEGF inhibitors such as bevacizumab or other target drugs; d) Standard chemotherapy must include fluorouracil, oxaliplatin and irinotecan.

4.2 Inclusion Criteria

Patients may be entered in the study only if they meet all of the following criteria:

1. Fully understand the study and signed the Informed Consent Form(ICF) out of their own will;
2. Subjects histologically or cytologically diagnosed with advanced CRC who failed at least two lines of standard chemotherapies (fluorouracil, oxaliplatin and irinotecan) previously. (failed chemotherapies is defined as the progressed disease or intolerable toxicity);

Notes: a) Each line of treatments for advanced disease include one or more chemo drugs used for ≥1 cycle; b) early stage adjuvant/neoadjuvant therapy is allowed. If recurrence or metastasis occur during the adjuvant/neoadjuvant treatment period or

within 6 months after completion of the above treatment, that adjuvant/neoadjuvant therapy is considered an first-line systemic chemotherapy for progressive disease; c) chemotherapy combined with EGFR inhibitors such as cetuximab or panitumumab or VEGF inhibitors such as bevacizumab or other target drugs is allowed; d) Standard chemotherapy must include use of fluorouracil, oxaliplatin and irinotecan.

3. Subject must not receive any systematically anti-tumor therapies, such as chemotherapy or radiotherapy, immunotherapy, biological or hormonal therapy for the past 4 weeks, or any VEGFR inhibitor treatment ever;
4. 18-75 years of age (inclusive);
5. Body weight≥40Kg;
6. ECOG Performance Status (ECOGPS) ≤ 1(0-1) and did not show any deterioration within 7 days before enrollemnt;
7. Heart function test: Left Ventricular Ejection Fraction (LVEF) ≥ 50%(echocardiogram test);
8. Evident measurable lesion(s);
9. Expected survival of over 12-weeks.

4.3 Exclusion Creteria

Patients will not be entered in the study for any of the following criteria:

1. Absolute neutrophil count (ANC)<1.5×10^9/L, blood platelet count (PLT) <100×10^9/L, or hemoglobin<9g/dL; blood transfusion for the purpose of enrollment is not allowed;
2. Serum total bilirubin>1.5 × Upper Limit of Normal (ULN), Alanine transaminase (ALT) and/or Aspartate transferase (AST)>1.5 × ULN (calculated according to the normal value range at each site); ALT and/or AST > 3 × ULN in patients with liver metastases;
3. Creatinine clearance rate < 60ml/min;
4. Regardless of use of any antihypertensive medication, Systolic pressure >140mmHg and /or diastolic pressure >90mmHg; or subjects requires more than two (inclusive) anti-hypertension medications.
5. Clinical significant abnormal electrolyte;
6. Urine protein detection with 2+ or above, or 24-hour urinary protein quantity≥ 1.0g/24 hours;

7. With unrestored toxicity in the previous anticancer therapy (NCI CTC AE >level 1), not fully recovered from previous surgery; less than 4 weeks from recent anticancer treatment or surgery;
8. Central Nervous System (CNS) metastatic disease or prior cerebral metastasis;
9. Subjects with other malignant tumors, cervical carcinoma in situ, or basal cell carcinoma within the recent 5 years are excluded.
10. Clinically uncontrolled active infection, for example: acute pneumonia and active hepatitis B;
11. Difficulty in swallowing or known drug malabsorption;
12. Duodenal ulcer, ulcerative colitis, intestinal obstruction and other gastrointestinal diseases or other conditions that may lead to gastrointestinal bleeding or perforation according to the investigators' judgment; or with a history of intestinal perforation or intestinal fistula;
13. Have evidence or a history of bleeding tendency within two months of the enrollment, regardless of seriousness;
14. Stroke occurred within 12 months before the enrollment (including transient ischemic attack);
15. Activated Partial Thromboplastin Time (APTT) and/or prothrombin time (PT) > 1.5×ULN (calculated according to the normal value range at each site);
16. Skin wounds, surgical site, wound site, severe mucosal ulcers or fracture not completely healed;
17. With acute myocardial infarction, severe/unstable angina or received coronary artery bypass surgery within 6 months prior to enrollment; or with a history of arterial thrombosis or deep vein thrombosis.
18. Female subject who is pregnant or lactating. Female subjects with childbearing potentials tested positive in pregnancy result in the first pregnancy test before study drug treatment
19. Any clinical or laboratory abnormalities unfit to participate in this clinical trial according to the investigator's judgment;
20. Serious psychological or psychiatric disorders which may affect the compliance of the study;
21. Participated in any other drug clinical trials for the past 4 weeks.

4.4 End of Treatment Criteria

Subjects are considered completed study or End of Treatment should the following

conditions occur (Among which the first 4 is considered as completed the study while the last 5 is considered as discontinued from the study):

1. Progressive disease;
2. Death;
3. Subject unable to return to ≤ NCI CTC AE level 1 or baseline values within 14 days after drug interruption;
4. NCI CTC AE 4 level of abnormal liver function and NCI CTC AE 3 level or above of bleeding; any severity of Arterial thrombosis; level 4 of any other life-threatening adverse event.
5. Subject pregnancy;
6. In the opinion of the investigator, the subject should be withdrawn from the study for his/her best interest;
7. Subjects or their legal representatives request to withdraw from the study;
8. Lost to follow up;
9. Major Protocol violation.

5 Evaluation Plan and Procedures

See Table 6 for the schedule of the study (Schedule of Activities).

Table 6 Schedule of Activities

**Protocol activities**[22]	**Screening**		**Study Treatment**										**Follow Up Period**	
			Cycle 1				Cycle 2		Cycle 3		Cycle 4	Cycle 5 to EOT	EOT Follow up	Surviva l Follow Up
	≦21 days prior to 1st dose	≦7 days prior to 1st dose	C1D1 （1st dose）	C1D8 （±2days）	C1D15 （±2days）	C1D22 （±2days）	C2D1 （±3days）	C2D15 （±2days）	C3D1 （±3days）	C3D15 （±2days）	C4D1 （±3days）	C5D1+ （±3days）	Within 30 days after EOT	Every two months after EOT （±7days）
Informed Consent[1]	X													
Medical History/Oncology History[2]	X													
Surgery[3]	X													
Current medical history/Baseline Signs and Symptoms		X												
Physical		X		X	X	X	X	X	X	X	X	X	X	

Examination[4]														
ECOG[5]	X	X[5]		X	X	X	X	X	X	X	X	X	X	
Vital Signs[6]		X		X	X	X	X	X	X	X	X	X	X	
Hematology[7]		X		X	X	X	X	X	X	X	X	X	X	
Blood Chemistry[8]		X		X	X	X	X	X	X	X	X	X	X	
Coagulation[9]		X		X	X	X	X	X	X	X	X	X	X	
Urinalysis[10]		X		X	X	X	X	X	X	X	X	X	X	
stool occult blood test		X		X	X	X	X	X	X	X	X	X	X	
Pregnancy Test (as appropriate)[11]		X											X	
Carcino-embryonic antigen	X						X		X		X	X	X	
12-lead ECG[12]	X						X		X		X	X	X	
thyroid function[13]	X						X		X		X	X	X	
Echocardiogram[14]	X						X							
Tumor Assessment[15]	X						X		X		X	X	X[15]	
Eligibility Assessment		X												
Subject Randomization[1]		X												

6														
Drug Assignment/Dispense/Return[17]		X	X				X		X		X	X	X	
Study Treatment[18]			X	X	X	X	X	X	X	X	X	X		
Concomitant medication/ Concomitant Procedure[19]	X	X	X	X	X	X	X	X	X	X	X	X	X	
Adverse Event[20]	X	X	X	X	X	X	X	X	X	X	X	X	X	X
Anti-Tumor Therapy													X	X
Survival Follow Up[21]														X

Footnotes for Schedule of Activities

1. Informed consent form must be obtained prior to any study related activities.
2. Medical History/Oncology History: Oncology History includes primary diagnosis date of CRC; date of first metastasis; prior treatment type, start/end date, best overall response, date of progressed disease; adverse reaction with severity above 3 grades. Radiation Therapy includes start/end dates and radiation site.
3. Surgery: operations (Diagnostic or therapeutic non-invasive procedures for example Digestive endoscope, Biopsy) must be collected in CRF includes start/end date, name of procedure and operation site.
4. Physical examination includes Height (baseline only), weight, Head, eyes, ears, nose, throat, neck, heart, chest (including lung), abdomen, limbs, skin, lymph nodes, nervous system, and general condition.
5. ECOG：ECOG is performed during screening visits (21 days before 1st dose and 7 days before 1st dose) and on Day -1 (The day before C1D1). If there's

1 or more than 1 increase in score compared to screening, subject doesn't meet Inclusion criteria 6 and will be excluded from study.

6. Vital Signs include blood pressure, heart rate, breath and temperature. For subject with a baseline history of antihypertensive medications, blood pressure should be monitored 3 hours (± 2 hours) after the daily doses of anti-hypertensive medication
7. Hematology includes red blood cells, hemoglobin, platelet count, neutrophils and White blood cell differential absolutes. If neutrophils$\leq 1\times 10^9$/L or platelets$\leq 25\times 10^9$/L, the test frequency should be increased to once every 2 or 3 days
8. Blood chemistry includes total protein (TP), albumin (ALB), globulin (G), A/G, blood glucose, urea nitrogen, creatinine, alkaline phosphatase (ALP), lactate dehydrogenase (LDH), total bilirubin, AST, ALT, calcium, phosphorus, magnesium, potassium, sodium, chloride, pancreatic amylase and uric acid. The subject presents elevation of ALT or AST over 3 times the normal value with normal transaminase at baseline, or elevation of ALT or AST over twice of baseline value with increased transaminase at baseline, close monitoring of blood biochemistry parameters (ALT, AST, ALP and TBiL) should be performed and the frequency of monitoring should be increased (1-2 times/week). creatinine clearance rate should be calculated by using the baseline creatinine values according to the formulations: Ccr = (140 - age) × weight (kg) / [72 × Scr (mg/dl)] or Ccr = [(140 - age) × weight (kg)] / [0.818 × Scr (umol/L)], and the unit of the creatinine clearance should be taken into consideration during the calculation, and that of females should be the calculated value× 0.85)
9. Coagulation includes prothrombin time (PT), activated partial thromboplastin time (APTT), thrombin time (TT), fibrinogen (FIB) and international normalized ratio (INR)
10. Urinalysis: pH, specific gravity, protein, urinary casts, blood cells, urine glucose and urine ketone. If urinary protein is in the level of + + or above during the medication period, the 24-hour urinary protein quantity should be tested within 1 week.
11. All female patients of childbearing potential must complete blood pregnancy test at screening and within 30 days after EOT. Pregnancy test should be repeated when there's suspected pregnancy. This is not applicable for female subjects in Postmenopausal. Document date of Menopause for female subjects in Postmenopausal.
12. Includes PR interval, QRS time, QT interval, QTc, and diagnostics
13. Includes serum free triiodothyronine (fT3), serum free thyroxine (fT4) and thyroid stimulating hormone (TSH)
14. Includes left ventricular ejection function and assessment
15. Baseline Tumor assessment should be completed within 3 weeks of first dose. CT/MRI Scans of check, abdomen and pelvis are required. Tumor

assessment is performed per RECIST 1.1. Baseline and follow up assessment should use the same imaging method and performed by the same investigator. Tumor assessments are performed on C2D1, C3D1, C4D1, C5D1, and every other cycle afterwards (C7D1, C9D1) until progressed disease. Tumor assessment schedule is calculated from C1D1, won't be impacted by dose interruption. If tumor assessment is not performed within 28 days of last dose, tumor assessment should be completed at EOT. Subject with bone lesion at baseline should be followed by bone scan at subsequent tumor assessments. For suspected cases of disease progression before the start of the next assessment, additional tumor assessment should be performed.

16. Subject randomization: after verifying subject eligibility, site will log into IWRS and randomize subject to treatment arm on Day-1. A subject randomization number is obtained. At the same time, drug assignment is performed in IWRS. An IP serial number is obtained. Site will take IP of the serial number from inventory and on C1D1 dispense IP to subjects. Subject should take 1st dose on C1D1.
17. Drug Assignment/Dispense/Return: subject should maintain all untaken drug and drug container return to site during study visits. On Day -1, it's only necessary to perform subject randomization and drug assignment. Subject should start 1st dose on C1D1. Subject should return the untaken drugs and containers from the previous cycle on D1 of each following cycle. New drug will be dispensed at the beginning of each cycle. On date of visit, Subject will take drug from previous cycle. If tumor assessment is PD for previous cycle and drug has been dispensed, subject needs to return all untaken drug at EOT follow up. If lab results show AE threshold is met to modify study dose, (for dose modification from 5mg to 4mg) subject needs to return to investigational site and return all untaken drug. Site needs to log into IWRS, modify dose level and reassign drug for subject. Drug with new serials numbers will be dispensed to subjects. If dose modification is from 4mg to 3mg, site should log into IWRS and register dose modification. It's not necessary to reassign new drugs to subjects.
18. Administration Method refers to protocol 6.2
19. Concomitant Medications/Treatments： All Concomitant Medications within 21 days before enrollment must be recorded in the case report forms (CRF), including: generic name of the drug and daily dose; reasons for using this medication; starting and stopping date of medication.
20. Adverse event (AE) should be collected from first dose to 30 days after EOT. AEs and laboratory abnormalities that cannot be restored or unexplained need to be collected till recovery or until they can be explained. SAE is collected from informed consent. Only SAEs related to the study drugs should be collected until 30 days after the last dose.
21. Survival follow-up: every two months after EOT. Subsequent anti-tumor therapy and study-related SAEs will be collected.
22. All follow up and data collection will continue throughout the study.

5.1 Screening Examination and Qualification Form

Written ICF should be provided before any specific study evaluation and operation.

Complete medical history should be recorded during screening:

- Demographics:

  Date of birth, gender, ethnic group/race, body weight and height;

- Previous medical history (Previous medical history that meet the following standards should be collected):

  Excluding the indications of the study drug;

  Conditions exsisting before signing the ICF;

  Medical conditions that are considered to be related with the study;

- Other baseline characteristics:

Baseline medical history related to disease factors of the patient including:

Diagnostic date and stage of CRC;

Time of progressive disease after the first time of medication;

Previous anti-cancer therapies: The start time and duration of previous medication of the first-line chemotherapy/second-line chemotherapy and follow-up treatment regimens (including the best effect, level 3 or above toxicities);

ECOG Performace Status (The evaluation standard is specified in Appendix 1);

All the drug treatment and significant non-drug treatment used within 21 days before enrollment must be recorded in the case report forms (CRF), including: generic name of the drug and daily dose; reasons for using this medication; starting and stopping date of medication or whether this drug will be continued during the study.

5.2 Subject Enrollment Procedures

All the subjects will be assessed by the screening criteria. And the investigator should complete the IWRS worksheet to record the screening by login on the IWRS through the internet.

If the subject meets all the eligibility criteria, randomization can be carried out. <u>The subject will get a random number, and participate in the study.</u>

5.3 Clinical Evaluation

5.3.1 Efficacy

5.3.1.1 Efficacy Endpoints

Primary Efficacy Endpoint: PFS (According to RECIST Version 1.1)

Secondary Efficacy Endpoint:

ORR (According to RECIST Version 1.1)

DCR, of which SD≥8weeks

OS

5.3.1.2 Efficacy Assessment

Tumors should be evaluated according to the standard of RECIST Version 1.1, and the evaluation criteria are specified in Appendix 2. Either CT or MRI Tumor Imaging evaluation method can be used at the discretion of the investigator, but the evaluation methods, machines used and technical parameters should be consistent in the entire study period; if no contraindications are implicated, contrast agents should be used. If tumor assessment is made within 21 days prior to the first dose in the same hospital using the same method and same machine, it can be used as baseline tumor assessment. Baseline tumor assessment should include the chest, abdomen, pelvis, and any other sites with suspected tumor lesions. For patients with bone metastases, bone scan should be used for the follow-up of the lesion.

Record of target lesions: number of lesions, site, description, maximum diameter and lymph nodes minimum diameter of each lesion (except lymph nodes), including the total length of all target lesions.

Tumors will be evaluated using imaging method every 4 weeks in the first 4 cycles, and every 8 weeks thereafter, until the occurrence of PD. Should the subject interrupt the medication due to AEs or other reasons, the evaluation should be conducted as scheduled. If the subject withdrew from the study during the first 28 days due to any safety reasons, tumors can be assessed after drug discontinuation. For suspected cases

of PD before the start of the next assessment, additional tumor assessment should be made.

5.3.2 Performance Status

ECOG PS score: ECOG PS score throughout the entire study period is recommended to be conducted by the same investigator at baseline and at each visit. Details are specified in Appendix 1.

5.3.3 Clinical Safety Evaluation

5.3.3.1 Safety Endpoints

Comprehensive safety of the two groups should be evaluated by severity and incidence of AEs, and classified in accordance with the NCI CTC AE Version 4.0. The safety endpoints include:

The overall incidence of AEs;

The incidence of level 3 and above AEs, incidence of SAEs, Incidence of AEs that lead to drug withdrawal, and incidence of AEs that result in drug interruption or dose adjustment.

5.3.3.2 Evaluation of AEs, Safety Laboratory Parameters and Other Test Results

The clinical safety of the study treatment should be evaluated according to NCI CTC AE Version 4.0 throughout the study period.

The occurrence of adverse events of the subjects should be assessed at each clinic visit.

The start time of AE, the highest degree of NCI CTC AE, end time, and its relationship to the study drug, impact to the study, whether additional treatment is given, and recovery should be recorded in the electronic case report form (eCRF).

Physical examination should be conducted at baseline and at each visit, or more frequently according to clinical indications, and should include vital signs (heart rate, blood pressure, body temperature and respiration), body weight and other related organ systems.

12-lead ECG examination should be performed at baseline, on Day 1 of each treatment cycle and 30 days after EOT.

Echocardiography examination should be performed at baseline and on Day 1 of the second treatment cycle.

Carcino-embryonic antigen (CEA) examination should be performed on Day 1 of the first treatment cycle and 30 days after EOT.

Hematology and urinalysis should be performed at baseline, treatment period and the observation period after EOT according to the study schedule.

Test parameters should include:

- Hematology: red blood cells, hemoglobin, neutrophils, platelet count and WBC classification; thrombin time (TT), prothrombin time (PT), activated partial thromboplastin time (APTT), fibrinogen (FIB) and international normalized ratio (INR);
- Urinalysis: pH, specific gravity, protein, urinary casts, blood cells, urine glucose and urine ketone;
- Stool occult blood test;
- Blood clinical biochemistry: total protein (TP), albumin (ALB), globulin (G), A/G, blood glucose, urea nitrogen, creatinine, alkaline phosphatase (ALP), lactate dehydrogenase (LDH), total bilirubin, AST, ALT, calcium, phosphorus, magnesium, potassium, sodium, chloride, amylase and uric acid;
- Routine thyroid function test: should at least include serum free triiodothyronine (fT3), serum free thyroxine (fT4) and thyroid stimulating hormone (TSH);
- 12-lead ECG: including PR interval, QRS time, QT interval, QTc, and diagnostics;
- Particular attention should be paid to left ventricular ejection function evaluation in the echocardiography examination.

6 Study Drugs and Administration

6.1 Study Drugs

6.1.1. Drug Provider

The study drug Fruquintinib capsule and the placebo used in this study will be provided by the sponsor.

6.1.2. Specifications and Storage Life of the Study Drugs

Fruquintinib and corresponding placebo will be manufactured and packaged by Wuxi AppTec Co., Ltd. as authorized by the sponsor. The sponsor should be responsible for the technical instruction and quality control.

Table 7 Information of the Study drugs

Name	Dosage Form	Specification	Administration Method	Storage life
Fruquintinib	Capsule	5mg	Oral	2 years
Fruquintinib	Capsule	1mg	Oral	2 years
Placebo*	Capsule	5mg	Oral	2 years
Placebo *	Capsule	1mg	Oral	2 years

*: The appearance of the placebo is identical to that of the study drug.

6.1.3. Labelling of the Study Drugs

For details of sample packaging and labeling, see the Investigator Site File.
The drug number on the packaging of the study drug should be unique.
Re-supply of all study drugs during the study will be administered by the IWRS system, and details are specified in the IWRS manual.

6.1.4. Storage of the Study Drugs

All the study drugs shall be stored in a closed, safe and cool place according to the requirements.
The storage humidity and temperature should be recorded and kept in the corresponding file.
All the study drugs are for research use only in this study.

6.1.5. Randomization of the Study Drugs

Subjects who meet all inclusion and exclusion criteria confirmed by the investigator will enter the IWRS. IWRS will assign a random number to the patient. The number is

associated with a treatment group, thus a specific treatment is assigned to the subject. And the subject will start the treatment within 1 day after randomization.

6.1.6. Study Drug Count

The investigator/pharmacist/supervisor must keep record of the drugs sent to the site, quantity in the central inventory, drug quantity consumed by each subject, and return all of the remaining drugs to the sponsor or destroy the drugs according to the requirements.

Records described above will include the date, quantity, serial number (drug number), expiration date (used before XXXX) and the study drug and the patient's unique study number. In addition, while returning the remaining drugs to the sponsor, the investigator/pharmacist/supervisor should make sure that the subjects have returned all the unused drugs (well packaged) or remaining drugs (with packages already opened) , and that there is no remaining study drugs at the site.

Drugs used by each subject will be calculated according to the formulation: drugs dispensed– drugs returned– drugs lost.

6.1.7. Study Drug Disposing

All remaining study drugs and the unused drugs collected from the subjects after the end of the study should be returned to the sponsor for destruction; if the destruction is performed by the site, the following written documents should be provided:

Identification of Drug Disposing (drug number or subject treatment assignment).

Number of Disposing

Method of Disposing

Signature and date of the person responsible for destroying the drugs.

6.2. Administration Method

6.2.1. Dosage and Cycle

Treatment group: The subjects will receive oral Fruquintinib 5mg, QD for 3 consecutive weeks and then the therapy will be discontinued for 1 week. The treatment cycle is 28 days.

Control group: The subjects will receive oral placebo 5mg, QD for 3 consecutive weeks and then the therapy will be discontinued for 1 week. The treatment cycle is 28 days.

Subjects will receive the double blinded study treatment according to their dose regimens until the occurrence of PD, unacceptable toxicity, or withdrawal of informed consent.

6.2.2. Mode of Administration

The drugs should be taken at fasting state with 100-200 mL of tepid water. Each subject should record in the patient diary the exact time of drug administration. And it is recommended to take the drugs 1 hour before or 2 hours after breakfast.

During the study, every effort should be made to ensure that the subject take the drugs according to the protocol. Should the subject missed medication in the morning, the the missed dose can be taken on the same day at any time before 22:00. However, if the subjects miss the medication, and failed to take it in the day, he/she must take the next prescribed dose on time, and the missed dose will no longer be taken. Missing dose must be reported to the investigator, and recorded in the CRF. Should vomiting occur within 30 minutes after medication, the drug can be taken again.

Strenuous exercise should be avoided during the study, as well as smoking, alcohol and caffeinated beverages. Grapefruit, pomelo or drinks containing the above fruits should be avoided during the study (see Appendix 3).

6.3 Dose Adjustment during the Study Treatment Principles for Toxicities During the Study

If subject experience any toxicity during the study process, the treatment of toxicity, drug interruption and dose reduction must comply with the following principles. Treatment of toxicity possibly related with Fruquintinib is specified in Section 6.3.2. And dose adjustment due to Fruquintinib related toxicity is specified in Section 6.3.3.

- Should intolerable toxicity occur during the drug administration treatment, drug interruption should be considered first; if the value can return to baseline within 14 days, the dose can be continued or reduced to the previous dose (for detailed dose adjustment information, see Table 8, Table 8-1 through Table 8-6 in Section 6.6.3); if the value cannot be restored to baseline within 14 days, it should be considered end of the treatment period, and the subject should enter follow-up period;
- Should several AEs occur simultaneously, the dose should be adjusted based on the most serious one;
- The time of drug interruption should not be too long, in principle, medication can be continued when the toxicility returned to less than level 1;
- In each dosing cycle, the dose can be adjusted according to the intolerable toxicity. The dose reduced cannot be adjusted again to the previous level; A maximum of

two dose adjustments is allowed for each subject (it can be reduced to 4 mg QD 3/1 at the first time, and 3mg QD 3/1 at the second time); if the dose is adjusted to 3mg QD 3/1, other dose adjustment is not allowed, but dose interruption is allowed.

- Should dose interruption or dose reduction occur, the treatment cycle will not be adjusted in principle. Total drug administration should not exceed 21 days while drug interruption should not exceed 14 days. Should drug interruption is between 7-14 days during 1 drug administration treatment period, a complementary drug administration of no more than 5-day doses can be considered during the drug interruption period (in the last 7 days), but drug administration is not allowed in the last two days of drug interruption period.

6.3.2. Handling of Toxic and Side Effects Possibly Related to Fruquintinib

The toxic and side effects possibly related to Fruquintinib according to the investigator shall be processed with corresponding intervention measures by following the principles of treatment stipulated in Section 6.3.1. The result of Phase I tolerance study has indicated that the Fruquintinib-related adverse reactions mainly include Hand Foot Syndrome (HFS), hypertension, proteinuria, diarrhea, stomatitis, thrombocytopenia and elevated TSH, etc.

Handling of Acute Hypertension

The following procedures shall be referred to in the handling of mild and moderate hypertension:

1. Treatment is not required for transient (<24 hours) diastolic Blood Pressure (BP) or with an increase of >20mmHg or BP >150/100mmHg but ≤180/130mmHg (previous BP normal). The study drug shall be continued.

2. For recurrent or continuous (>24 hours) diastolic BP >150/100mmHg or BP increase of >20mmHg (previous BP normal) or that with detected symptoms, single or multiple kinds of anti-hypertension drugs shall be added for treatment. The study drug shall be continued and BP monitoring shall be performed.

3. For BP>160/100mmHg remaining longer than 1 week after anti-hypertensive treatment (previous BP normal), Fruquintinib interruption shall be considered. Anti-hypertensive treatment shall be continued and close monitoring shall be performed. If the hypertensive condition recovers to Grade 1-2 within 14 days, the

investigator and the sponsor shall co-determine whether to continue Fruquintinib therapy(with continuation of anti-hypertension drugs).

4. For BP>160/100mmHg remaining longer than 1 week after re-administration of Fruquintinib, the investigator and the sponsor shall co-determine whether to reduce the dose of Fruquintinib to previous level.

5. For BP>160/100mmHg remaining longer than 1 week after the second dose adjustment of Fruquintinib with combination or adjustment of anti-hypertensive drugs, treatment with the study drug shall be terminated.

Treatment Principles of Mild and Moderate Anti-hypertensive Drugs (see the hepertension treatment guidelines):

- Fruquintinib may result in elevation of BP;
- Calcium channel antagonist is the first choice. If the subject presents contraindication for application of calcium channel antagonist, β blocker is the second choice;
- Angiotensin Converting Enzyme (ACE) inhibitor and angiotensin II receptor antagonists are primarily applicable for patients with both hypertension and proteinuria;
- For BP>140/90mmHg after treatment, consider increasing the dose to adequate level and adopting combination therapy (e.g.: selective β blocker, thiazide diuretic, ACE inhibitor and angiotensin II receptor antagonists) according to the requirements. If BP>140/90mmHg continues after 24 hours, consider increasing the dose of combination therapy to adequate level or adopting a third anti-hypertensive drug.

The following procedures shall be referred to in the handling of severe hypertension:

When diastolic BP increases to ≥110mmHg or systolic BP increases to ≥180mmHg, the following procedures should be followed:

1. Interrupt the study drug and provide hospitalization treatment;
2. Adopt combination therapy of two kinds of anti-hypertensive drugs (including one type of dihydropyridine calcium channel antagonist), or adjust the subject’s previous anti-hypertensive therapy;
3. If damage in target organ occurs, venous anti-hypertensive treatment shall be provided in addition to oral treatment.

4. Invite relevant experts and personnel to perform BP-stabilization and even emergency treatment for the subject;
5. Consider re-administration of the study drug when subject BP are <140/90mmHg. Refer to Step 4 previously.

Proteinuria

If the subject urine protein was detected with 2+ in urinalysis test during the treatment, please collect 24-hour urine. If urinalysis or 24-hour urine protein quantitation is assessed as NCI CTC AE Grade 1, provide close monitoring; if Grade 2 presented with 24-hour urine protein quantitation <2g, provide close monitoring and active treatment; if 24-hour urine protein quantitation ≥2g, interrupt the study drug and continue dose -reducing treatment if toxicity recovers to lower than Grade 1 within 2 weeks; if toxicity does not recover to Grade 1 after 14 days of drug interruption, the study drug shall be terminated.

Hand-foot Syndrome (HFS)

On condition that the subject presents HFS ≤Grade 2, symptom treatment is acceptable. Hands and feet shall avoid friction, pressure and contact with high temperature objects. Keeping the skin of hands and feet moist and applying appropriate uremic frost or cream containing lanolin oil is beneficial for symptom alleviation and focus recovery. Subjects with severe symptoms (especially with pain) can apply Shaoshang Zhitong Ruangao (produced by Wuhan Jianmin Pharmaceutical Groups Corp. LTD and shall be provided by the sponsor) or take oral Diclofenac Sodium Enteric-coated Tablets (Voltaren), etc. for symptom alleviation.

If the subject presents HFS ≥Grade 3 (severe skin reaction including exfoliation, blister, edema with pain and effects on daily life), drug discontinuation shall be performed in accordance with the treatment principle in Section 6.3.1. Drug of previous or reduced dose can be continued if toxicity recovers to lower than Grade 1 within 14 days.

Diarrhea

If the subject presents diarrhea of Grade 1-2, either close monitoring or drug application for improvement of intestinal functions is acceptable; if diarrhea of Grade 3 appears, Loperamide Hydrochlo-ride Capsules (Imodium) and/or other drugs for improvement of intestinal functions are acceptable. The condition can generally

recover in 3 days and the drug can be continued; for subject fails to recover within 3 days, the study drug shall be interrupted.

Mucositis

If the subject presents stomatitis of Grade 1-2 (including oral ulcers, mucositis, gingivitis, throat discomfort and angular cheilitis, etc.), local application of antibiotics (including anaerobic bacteria resistant antibiotics), antifungal agents, mucosal protective agents, local anesthetics, oral antacids as well as Xiguashuang Spray and Yinhuang Jiedu Pian for oral treatment is acceptable, and the study drug can be continued. Avoid application of drugs containing iodine and long-term use of hydrogen peroxide; soft and nonirritating diet is recommended while spicy, acid and irritating food should be discontinued. If the subject cannot swallow food or take liquid diet, parenteral liquid or nutrition support may be required.

If condition of uncured stomatitis after treatment occurs and affects food intake and body weight, the drug should be discontinued; continuous drug of previous or reduced dose is considerable if toxicity recovers to Grade 1 within 14 days.

Hypothyroidism and TSH Elevation

If the subject presents hypothyroidism (with or without clinical symptoms) with clinical diagnostic significance, or continuous TSH elevation with clinical symptoms (with or without decrease of T4), hormone replacement therapy (HRT) is recommended.

Decreased Platelet Count

If the subject presents decreased platelet count ($<70\times10^9$/L), drug discontinuation and re-examination of haematology is recommended; if the platelet count level fails to recover to Level 1 or baseline level, Recombinant Human Interleukin-11 Injection is recommended for platelet elevation; infusion of apheresis platelet is acceptable for subjects of Grade 4.

Handling of other toxic and side reactions shall be performed in accordance with the above-mentioned principles for toxicity reaction. Appropriate process and treatment should be provided considering subjects' best interests.

6.3.3. Stipulations for Dose Adjustment Induced by Toxicity of the Study Drugs

When drug-related toxicity occurs, the toxicity shall be classified according to NCI CTC AE Version 4.0.

And dose of Fruquintinib should be adjusted according to the following preset dose levels:

Dose Level 0<br>(Original dose)	5mg<br>Oral administration at fasting state, once daily	Fruquintinib of 5mg, 1 capsule, or 1 capsule of the corresponding placebo
Dose Level -1<br>(the 1st dose reduction)	4mg<br>Oral administration at fasting state, once daily	Fruquintinib of 1mg, 4 capsules, or 4 capsules of the corresponding placebo
Dose Level -2<br>(the 2nd dose reduction)	3mg<br>Oral administration at fasting state, once daily	Fruquintinib of 1mg, 3capsules, or 3 capsules of the corresponding placebo

The study drugs related toxicities and corresponding dose adjustment regimens are specified in Table 8, among which, toxicities of hand-foot syndrome (HFS), proteinuria, decreased platelet count, bleeding, and abnormal liver function are excluded from that in Table 8 but listed respectively in Table 8-1 through Table 8-6 specially.

Table 8 Dose Adjustment Induced by Toxicity of the Study Drugs

(excluding HFS, proteinuria, decreased platelet count, bleeding, and abnormal liver function)[a]

Grade of AE (NCI CTCAE Version 4.0)	Drug Interruption	Dose Adjustment
Grade 1	drug administration as scheduled	No adjustment
Grade 2	drug administration as scheduled	No adjustment
Grade 3[b]	Interrupt the dose until the toxicity returns to ≤ 1 Grade 1 or baseline level	Reduce to the dose to the last level
Grade 4	Treatment termination	Treatment termination

a: Should any artery thrombosis occur, the treatment should be terminated.

b: including Grade 3 diarrhea and stomatitis, etc. that ineffectively treated by drug therapies, but not Grade 3 menstrual cycle extension.

Table 8-1 Dose Adjustment for HFS

AE Grading Standard	Dose Adjustment	Treatment Opinions
Grade 1: numb, paresthesia, dysesthesia, erythema, painless edema, thicken skin and hand and foot discomfort which does not affect the normal activities; without any pains	Continue drug treatment of the same dose level	Active treatment can be adopted to relieve the symptoms; for example, urea cream can be used.
Grade 2: erythema with pains accompanied by hand and foot swelling and /or discomfort, which affects normal activities	The drug can be interrupted and reduced to the last dose level should the AE recovers to Grade 1 within 14 days	Shaoshang Zhitong Ruangao manufactured by Wuhan Jianmin Pharmaceutical Group Co, Ltd.
Grade 3: wet decrustation, ulcer, blister or severe hand and foot pain or severe discomfort which affects work or normal activities.	The drug can be interrupted and reduced to the last dose level should the AE recovers to Grade 1 within 14 days	When the same AE occurs for 3 times, the drug should be terminated.

Table 8-2 Dose Adjustment for Proteinuria

AE Grading Standard	Dose Adjustment	Treatment Opinions
Grade 1: Proteinuria + by the urinalysis ; 24-hour urine protein quantitation < 1.0g	Continue drug treatment of the same dose level	Active treatment can be adopted to relieve the symptoms; for example, urea cream can be used.
Grade 2: Proteinuria ++ by the urinalysis ; 24-hour urine protein quantitation is between 1.0-2.0g (excluding 2.0g)	Continue drug treatment of the same dose level	Active treatment and urinalysis should be performed (Every 1 week), accompanied by nephrology consultation if

		necessary.
Grade 2: Proteinuria ++ or above by the urinalysis ; 24-hour urine protein quantitation is between 2.0-3.5g (excluding 3. 5g)	The drug can be interrupted and reduced to the last dose level should the AE recovers to Grade 1 within 14 days	Active treatment should be performed, accompanied by nephrology consultation if necessary; and the drug should be terminated should the AE occur for the 3rd time.
Grade 3: 24-hour urine protein quantitation ≥3.5g	The drug can be interrupted and reduced to the last dose level should the AE recovers to Grade 1 within 14 days	Active treatment should be performed, accompanied by nephrology consultation if necessary; and the drug should be terminated should the AE occur for the 3rd time.

Table 8-3 Dose Adjustment for hypertension (patients that receiving anti-pressure treatment at baseline should monitor blood pressure after drug administration once daily)

AE Grading and Definitions	antihypertensive therapy	Dose of Fruquintinib
Grade 1: early period of hypertension: (systolic pressure of 120-139mmHg or diastolic pressure of 80-89mmHg)	None	Continue drug treatment of the same dose level.
Grade 2: systolic pressure of 140-159mmHg or diastolic pressure of 90-89mmHg; or diastolic pressure	Treatment objective: keep the blood pressure at a level of less than 140/90mmHg.<br>If the patient has received the antihypertensive treatment, the dose of the antihypertensive drug	Continue drug treatment of the same dose level.<br>For the use and dose adjustment of antihypertensive drugs, please refer to the

symptomatic increase >20mmHg	should be increased or adopt other antihypertensive therapies; If the patient does not receive any antihypertensive treatment, a single antihypertensive therapy should be used.	antihypertensive drug treatment guideline and invite the nephrology for consultation if necessary.
Grade 2: systolic pressure of 140-159mmHg or diastolic pressure of 90-89mmHg; or diastolic pressure symptomatic increase >20mmHg	Treatment objective: keep the blood pressure at a level of less than 140/90mmHg.<br>Start to use antihypertensive drug or increase the dose of the antihypertensive drug in use or adopt other antihypertensive therapies additionally; For the use and dose adjustment of antihypertensive drugs, please refer to the antihypertensive drug treatment guideline and invite the nephrologist for consultation if necessary.	For patient with blood pressure still exceeds 160/100mm Hg for more than 7 days after using antihypertensive drug or adjusting the dose of the drug in use, Fruquintinib should be interrupted; Should the blood pressure of the patient recover to Grade 1 the baseline level, one time of dose reduction shall be made.
Grade 4: Fetal results (such as malignant hypertension, temporary or permanent neurological deficits and hypertensive crisis)	Emergent medical treatment	The drug should be terminated.

Table 8-4 Dose Adjustment for Decreased Platelet Count

AE Grading	Dose Adjustment	Treatment Opinions
Grade 1: Platelet Count of	Continue drug treatment of the same dose level	Perform follow up visit as scheduled.

$100\sim75\times10^9$/L		
Grade 2: Platelet Count of $75\sim50\times10^9$/L	The drug can be interrupted and reduced to the last dose level should the AE recovers to Grade 1 or baseline level within 7-14 days	Hematology examination should be performed every 2-3 days; active treatment using interleukin-11 is recommended. Hematology examination should be performed once every week in the follow up visit.
Grade 3: Platelet Count of $50\sim25\times10^9$/L	The drug can be interrupted and reduced to the last dose level should the AE recovers to Grade 1 or baseline level within 14 days.	Hematology examination should be performed every 2-3 days; active treatment using interleukin-11 is recommended. Hematology examination should be performed once every week in the follow up visit.
Grade 4: Platelet Count $<25\times10^9$/L	The study treatment should be terminated	Hematology examination should be performed once daily until the AE recovers to Grade 2 or a lower grade; single donor platelets should be provided as well as interleukin-11 active treatment

Table 8-5 Dose Adjustment for Bleeding in Any Site

AE Grading	Dose Adjustment	Treatment Opinions
Grade 1	Continue drug treatment of the same dose level	Perform follow up visit as scheduled.
Grade 2	The drug can be interrupted and reduced to the last dose level should the AE recovers to Grade 1 or lower level within 14 days.	Active treatment
Grade 3 or above	The study treatment should be terminated.	Emergent medical intervention.

Table 8-6 Dose Adjustment for Abnormal Liver Function (increasing of ALT, AST or total bilirubin)

AE Grading	Dose Adjustment	Treatment Opinions
Grade 1	Continue drug treatment of the same dose level.	Perform follow up visit as scheduled.
Grade 2 (with normal baseline value)	The drug can be interrupted and reduced to the last dose level should the AE recovers to Grade 1 or lower level within 14 days.	Active liver protection treatment should be provided, and the liver function should be monitored closely once every week.
Grade 2 (with abnormal baseline value)	Continue drug treatment of the same dose level.	Active liver protection treatment should be provided, and the liver function should be monitored closely once every week.
Grade 3	The drug can be interrupted and reduced to the last dose level should the AE recovers to Grade 1 or lower level within 14 days.	Active liver protection treatment should be provided, and the liver function should be monitored closely (twice every week until the toxicity recovers to Grade 1, baseline level or can be reasonably explained).
Grade 4	The study treatment should be terminated.	Active liver protection treatment should be provided, and the liver function should be monitored closely (twice every week until the toxicity recovers to Grade 1, baseline level or can be reasonably explained).

a: Should total bilirubin >2×ULN and/or aminopherase >3×ULN occurs in patients with normal baseline values, or total bilirubin > 2×baseline value and/or aminopherase >3× baseline value occurs in patients with abnormal baseline values, please report as special event and provide treatment to the patient according to Protocol.

6.3.4. Overdose

If overdose (defined as administration of more than one dose within 24 hours) occurs, symptomatic and supportive treatment should be provided.

If the dose of the study drug exceeds the regulated dose in the study protocol, while no corresponding symptoms or signs presents, it is not required to record it in the CRF but to report as protocol violation. In terms of overdose accompanied by SAE, diagnosis and symptoms of the SAE is required to be recorded in the "SAE Form". If overdose accompanied by AE, not SAE, occurs, the AE shall be recorded in "AE Form".

6.4 Blinding

Randomize the subjects into Fruquintinib treatment group or corresponding placebo treatment group using double-blind method. Identity of the drug administered for treatment remains unknown for investigator, the sponsor and subjects. The allocated randomized numbers are based upon the information provided by IWRS.

Fruquintinib and placebo present identical appearance to ensure the implementation of blinding and the subject shall take 1 capsule of 5mg, PO, QD. For the purpose of blinding, the study drugs (Fruquintinib or corresponding placebo) shall be marked by unique drug numbers which are pre-printed on each package bottle, and be distributed to each subject through the IWRS.

Emergency unblinding: unblinding shall be performed only under emergency circumstances. Investigators should notice that the occurrence of SAE should not be adopted as the regular condition of immediate label unblinding. On condition that unblinding is required for treating SAE of a certain subject, medical monitor from the sponsor must be contacted in advance before unblinding. Unblinding of the subject shall be performed through IWRS system.

6.5 Concomitant Treatment

Subjects are not allowed to receive other simultaneous antineoplastic treatment, including cytotoxic drug (except for non-antineoplastic chemotherapy), radiotherapy (except for palliative radiotherapy for symptom control), biotherapy, endocrine therapy or any other treatment with the study drugs, during enrollment of this study as well as the whole process of this study. TCM with antineoplastic indications is

prohibited as well during the process of this study (see reference Appendix 3). Systemic antineoplastic treatment and treatment with other study drugs must be terminated 4 weeks before the subject's enrollment of this study.

6.6 Other Concomitant Treatments

The investigator should comply with the following guiding principles, cautiously select concomitant medication during the process of study and make every effort to protect the safety of the subject.

- BSC is permitted during the process of study while preventative application of anti-vomiting medicine is prohibited. Application of other drugs that may induce or aggravate the symptoms of clinical study is prohibited and enhanced monitoring is required when application of that drug is necessary.
- Fruquintinib has been proved to be metabolized through hepatic cytochrome P450 3A4 according to pre-clinical study. Strong inducers of enzyme CYP3A4 such as Phenytoin, Phenobarbital, Rifampin and other drugs (not limited to the above-mentioned drugs) as well as strong inhibitors of enzyme CYP3A4 such as Ketoconazole, Itraconazole, Fluconazole, Indinavir, Erythromycin, etc. (not limited to the above-mentioned drugs) may significantly influence the in vivo metabolism of Fruquintinib. Investigators should be cautious for enrollment of the subjects received confirmed combination with inducers and inhibitors of enzyme CYP3A4. If concomitant medication of this type is applied during the study, cautions are required as well as close monitoring of drug exposure and adverse reactions. See Appendix 3.
- Subjects are allowed to take anticoagulant (e.g. Warfarin) during the treatment period while monitoring of relevant coagulation indicators such as INR is required; LMWH Sodium is acceptable when required by the treatment;
- Investigators shall decide whether to apply other concomitant medications to ensure the interests and safety of the subjects.

All the drugs including study drugs applied during the process of treatment should be recorded in the CRF/eCRF.

6.7 Treatment Compliance

Investigators should record the amount and date of study drug distribution and collection, as well as actual dose of administration from each subject in a timely and

accurate manner. The actual dose of administration should be consistent with the dose required in the Protocol. Drug treatment compliance shall be determined according to the amount of study drug distributed and collected from subjects as well as the amount of drug lost by subjects at the end of each treatment period and at the time of study withdrawal. It shall be determined comprehensively as the conditions of self-reported dose missing/overdose/drug losing etc. from the subjects.

The patients are required to return all bottles of used and unused study drugs to the site at the end of treatment for evaluation of compliance. All remaining materials and drugs must be returned to the sponsor at the end of study.

7 Safety

7.1 Safety Parameters and Definitions

7.1.1 Adverse Event (AE)

An AE is any untoward medical occurrence occurring after the patient has received the medication, whether or not considered related to the study drugs. Therefore, an AE can be any adverse or untoward sign (including laboratory abnormalities), symptom or disease. An AE has a temporal relationship with the medication but is not necessarily related to the drug.

In addition, AEs also include complications induced by intervention regulated in the Protocol. For instance, complications induced by biopsy and other invasive operations, and worsening of the disease that considered by the investigator as pre-existed during AE reporting (except for tumor progression) is also considered as AE.

7.1.2 Serious Adverse Events (SAEs)

Serious adverse event (SAE) refers to any AE that meets at least one of the following criteria:

- **Results in death:** AE resulting in death of the subject, excepted for death resulted from progressive disease (PD);
- **Is life-threatening:** this refers to an event in which the patient was at risk of death at the time of the event. It does not refer to an event, which hypothetically might have caused death, if it were more severe.
- **Requires hospitalization or prolongation of an existing hospitalization:** AE results in hospitalization treatment (not including emergency or outpatient treatment),

or that occurs during hospitalization of the subject and prolongs the existing hospitalization;

- **Results in persistent or significant disability or incapacity:** AE results in substantial harm to the subject's capacity of conducting daily life (incapacity does not include events with secondary clinical significance, such as headache, nausea, vomiting, diarrhea, influenza and accidental trauma (e.g. ankle strain, etc.);

- **Is a congenital abnormality or birth defect:** A congenital abnomality or birth defect exists in the newborn (fetus) born (aborted) by a female subject with drug exposure or the female companion of a male subject with drug exposure;

- **Other important medical events:** An important medical event may not cause immediate risk of life, result in death or hospitalization. However, it may jeopardize the subjects or require drug or surgical treatment to prevent the occurrence of the above-mentioned outcomes (death of subjects, life-threatening, result in hospitalization, prolonged hospitalization, result in persistent or significant disability or incapacity, congenital abnormality).

7.1.3 Special Events Stipulated in the Protocol

The sponsor shall provide continuous close monitoring on potential drug-induced liver damage even it is rare.

Close monitoring of blood biochemistry should be performed when the subject presents the following condition:

The subject presents elevation of ALT or AST over 3 times the normal value with normal transaminase at baseline, or elevation of ALT or AST over twice of baseline value with increased transaminase at baseline, close monitoring of blood biochemistry parameters (ALT, AST, ALP and TBiL) should be performed and the frequency of monitoring should be increased (1-2 times/week);

- For subject presenting early symptoms of liver damage (such as anorexia, nausea, vomiting, discomfort in upper right stomach, weakness, etc.) before detection of abnormal blood biochemistry, immediate examination of blood biochemistry should be performed and frequency of monitoring should be increased if above-mentioned abnormalities occur.

Special events regulated by the Protocol are as follows:

- The subject presenting normal hepatic function (ALT, AST and bilirubin are all within their normal ranges) at baseline is tested with AST and/or ALT

elevation >3×ULN combined with TBiL elevation >2×ULN in one blood sample collection.

- The subject presenting increased transaminase at baseline is detected with AST and/or ALT elevation >twice of baseline value combined with TBiL elevation >2×ULN in one blood sample collection.

When subject presents the above-mentioned special event, close monitoring of blood biochemistry parameters (ALT, AST, ALP and TBiL) should be performed and the frequency of monitoring should be increased (1-2 times/week).

In addition, for subject presenting early symptoms of liver damage (such as anorexia, nausea, vomiting, discomfort in upper right stomach, and weakness, etc.) before detection of abnormal blood biochemistry, immediate examination of blood biochemistry should be performed and frequency of monitoring should be increased if the requirements of special events specified in the Protocol are met.

7.2 Collection and Evaluation of Safety Parameters

7.2.1 Definition of AE Reporting Time

Table 9 Definition of AE Reporting Time

Time Period	Reporting Requirements
Since signing the ICF to the time before first administration of the study drug	Report all SAEs
Since administration of the first dose till 30days after the final dose of the study drug	Record all AEs and SAE (including special events regulated by protocol) except for disease progression.
Post-treatment period (since 30 day after the final dose till the end of the study)	Report only treatment emergent SAEs.

7.2.2 AE Intensity Evaluation

The intensity of all AEs shall be graded and determined as 5 grades (Grade 1 to Grade 5) in accordance with NCI CTC AE V4.0. The AEs not listed in NCI CTC AE shall be determined according to the following table (Table 6).

Table 10 Determination of AE Intensity

CTC Grading	Equivalent to	Definition
1	Mild	Discomforts are observed while regular daily activity is not affected.
2	Moderate	Discomforts are sufficient to reduce or affect daily activity: treatment or medical intervention is not adopted, even though they are capable of improving life quality of the patient or remitting symptoms
3	Severe	Incapable of working or fulfilling daily activity, treatment or medical intervention has been adopted to improve life quality of the subject or relieve symptoms, treatment delay will not place the patient in immediate risk of death
4	Life-threatening or incapacitating	With immediate risk of death or permanent mental or physical damage, incapable of working or fulfilling daily activity, treatment or medical intervention is required to sustain life
5	Fatal	AEs resulting in death

Distinction of severity and intensity of AE requires attention. Intensity refers to the intense extent of AEs (e.g.: mild, moderate or severe headache) while the event itself presents comparatively slight clinical significance (for example severe headache) and cannot be determined as SAE unless it conforms to the criteria of SAE. Therefore, severity and intensity should be evaluated independently during AE/SAE report.

7.2.3 Drug-event Relationship

The relationship of the study drug with AE and the role played by the study drug in AE can be classified as 4 categories of unrelated, unlikely related, possibly related and related. The following classification and criteria can be referred to for determination:

1. Unrelated: Other factors (disease, environment, etc.) are determined as the cause after medical judgment.

2. Unlikely related: The study drug is considered unlikely related to AE after medical judgment:

a) No temporal relationship exists between the drug application and occurrence of AE.

b) AE may be caused by other factors such as change of disease course, environment or application of other drugs for treatment.

c) Occurrence of AE is unrelated to the acknowledged characteristics of the drug.

d) AE does not recur or aggravate after continuous administration.

3. Possibly related: (the previous two items are a necessary condition) if the following conditions are met after medical judgment, AE is considered to be possibly related to the study drug.

a) Temporal relationship exists between the drug application and occurrence of AE.

b) The relationship between AE and change of course of disease, environment or application of other drugs for treatment cannot be excluded.

c) Occurrence of AE is related to the acknowledged characteristics of the drug.

4. Related: (the first 3 items are indispensible) if the following conditions are met after medical judgment, AE is considered to be relevant to the study drug:

a) Obvious temporal relationship exists between drug application and occurrence of AE.

b) Cannot be interpreted by factors like change of disease course, environment or application of other drugs for treatment.

c) AE disappears or relieves with reduced dose or after drug interruption, and recurs after re-administration.

d) Occurrence of AE is relevant to the acknowledged characteristics of the drug.

During SAE report, if the SAE is judged as **possibly related, unlikely related** or **unrelated**, the investigator is required to provide other potential causes leading to the SAE.

7.3 Recording and Reporting of Safety Parameters

7.3.1. AE Recording

During the AE reporting period stipulated in the Protocol, the investigators are responsible for collecting all AEs and record them in the CRF/eCRF. In terms of AE

recording, investigators should use correct and normative medical terminology and avoid spoken language and abbreviations. The content of record should include the start time of AE, the highest degree of NCI CTC AE grading, end time, correlation with the study drug, influence to the study, whether concomitant therapy exist and condition of recovery.

Diagnosis vs Symptoms and Signs

If diagnosis exists, the result should be recorded in the CRF/eCRF rather than single symptoms and signs (e.g.: record liver failure, rather than jaundice, elevation of transaminase and asterixis). However, if the symptoms and signs cannot be categorized as a single diagnosis during reporting period, each single event should be recorded as AE in the CRF/eCRF. On condition that the diagnosis is confirmed afterwards, CRF/eCRF should be updated for diagnosis recording.

AEs Secondary to Other Events

Generally speaking, the primary events should be recorded for AE secondary to other events (such as induced by other events or clinical sequelae), unless the secondary events present more severe intensity or become SAEs. However, the secondary events with obvious clinical significance should be recorded as independent AE in the CRF/eCRF if they have different time of occurrence with the primary events. If the correlation between the events remains unclear, records should be completed individually in the CRF.

Continuous, Intermittent or Single AE (Frequency of AE)

Continuous AE refers to an AE exists continuously through the whole process without remission, for example, a continuous RTI which lasts for 5d. This type of AE should be recorded in the CRF once only. The most intense severity throughout the event should be recorded during intensity evaluation.

Intermittent AE refers to an AE without outcome with obvious clinical significance but presents occasional variation or remission in terms of symptoms, signs or laboratory tests through the whole process, for example, nausea and vomiting continuing for days and alleviates comparatively during the process; subjects with hypertension presents comparatively continuous course of disease during multiple BP

tests despite of intermittent remission. This type of AE should be recorded in CRF for only one time. The most intense severity throughout the event should be recorded during intensity evaluation.

Single AE refers to an AE that can logically only occurs individually, or occurs only once independently during the study, such as an accident of falling down that the subject experiences during the period of drug administration or one occasional vomiting that the subject experiences during the period of test. This type of AE should be recorded in the CRF only once.

It's important to note that if the above-mentioned AE has presented recovery with obvious clinical significance, and the subsequent identical AE is considered to have no consistency in terms of disease course with the previous case, and the occurrence of both events should be recorded respectively in the CRF.

Abnormality of Laboratory Results and Vital Signs

All the results of laboratory tests can be recorded on the page for laboratory results in CRF. Not all the abnormalities of laboratory tests/vital signs are required for AE recording. Investigators are responsible for reviewing all the abnormal laboratory results and vital signs, and determine whether to record them as AE after medical judgment. When any of the above-mentioned abnormalities present obvious clinical significance, or at least one of the following conditions is met, they should be recorded as AEs:

- Accompanied by clinical symptoms.
- Lead to study drug application change (e.g.: dose adjustment, discontinuation or termination)
- Require medical intervention or alteration of concomitant treatment (e.g.: increase, interruption, discontinuation or other change of concomitant medication, treatment or process).
- Having obvious clinical significance according to the investigator.

If the abnormal laboratory results or vital signs with clinical significance are the representations (such as elevation of ALT/AST and hemobilirubin resulted from damage of hepatic function) of a certain disease or syndrome, only the diagnosis

(damage of hepatic function) should be recorded in the AE Record of the CRF/eCRF. Otherwise, the abnormal laboratory results or vital signs should be recorded in the AE Record of the CRF/eCRF and whether the tested value is higher or lower than normal range requires specification (e.g.: record as “serum potassium elevation” rather than “serum potassium abnormality”). If standard clinical terminology corresponding to the abnormalities of laboratory tests or vital signs exists, it is the terminology (e.g. serum potassium elevation up to 7.0mEg/L should be recorded as “hyperkalemia”) that should be recorded in CRF/eCRF.

Death

During record of death event, if AE leading to death exists, single medical concept should be applied to record the AE leading to death in the CRF/eCRF and the event should be regarded as SAE for expedited reporting. If the cause of death remains unknown, “Cause of death unknown” should be recorded in CRF/eCRF and regarded as SAE for expedited reporting. Investigate the exact cause of death further and update the record when the cause of death is confirmed; if death is resulted from tumor progression, it shall not be recorded or reported as AE/SAE.

Pre-existing Medical Conditions

The pre-existing condition of subject during the study screening shall be recorded as AE only when the degree of severity, frequency and nature has worsened (except for deterioration of the investigated disease) after enrollment. Change from the previous condition should be reflected in the record, such as “increased frequency of headache”.

Hospitalization, Prolonged Hospitalization or Surgery

Any AE leading to hospitalization or prolonged hospitalization should be recorded and reported as SAE, except for the following conditions:

- Scheduled hospitalization or prolonged hospitalization as requirement of the Protocol (e.g. for administration and efficacy evaluation, etc.)
- Hospitalization due to pre-existing and unchanged medical condition before participation in the study, such as scheduled selective surgery or treatment before enrollment of the study; however, the required surgery and treatment due to

condition deterioration of the existing disease during the study shall be considered as AE.

Pregnancy

If female subject presents pregnancy during the study, the study drug should be terminated immediately and the investigator should be informed. Investigators should report it to the sponsor within 24 hours, and discuss the risk of pregnancy continuation and potential impact on the fetus with the subject. Monitoring of the subject should be continued to the completion of pregnancy. All pregnancy within 30 days after the final administration of study drug should be reported to the investigator.

Abortion should be recorded and reported as SAE whether it is artificial or spontaneous. Any congenital abnormality/birth defect of the infant born by female subject or female partners of male subject that has used the study drug should be recorded and reported as SAE.

Progressive Disease

If the event is definitely consistent with the anticipated progression pattern of primary tumor, the event should not be regarded as AE. Hospitalization induced by simply progressive disease is not considered as an SAE. If symptoms cannot be confirmed to be completely induced by progressive disease or does not consist with the anticipated progression pattern of tumor, the relevant clinical symptoms can be recorded as AE.

7.3.2. Expedited Reporting of SAEs

If any SAE is discovered by the investigator during the course of study, regardless of whether it is related to the study drug, the finished *SAE Report* should be summited to drug administration department of relevant province, autonomous region and municipality as well as CFDA, the sponsor and Ethics Committee by fax within 24 hours after awareness of the SAE. Investigators should finish and submit the follow-up report within the same time limit after obtaining the follow-up information. When a non-serious adverse event progresses into an SAE, the SAE and relevant follow-up report should also be reported within 24 hours. Regardless of whether related with the study drug, proper treatment is required by all SAEs until the subject

has recovered from the event or the event has become less urgent or the condition has been stable in accordance with the determination of investigators.

To ensure the sponsor's timely and full access to safety data, investigators should fill out the *Clinical Trial SAE Report* provided by the sponsor in addition to the *SAE Report* provided by CFDA for all SAEs and submit to the sponsor by fax or through the EDC system within 24 hours. If the subject is hospitalized for treatment due to SAE, the report of diagnosis and treatment issued by the hospital should be faxed to the sponsor in a timely manner.

7.3.3. Report of Special Events Stipulated by the Protocol

All the special events stipulated by protocol should be reported to sponsor regardless of whether they are SAEs or correlated with the study drug.

- For events conforming to the requirements of SAE, report as the SAE expedited reporting procedures (see Section 7.3.2);
- For events not conforming to the requirements of SAE, please complete the required SAE report and submit it by fax or through the EDC system to the sponsor within 24 hours.

Please refer to Appendix 4 for the procedure of reporting AE/SAE, and Appendix 5 for the asseessment of special events (liver damage) regulated by the Protocol.

7.4 Subject Follow-up

7.4.1. Follow-up of AE

Investigators should follow-up all AEs until occurrence of any of the following conditions:

- AE is relieved or improved to baseline level.
- No further anticipated improvement will present according to the investigator.
- Death of the subject.
- Lost contact with the subject.
- The AE is unrelated to study treatment according to the investigator.
- Subject initiates new anticancer treatment

- End of study.

The final outcome (including AE remission or date of death) of each AE is required to be recorded in the CRF/eCRF.

7.4.2. Follow-up of SAEs

All follow-ups of SAEs should be performed to the greatest extent till SAE is relieved or improved to baseline level, the subject is dead, loss of contact with the subject or that the AE is unrelated to study treatment according to the investigator. The investigator should fill out the follow-up report or summary report in *Report of SAE* with SAE follow-up information; fill out the SAE Report required by the sponsor and report as the SAE expedited reporting procedure (see 7.3.2).

For some SAEs, the sponsor may inquire the investigator for more case information of the event by EDC system, email, fax or monitoring visit, etc.

7.4.3. Follow-up of Special Events Regulated by the Protocol

For all special events regulated by protocol, follow-ups should be performed with reference to the *Clinical Evaluation of Liver Damage* provided in Appendix 5 in addition to regular SAE follow-ups to obtain more effective information for evaluating this type of events.

7.5 Emergency Unblinding

The principal investigator of the site should determine whether to perform emergency unblinding by combining the actual emergency condition and need of rescue once major safety event occur. Unblinding should be performed with IWRS after informing of the clinical head from the sponsor or the monitors of each site when necessary. Once unblinding is performed, treatment for that subject shall be terminated.

8 Statistical Analysis Plan

8.1 Primary and Secondary Endpoints

Primary Endpoint:

Survival or Progression-free Survival: refers to the time interval between the randomized date and the initial record of PD or date of death whichever is earlier. The presence of PD shall be determined in accordance with the result of evaluation performed by the investigator with RECIST v1.1. Set the date of final tumor evaluation as the censored date for patients not yet presented disease progression or death. Set the randomized date as the censored date for patients that did not performed tumor evaluation at post-baseline stage.

Secondary Endpoints:

The secondary efficacy variables include tumor Overall Response Rate (ORR), Disease Control Rate (DCR) and survival.

Tumor ORR: defined as the occurrence rate of confirmed Complete Response (CR) or Partial Response (PR), evaluate target lesions and non-target lesions with corroborant radiological method and determined by RECIST v1.1. Subjects have not performed tumor evaluation at post-baseline stage shall be regarded as patients without remission. Subjects qualified for evaluation of CR or PR should have at least one available lesion for measurement with RECIST v1.1.

DCR: defined as the occurrence rate of corroborant CR, PR and Stable Disease (SD), evaluate target lesions and non-target lesions with corroborant radiological method and determine according to the RECIST v1.1.

Overall Survival (OS): refers to the time interval between the randomized date and the date of death (any cause). Set the final known date of survival as the censored date for subjects that have not been reported to be dead by the time of analysis.

Safety:

The safety of treatment shall be evaluated through AE, laboratory tests, vital signs, ECG, UCG (especially LVEF) and ECOG PS.

All the subjects receiving at least one time of treatment shall be included in the safety evaluation.

8.2 Statistical Analysis Method

8.2.1. Statistical Model

Primary efficacy endpoints: PFS

The comparison of PFS of primary endpoints of the two groups is the stratified log-rank test based upon the ITT population at a two-sided significance level of 0.05. When PFS presents statistical significance under the above-mentioned significance level, the study result can be declared as positive.

The log-rank test result of stratified factors shall be provided as well. The stratified factors include previous chemotherapy (2 vs. ≥3), previous treatment by VEGF inhibitors including Bevacizumab, etc. (yes vs. no) and liver metastasis (yes vs. no). Estimate the MST in two treatment groups by adopting Kaplan-Meier method and provide visually intuitive description to the difference between treatment groups by drawing Kaplan-Meier curve. Estimation of treatment effects shall be expressed by the HR estimated by stratified COX model in a 95% Confidential Interval (CI) while the analysis result of stratified factors shall be provided.

Secondary efficacy endpoints: DCR, ORR and OS

The analysis set of DCR and ORR is the subjects of ITT set with measurable lesions of baseline disease. Apply Blyth-Still-Casella method to calculate the estimated value of DCR and ORR in each treatment group respectively in a 95% CI. Apply stratified Mantel-Haensze to inspect and compare CR in treatment groups. The CI of CR difference in treatment groups shall be calculated by the approximate normal distribution method of binomial distribution. The comparison of OS is the stratified analysis based upon all ITT population; evaluation of treatment efficacy shall be expressed by the HR estimated by stratified COX model in a 95% CI. Stratified factors are the same with that of PFS. OS analysis shall apply Kaplan-Meier method as well to estimate the MST in two treatment groups, and provide visually intuitive description to the difference between treatment groups by drawing Kaplan-Meier curve.

8.2.2. Types of Analysis

8.2.2.1 Efficacy Analysis

Intention to Treat (ITT): Analyze by the groups allocated in by randomization

according to the principle of intention-to-treat, including all randomized patients.

Safety Analysis Set (SAS): All randomized patients that received treatment by the study drug for at least once should be included in the SAS.

8.2.2.2 Safety Data Analysis

Safety population

Including patients received treatment for at least once after signing the Informed Consent Form (ICF).

All safety parameters shall be summarized and listed in accordance with safety population.

Frequency table (overall and intensity classification) shall be listed in accordance with classification of human body systems for AE data. In the list of overall AE occurrence rate, subjects presented the same AE for more than once shall be calculated once only in the frequency table.

Summarization of laboratory data shall adopt statement changes and frequency table at the same time by each time point of sampling.

All AEs and abnormal laboratory variables shall be evaluated by the NCI CTC AE Version 4.0 Classification System.

Descriptive statistics shall be used for ECOG PS summarization. Vital signs, ECG and UCG shall be described in list.

8.2.2.3 Interim Analysis

No interim analysis is planned.

8.3 Statistical Analysis Plan

See Statistical Analysis Plan (SAP) for details.

The analyzing time of primary endpoints is 6 months after enrollment of all subjects, and OS analysis is performed at the end of the study when all PFS events or 80% OS events are obtained.

8.4 Sample Size

The number of primary endpoint (PFS events) required for the evaluation of efficacy is calculated based on the following assumptions:

- A two-sided significance level of 0.05;
- A 67% test power will be ensured when the true Hazard Ratio (HR) of treatment group/control group is 0.5, in other words, the median PFS time is extended from 2 months to 4 months;
- The enrollment rate is 10 subjects per month and the overall enrollment can be completed in 7 months;
- A subject withdrawl rate of 15%;
- Under the premise of these assumptions, approximately 70 subjects will be enrolled in this study. 6 months after the end of the enrollment, the subjects will be unblinded for the final analysis of primary endpoint.

9 Data Quality Assurance

Electronic data management system will be adopted in this study.

Establishment of eCRF: eCRF shall be established by data administrators according to the Protocol.

Access permission: Data administrators shall create account respectively for different identities including investigator, sponsor, monitor and auditor, etc. and grant different access permission, e.g. investigator of each site can access the content of their own site and are granted with permission of data modification. The sponsor is limited to browse the condition of all cases only; and monitors and auditors can review the case conditions of all sites with permission of inserting comments and raise queries but has no right of data modification.

Data entry: The clinical investigator or data entry personnel (clinical coordinator) designated by the investigator shall input the data in study medical record to eCRF with timely and accurate manner. eCRF shall not be used as original record and the content is originally from "study medical record".

Data query and answers: the monitor can propose queries online when problems are found during the monitoring. And investigators shall answer the queries online and correct data errors. The monitors are allowed to propose repetitive queries under necessary conditions.

Data locking and exporting: The data administrator shall perform data locking after accuracy confirmation of monitors when each subject has completed the study, until completion of data locking for the last subject. When all data is locked, it shall be imported to designated database by data administrator and submitted to statistical staff for statistical analysis.

When the study is completed, eCRF and all queries shall be archived in burned discs as necessary. The data management center shall reserve the electronic data till 5 years after launching of the drug and during which, the data management center can unlock the system at any time after appointment of CFDA for inspection.

10 Data Monitoring Committee (DMC)

A Data Monitoring Committee (DMC) shall be established in this study. It shall be composed by PI, and the medical monitor and safety data monitor from the sponsor.

DMC shall review the safety data on a regular basis. Safety data include: demographic data, AEs, SAEs and laboratory abnormalities.

After data monitoring, DMC shall provide suggestions on whether to continue the study, modify the protocol as required or stop the study. The final decision shall be made by the sponsor.

11 Ethics

11.1 Local Regulations/Declaration of Helsinki

The investigators shall guarantee that the study will be conducted in full compliance with the principle of “Declaration of Helsinki” as well as the local laws and regulations, and make every effort to protect the subjects. The study must keep full compliance with the principle of “Criterions for the Quality Control of Clinical Trial of Drugs” (since Jan. 1997) in ICH three-way guideline or local laws and provide protection to a larger extent for the subjects.

11.2 Informed Consent

The responsibility of investigators or the personnel designated by investigators (if permitted by local law) is to obtain the written ICF from the subjects participating in the study after adequate explanation of study objective, methods, expected benefits and potential risks. For patients that unqualified or unable to provide legal consent, written ICF must be obtained from his/her legal guardian. If the patient and his/her legal guardian cannot read, a notary public must be on the spot during the whole process of informed consent. With the verbal consent of study participation from the patient and his/her legal guardian, the notary public shall sign the ICF to demonstrate the accurate explanation and full understanding of the information in it. Investigators and other designated personnel are also required to make the subjects understand that

they can refuse participating in or withdraw from the study for whatever reason at any time. The CRF of this study contains a certain part for recording the patients' informed consent and it should be completed appropriately. If new safety information induces significant change of risk/benefit evaluation, the ICF shall be updated when necessary. All patients (including patients receiving treatment) should be informed with this new information and provided with a modified ICF. Their consent of continuous participation in the study should be obtained.

11.3 Independent Ethics Committee (IEC)/Institution Review Board (IRB)

The study protocol, other related materials (such as Address to the Patients or study description), advertisements and compensations provided to the patient shall be submitted to the Ethics Committee by the investigator. EC approval must be obtained before starting the study and recorded in form of letters to the investigators, including dates of EC meeting and approval.

Any modification to the study protocol after receiving the EC approval letter shall be submitted to the Ethics Committee by the investigator according to related procedures as well as laws and regulations.

12 Protocol Modification

The study protocol and procedure shall not be changed without consent from both the investigator and the sponsor.

If the study protocol requires modification, the modified content or updated version (modified version) of the protocol shall be submitted to the Ethics Committee for written approval before implementation. Besides, the modified protocol shall be submitted to the local drug administration department or obtain the approval when required and requirements of local departments must be conformed to.

If modification of site ICF is required during protocol modification, the sponsor and the site Ethics Committee must be informed. The modified ICF must be approved by

the Ethics Committee in written form before application.

The sponsor shall distribute the protocol amendment and the modified protocol to every principal investigator and the principal investigators shall be responsible for distributing the documents to the corresponding Ethics Committee and other study personnel.

13 Conditions of Study Termination

Both the sponsor and the investigator can stop the study at any time. If study termination is a must, it shall be stopped after review and negotiation of both parties. When stopping the study, the sponsor and investigators shall make every effort to protect the interests of the subjects.

14 Study Documents, CRFs and Records Retention

14.1 Investigators Files/Records Retention

The investigator should preserve all the study materials for at least 5 years, including the materials of confirmation to all the subjects (for effective verification of different recording materials, such as CRF/eCRF and original hospital records), original ICF of all subjects, CRF/eCRF and detailed drug distribution records, etc.

All the materials of this clinical study are the property of the sponsor, and any provision to a third party in any form or publication of the study data is prohibited without prior authorization of the sponsor.

14.2 Original Documents and Background Materials

Original documents can demonstrate the existence of the patients and verify the completeness of collected data. The original documents shall be archived at the site.

The data transferred to the CRF/eCRF from the original documents must be consistent with the original documents and explanation is required for any differences. In accordance with condition of the study, investigators may require the previous medical records, hospital transfer records or the current medical records.

All data in CRF/eCRF must be obtained from the original documents.

14.3 Direct Access to the Original Data and Documents

The investigators/sites shall accept study-related monitoring, audits, IRB/IEC review and inspections by the regulatory departments, and provide direct access to all related original data/documents. CRF/eCRF and all the original documents including disease condition record and copies of laboratory tests and medical examination results shall be provided for examinations by the clinical research associates (CRA) and auditors of the sponsor as well as the health administrative departments at any time.

15 Study Monitoring

Before the enrollment of the first subject, CRA designated by the sponsor are required to be on the site and:

- Ensure the completeness of facilities and equipment;
- Discuss the responsibilities of study team members and monitors during the study with investigators (or other members) and sign the related agreements;
- Pay regular visits to the site and keep close connection with the investigator during the study
- Provide adequate information and support to the investigator
- Ensure that the study facilities and equipment are still complete
- Ensure the study team keeps full compliance with the Protocol and records data to CRF/eCRF and drug accountability sheet with accuracy;
- Ensure completeness of original data review (check the consistency of CRF/eCRF data and hospital medical record with other study-related records). The access to the original record of each subject is necessary.

The monitors and other representatives of the sponsor shall visit the site as well if the investigator requires more information and suggestions.

16 Confidentiality of the Study Documents and Patients' Records

The investigator is required to ensure anonymity maintenance for patients and prevent patient identity disclosure to unauthorized parties. On the CRF/eCRF or other documents submitted to the sponsor, the patients shall be identified only by codes but not names.

The investigator should keep a record of patient enrollment registration with revealed patient code, name and address. Investigators should keep some certain documents including patient ICF under restrict preservation; submission to Hutchison MediPharma Ltd. is prohibited.

17 Data Publication and Business Confidentiality Protection

The study result may be published or released on scientific meetings. Investigators shall agree to submit all manuscripts or abstracts to Hutchison MediPharma Ltd. in advance before scheduled submission, if applicable. Thus the patent information of the sponsor can be protected and due to the possibility that the investigator may not know information about other studies, the sponsor can also propose suggestions.

In accordance with standard of publication and ethical specifications, Hutchison MediPharma Ltd. supports generally the publication of multi-centered but rather single-centered studies. Under such circumstances, a coordinating investigator shall be designated after agreement of both parties.

18 References

1. Clinical Practice Guidelines for Colorectal Cancers, National Comprehensive Cancer Network (NCCN), Version 2012.
2. Hurwitz H, Fehrenbacher L, Novotny W, et al: Bevacizumab plus irinotecan, fluorouracil, and leucovorin for metastatic colorectal cancer. N Engl J Med 350:2335-2342, 2004
3. Demetri GD, Desai J, Fletcher JA, et al: SU11248, a multi-targeted tyrosine kinase inhibitor, can overcome imatinib (IM) resistance caused by diverse

genomic mechanisms in patients (pts) with metastatic gastrointestinal stromal tumor (GIST). J Clin Oncol 22:3001, 2004

4. Axel Grothey, Alberto F. Sobrero, Salvatore Siena,et al: Results of a phase III randomized, double-blind, placebo-controlled, multicenter trial (CORRECT) of regorafenib plus best supportive care (BSC) versus placebo plus BSC in patients (pts) with metastatic colorectal cancer (mCRC) who have progressed after standard therapies. J Clin Oncol 30, 2012 (suppl 4; abstr LBA385)
5. 郝希山。2011 年第十三届中国科协年会分会“癌症流行趋势和防控的策略研讨会”

Appendix 1 ECOG Performance Status

Eastern Cooperative Oncology Group Performance Status Assessments

ECOG PERFORMANCE STATUS*

Grade	ECOG
0	Fully active, able to carry on all pre-disease performance without restriction
1	Restricted in physically strenuous activity but ambulatory and able to carry out work of a light or sedentary nature, e.g., light house work, office work
2	Ambulatory and capable of all selfcare but unable to carry out any work activities, up and about more than 50% of waking hours
3	Capable of only limited selfcare, confined to bed or chair more than 50% of waking hours
4	Completed disabled; cannot carry on any selfcare; totally confined to bed or chair
5	Dead

Appendix 2. Response Evaluation Criteria in Solid Tumors RECIST Version 1.1

(Response Evaluation Criteria in Solid Tumors RECIST Version 1.1)

Since no formal Chinese version of RICIST can be found at present, the inhouse translated version is adopted. For more detailed information, please see the English Version (European Journal of Cancer 45 (2009) 228-247).

ABSTRACT

Background

Assessment of the change in tumor burden is an important feature of the clinical evaluation of cancer therapeutics: both tumor shrinkage (objective response) and disease progression are useful endpoints in clinical trials. Since RECIST was published in 2000, many investigators, cooperative groups, industry and government authorities have adopted these criteria in the assessment of treatment outcomes. However, a number of questions and issues have arisen which have led to the development of a revised RECIST guideline (version 1.1). Evidence for changes, summarised in separate papers in this special issue, has come from assessment of a large data warehouse (>6500 patients), simulation studies and literature reviews.

Highlights of revised RECIST 1.1:

Major changes include:

Number of lesions to be assessed: based on evidence from numerous trial databases merged into a data warehouse for analysis purposes, the number of lesions required to assess tumor burden for response determination has been reduced from a maximum of 10 to a maximum of five total (and from five to two per organ, maximum).

Assessment of pathological lymph nodes is now incorporated: nodes with a short axis of ≥15mm are considered measurable and assessable as target lesions. The short axis measurement should be included in the sum of lesions in calculation of tumor response. Nodes that shrink to <10 mm short axis are considered normal.

Confirmation of response is required for trials with response primary endpoint but is

no longer required in randomized studies since the control arm serves as appropriate means of interpretation of data. Disease progression is clarified in several aspects: in addition to the previous definition of progression in target disease of 20% increase in sum, a 5 mm absolute increase is now required as well to guard against over calling PD when the total sum is very small. Furthermore, there is guidance offered on what constitutes ‘unequivocal progression’ of non-measurable/non-target disease, a source of confusion in the original RECIST guideline. Finally, a section on detection of new lesions, including the interpretation of FDG-PET scan assessment is included. Imaging guidance: the revised RECIST includes a new imaging appendix with updated recommendations on the optimal anatomical assessment of lesions.

Future work:

A key question considered by the RECIST Working Group in developing RECIST 1.1 was whether it was appropriate to move from anatomic unidimensional assessment of tumor burden to either volumetric anatomical assessment or to functional assessment with PET or MRI. It was concluded that, at present, there is not sufficient standardization or evidence to abandon anatomical assessment of tumor burden. The only exception to this is in the use of FDG-PET imaging as an adjunct to determination of progression. As is detailed in the final paper in this special issue, the use of these promising newer approaches requires appropriate clinical validation studies.

Keywords: Response criteria, Solid tumors, Guidelines

1. Background

1.1. History of RECIST Criteria

Assessment of the change in tumor burden is an important feature of the clinical evaluation of cancer therapeutics. Both tumor shrinkage (objective response) and time to the development of disease progression are important endpoints in cancer clinical trials. The use of tumor regression as the endpoint for phase II trials screening new agents for evidence of anti-tumor effect is supported by years of evidence suggesting that, for many solid tumors, agents which produce tumor shrinkage in a proportion of patients have a reasonable (albeit imperfect) chance of subsequently demonstrating an

improvement in overall survival or other time to event measures in randomized phase III studies. At the current time objective response carries with it a body of evidence greater than for any other biomarker supporting its utility as a measure of promising treatment effect in phase II screening trials. Furthermore, at both the phase II and phase III stage of drug development, clinical trials in advanced disease settings are increasingly utilizing time to progression (or progression-free survival) as an endpoint upon which efficacy conclusions are drawn, which is also based on anatomical measurement of tumor size.

However, both of these tumor endpoints, objective response and time to disease progression, are useful only if based on widely accepted and readily applied standard criteria based on anatomical tumor burden. In 1981 the World Health Organization (WHO) first published tumor response criteria, mainly for use in trials where tumor response was the primary endpoint. The WHO criteria introduced the concept of an overall assessment of tumor burden by summing the products of bidimensional lesion measurements and determined response to therapy by evaluation of change from baseline while on treatment. However, in the decades that followed their publication, cooperative groups and pharmaceutical companies that used the WHO criteria often ‘modified’ them to accommodate new technologies or to address areas that were unclear in the original document. This led to confusion in interpretation of trial results and in fact, the application of varying response criteria was shown to lead to very different conclusions about the efficacy of the same regimen. In response to these problems, an International Working Party was formed in the mid 1990s to standardize and simplify response criteria.

New criteria, known as RECIST (Response Evaluation Criteria in Solid Tumors), were published in 2000. Key features of the original RECIST include definitions of minimum size of measurable lesions, instructions on how many lesions to follow (up to 10; a maximum five per organ site), and the use of unidimensional, rather than bidimensional, measures for overall evaluation of tumor burden. These criteria have subsequently been widely adopted by academic institutions, cooperative groups, and industry for trials where the primary endpoints are objective response or progression.

In addition, regulatory authorities accept RECIST as an appropriate guideline for these assessments.

2. Purpose of this Guideline

This guideline describes a standard approach to solid tumor measurement and definitions for objective assessment of change in tumor size for use in adult and paediatric cancer clinical trials. It is expected these criteria will be useful in all trials where objective response is the primary study endpoint, as well as in trials where assessment of stable disease, tumor progression or time to progression analyses are undertaken, since all of these outcome measures are based on an assessment of anatomical tumor burden and its change on study. There are no assumptions in this paper about the proportion of patients meeting the criteria for any of these endpoints which will signal that an agent or treatment regimen is active: those definitions are dependent on type of cancer in which a trial is being undertaken and the specific agent(s) under study. Protocols must include appropriate statistical sections which define the efficacy parameters upon which the trial sample size and decision criteria are based. In addition to providing definitions and criteria for assessment of tumor response, this guideline also makes recommendations regarding standard reporting of the results of trials that utilize tumor response as an endpoint.

While these guidelines may be applied in malignant brain tumor studies, there are also separate criteria published for response assessment in that setting. This guideline is not intended for use for studies of malignant lymphoma since international guidelines for response assessment in lymphoma are published separately.

Finally, many oncologists in their daily clinical practice follow their patients' malignant disease by means of repeated imaging studies and make decisions about continued therapy on the basis of both objective and symptomatic criteria. It is not intended that these RECIST guidelines play a role in that decision making, except if determined appropriate by the treating oncologist.

3. Measurability of Tumor at Baseline

3.1. Definitions

At baseline, tumor lesions/lymph nodes will be categorized measurable or

non-measurable as follows:

3.1.1. Measurable

Tumor lesions: Must be accurately measured in at least one dimension (longest diameter in the plane of measurement is to be recorded) with a minimum size of:

- 10mm by CT scan (CT scan slice thickness no greater than 5 mm).
- 10mm caliper measurement by clinical exam (lesions which cannot be accurately measured with calipers should be recorded as non-measurable).
- 20mm by chest X-ray.

Malignant lymph nodes: To be considered pathologically enlarged and measurable, a lymph node must be ≥15mm in short axis when assessed by CT scan (CT scan slice thickness recommended to be no greater than 5 mm). At baseline and in follow-up, only the short axis will be measured and followed. See also notes below on 'Baseline documentation of target and non-target lesions' for information on lymph node measurement.

3.1.2. Non-measurable

All other lesions, including small lesions (longest diameter <10mm or pathological lymph nodes with ≥10 to <15mm short axis) as well as truly non-measurable lesions. Lesions considered truly non-measurable include: leptomeningeal disease, ascites, pleural or pericardial effusion, inflammatory breast disease, lymphangitic involvement of skin or lung, abdominal masses/abdominal organomegaly identified by physical exam that is not measurable by reproducible imaging techniques.

3.1.3. Special Considerations Regarding Lesion Measurability

Bone lesions, cystic lesions, and lesions previously treated with local therapy require particular comment:

Bone lesions:

- Bone scan, PET scan or plain films are not considered adequate imaging techniques to measure bone lesions. However, these techniques can be used to confirm the presence or disappearance of bone lesions.

- Lytic bone lesions or mixed lytic-blastic lesions, with identifiable soft tissue components, that can be evaluated by cross sectional imaging techniques such as CT or MRI can be considered as measurable lesions if the soft tissue component meets the definition of measurability described above.
- Blastic bone lesions are non-measurable.

Cystic lesions:

- Lesions that meet the criteria for radiographically defined simple cysts should not be considered as malignant lesions (neither measurable nor non-measurable) since they are, by definition, simple cysts.
- ‘Cystic lesions’ thought to represent cystic metastases can be considered as measurable lesions, if they meet the definition of measurability described above. However, if noncystic lesions are present in the same patient, these are preferred for selection as target lesions.

  Lesions with prior local treatment:
- Tumor lesions situated in a previously irradiated area, or in an area subjected to other loco-regional therapy, are usually not considered measurable unless there has been demonstrated progression in the lesion. Study protocols should detail the conditions under which such lesions would be considered measurable.

3.2. Specifications by Methods of Measurements

3.2.1. Measurement of Lesions

All measurements should be recorded in metric notation, using calipers if clinically assessed. All baseline evaluations should be performed as close as possible to the treatment start and never more than 4 weeks before the beginning of the treatment.

3.2.2. Method of Assessment

The same method of assessment and the same technique should be used to characterize each identified and reported lesion at baseline and during follow-up. Imaging based evaluation should always be done rather than clinical examination unless the lesion(s) being followed cannot be imaged but are assessable by clinical exam.

Clinical lesions: Clinical lesions will only be considered measurable when they are

superficial and P10mm diameter as assessed using calipers (e.g. skin nodules). For the case of skin lesions, documentation by color photography including a ruler to estimate the size of the lesion is suggested. As noted above, when lesions can be evaluated by both clinical exam and imaging, imaging evaluation should be undertaken since it is more objective and may also be reviewed at the end of the study.

Chest X-ray: Chest CT is preferred over chest X-ray, particularly when progression is an important endpoint, since CT is more sensitive than X-ray, particularly in identifying new lesions. However, lesions on chest X-ray may be considered measurable if they are clearly defined and surrounded by aerated lung.

CT, MRI: CT is the best currently available and reproducible method to measure lesions selected for response assessment. This guideline has defined measurability of lesions on CT scan based on the assumption that CT slice thickness is 5mm or less. As is described in Appendix II, when CT scans have slice thickness greater than 5 mm, the minimum size for a measurable lesion should be twice the slice thickness. MRI is also acceptable in certain situations (e.g. for body scans). More details concerning the use of both CT and MRI for assessment of objective tumor response evaluation are provided in Appendix II.

Ultrasound: Ultrasound is not useful in assessment of lesion size and should not be used as a method of measurement.

Ultrasound examinations cannot be reproduced in their entirety for independent review at a later date and, because they are operator dependent, it cannot be guaranteed that the same technique and measurements will be taken from one assessment to the next (described in greater detail in Appendix II). If new lesions are identified by ultrasound in the course of the study, confirmation by CT or MRI is advised. If there is concern about radiation exposure at CT, MRI may be used instead of CT in selected instances.

Endoscopy, laparoscopy: The utilization of these techniques for objective tumor evaluation is not advised. However, they can be useful to confirm complete pathological response when biopsies are obtained or to determine relapse in trials where recurrence following complete response or surgical resection is an endpoint.

Tumor markers: Tumor markers alone cannot be used to assess objective tumor response. If markers are initially above the upper normal limit, however, they must normalize for a patient to be considered in complete response. Because tumor markers are disease specific, instructions for their measurement should be incorporated into protocols on a disease specific basis. Specific guidelines for both CA-125 response (in recurrent ovarian cancer) and PSA response (in recurrent prostate cancer), have been published. In addition, the Gynecologic Cancer Intergroup has developed CA125 progression criteria which are to be integrated with objective tumor assessment for use in first-line trials in ovarian cancer.

Cytology, histology: These techniques can be used to differentiate between PR and CR in rare cases if required by protocol (for example, residual lesions in tumor types such as germ cell tumors, where known residual benign tumors can remain). When effusions are known to be a potential adverse effect of treatment (e.g. with certain taxane compounds or angiogenesis inhibitors), the cytological confirmation of the neoplastic origin of any effusion that appears or worsens during treatment can be considered if the measurable tumor has met criteria for response or stable disease in order to differentiate between response (or stable disease) and progressive disease.

4. Tumor Response Evaluation

4.1. Assessment of Overall Tumor Burden and Measurable Disease

To assess objective response or future progression, it is necessary to estimate the overall tumor burden at baseline and use this as a comparator for subsequent measurements. Only patients with measurable disease at baseline should be included in protocols where objective tumor response is the primary endpoint. Measurable disease is defined by the presence of at least one measurable lesion. In studies where the primary endpoint is tumor progression (either time to progression or proportion with progression at a fixed date), the protocol must specify if entry is restricted to those with measurable disease or whether patients having non-measurable disease only are also eligible.

4.2. Baseline Documentation of 'Target' and 'Non-target' Lesions

When more than one measurable lesion is present at baseline all lesions up to a

maximum of five lesions total (and a maximum of two lesions per organ) representative of all involved organs should be identified as target lesions and will be recorded and measured at baseline (this means in instances where patients have only one or two organ sites involved a maximum of two and four lesions respectively will be recorded).

Target lesions should be selected on the basis of their size (lesions with the longest diameter), be representative of all involved organs, but in addition should be those that lend themselves to reproducible repeated measurements. It may be the case that, on occasion, the largest lesion does not lend itself to reproducible measurement in which circumstance the next largest lesion which can be measured reproducibly should be selected.

Lymph nodes merit special mention since they are normal anatomical structures which may be visible by imaging even if not involved by tumor. Pathological nodes which are defined as measurable and may be identified as target lesions must meet the criterion of a short axis of ≥15mm by CT scan. Only the short axis of these nodes will contribute to the baseline sum. The short axis of the node is the diameter normally used by radiologists to judge if a node is involved by solid tumor. Nodal size is normally reported as two dimensions in the plane in which the image is obtained (for CT scan this is almost always the axial plane; for MRI the plane of acquisition may be axial, saggital or coronal). The smaller of these measures is the short axis. For example, an abdominal node which is reported as being 20mm × 30mm has a short axis of 20mm and qualifies as a malignant, measurable node. In this example, 20mm should be recorded as the node measurement. All other pathological nodes (those with short axis ≥10mm but <15 mm) should be considered non-target lesions. Nodes that have a short axis <10mm are considered non-pathological and should not be recorded or followed.

A sum of the diameters (longest for non-nodal lesions, short axis for nodal lesions) for all target lesions will be calculated and reported as the baseline sum diameters. If lymph nodes are to be included in the sum, then as noted above, only the short axis is

added into the sum. The baseline sum diameters will be used as reference to further characterize any objective tumor regression in the measurable dimension of the disease.

All other lesions (or sites of disease) including pathological lymph nodes should be identified as non-target lesions and should also be recorded at baseline. Measurements are not required and these lesions should be followed as ‘present’, ‘absent’, or in rare cases ‘unequivocal progression’. In addition, it is possible to record multiple non-target lesions involving the same organ as a single item on the case record form (e.g. ‘multiple enlarged pelvic lymph nodes’ or ‘multiple liver metastases’).

4.3. Response Criteria

This section provides the definitions of the criteria used to determine objective tumor response for target lesions.

4.3.1. Evaluation of Target Lesions

Complete Response (CR): Disappearance of all target lesions.

Any pathological lymph nodes (whether target or non-target) must have reduction in short axis to <10 mm.

Partial Response (PR): At least a 30% decrease in the sum of diameters of target lesions, taking as reference the baseline sum diameters

Progressive Disease (PD): At least a 20% increase in the sum of diameters of target lesions, taking as reference the smallest sum on study (this includes the baseline sum if that is the smallest on study). In addition to the relative increase of 20%, the sum must also demonstrate an absolute increase of at least 5 mm. (Note: the appearance of one or more new lesions is also considered progression).

Stable Disease (SD): Neither sufficient shrinkage to qualify for PR nor sufficient increase to qualify for PD, taking as reference the smallest sum diameters while on study.

4.3.2. Special Notes on the Assessment of Target Lesions

Lymph nodes:

Lymph nodes identified as target lesions should always have the actual short axis measurement recorded (measured in the same anatomical plane as the baseline

examination), even if the nodes regress to below 10mm on study. This means that when lymph nodes are included as target lesions, the 'sum' of lesions may not be zero even if complete response criteria are met, since a normal lymph node is defined as having a short axis of <10mm. Case report forms or other data collection methods may therefore be designed to have target nodal lesions recorded in a separate section where, in order to qualify for CR, each node must achieve a short axis <10mm. For PR, SD and PD, the actual short axis measurement of the nodes is to be included in the sum of target lesions.

Target Lesions that Become 'too Small to Measure'：

While on study, all lesions (nodal and non-nodal) recorded at baseline should have their actual measurements recorded at each subsequent evaluation, even when very small (e.g. 2mm).

However, sometimes lesions or lymph nodes which are recorded as target lesions at baseline become so faint on CT scan that the radiologist may not feel comfortable assigning an exact measure and may report them as being 'too small to measure'. When this occurs it is important that a value be recorded on the case report form. If it is the opinion of the radiologist that the lesion has likely disappeared, the measurement should be recorded as 0mm. If the lesion is believed to be present and is faintly seen but too small to measure, a default value of 5mm should be assigned (Note: It is less likely that this rule will be used for lymph nodes since they usually have a definable size when normal and are frequently surrounded by fat such as in the retroperitoneum; however, if a lymph node is believed to be present and is faintly seen but too small to measure, a default value of 5mm should be assigned in this circumstance as well).

This default value is derived from the 5mm CT slice thickness (but should not be changed with varying CT slice thickness). The measurement of these lesions is potentially non-reproducible, therefore providing this default value will prevent false responses or progressions based upon measurement error. To reiterate, however, if the radiologist is able to provide an actual measure, that should be recorded, even if it is

below 5mm.

Lesions that Split or Coalesce on Treatment:

As noted in Appendix II, when non-nodal lesions ‘fragment’, the longest diameters of the fragmented portions should be added together to calculate the target lesion sum. Similarly, as lesions coalesce, a plane between them may be maintained that would aid in obtaining maximal diameter measurements of each individual lesion. If the lesions have truly coalesced such that they are no longer separable, the vector of the longest diameter in this instance should be the maximal longest diameter for the ‘coalesced lesion’.

4.3.3. Evaluation of Non-target Lesions

This section provides the definitions of the criteria used to determine the tumor response for the group of non-target lesions. While some non-target lesions may actually be measurable, they need not be measured and instead should be assessed only qualitatively at the time points specified in the protocol.

Complete Response (CR): Disappearance of all non-target lesions and normalization of tumor marker level. All lymph nodes must be non-pathological in size (<10mm short axis).

Non-CR/Non-PD: Persistence of one or more non-target lesion(s) and/or maintenance of tumor marker level above the normal limits.

Progressive Disease (PD): Unequivocal progression (see comments below) of existing non-target lesions. (Note: the appearance of one or more new lesions is also considered progression).

4.3.4. Special Notes on Assessment of Progression of Non-target Disease

The concept of progression of non-target disease requires additional explanation as follows:

When the patient also has measurable disease. In this setting, to achieve ‘unequivocal progression’ on the basis of the non-target disease, there must be an overall level of substantial worsening in non-target disease such that, even in presence of SD or PR in target disease, the overall tumor burden has increased sufficiently to merit discontinuation of therapy. A modest ‘increase’ in the size of one or more non-target

lesions is usually not sufficient to quality for unequivocal progression status. The designation of overall progression solely on the basis of change in non-target disease in the face of SD or PR of target disease will therefore be extremely rare.

When the patient has only non-measurable disease. This circumstance arises in some phase III trials when it is not a criterion of study entry to have measurable disease. The same general concepts apply here as noted above, however, in this instance there is no measurable disease assessment to factor into the interpretation of an increase in non-measurable disease burden. Because worsening in non-target disease cannot be easily quantified (by definition: if all lesions are truly non-measurable) a useful test that can be applied when assessing patients for unequivocal progression is to consider if the increase in overall disease burden based on the change in non-measurable disease is comparable in magnitude to the increase that would be required to declare PD for measurable disease: i.e. an increase in tumor burden representing an additional 73% increase in 'volume' (which is equivalent to a 20% increase diameter in a measurable lesion). Examples include an increase in a pleural effusion from 'trace' to 'large', an increase in lymphangitic disease from localized to widespread, or may be described in protocols as 'sufficient to require a change in therapy'. If 'unequivocal progression' is seen, the patient should be considered to have had overall PD at that point. While it would be ideal to have objective criteria to apply to non-measurable disease, the very nature of that disease makes it impossible to do so, therefore the increase must be substantial.

4.3.5. New Lesions

The appearance of new malignant lesions denotes disease progression; therefore, some comments on detection of new lesions are important. There are no specific criteria for the identification of new radiographic lesions; however, the finding of a new lesion should be unequivocal: i.e. not attributable to differences in scanning technique, change in imaging modality or findings thought to represent something other than tumor (for example, some 'new' bone lesions may be simply healing or flare of pre-existing lesions). This is particularly important when the patient's baseline lesions show partial or complete response. For example, necrosis of a liver lesion may

be reported on a CT scan report as a 'new' cystic lesion, which it is not.

A lesion identified on a follow-up study in an anatomical location that was not scanned at baseline is considered a new lesion and will indicate disease progression. An example of this is the patient who has visceral disease at baseline and while on study has a CT or MRI brain ordered which reveals metastases. The patient's brain metastases are considered to be evidence of PD even if he/she did not have brain imaging at baseline.

If a new lesion is equivocal, for example because of its small size, continued therapy and follow-up evaluation will clarify if it represents truly new disease. If repeat scans confirm there is definitely a new lesion, then progression should be declared using the date of the initial scan.

While FDG-PET response assessments need additional study, it is sometimes reasonable to incorporate the use of FDG-PET scanning to complement CT scanning in assessment of progression (particularly possible 'new' disease). New lesions on the basis of FDG-PET imaging can be identified according to the following algorithm:

a. Negative FDG-PET at baseline, with a positive FDG-PET at follow-up is a sign of PD based on a new lesion.

b. No FDG-PET at baseline and a positive FDG-PET at follow-up:

c. If the positive FDG-PET at follow-up corresponds to a new site of disease confirmed by CT, this is PD.

d. If the positive FDG-PET at follow-up is not confirmed as a new site of disease on CT, additional follow-up CT scans are needed to determine if there is truly progression occurring at that site (if so, the date of PD will be the date of the initial abnormal FDG-PET scan).

e. If the positive FDG-PET at follow-up corresponds to a pre-existing site of disease on CT that is not progressing on the basis of the anatomic images, this is not PD.

4.4. Evaluation of Best Overall Response

The best overall response is the best response recorded from the start of the study treatment until the end of treatment taking into account any requirement for confirmation. On occasion a response may not be documented until after the end of

therapy so protocols should be clear if post-treatment assessments are to be considered in determination of best overall response. Protocols must specify how any new therapy introduced before progression will affect best response designation. The patient's best overall response assignment will depend on the findings of both target and non-target disease and will also take into consideration the appearance of new lesions. Furthermore, depending on the nature of the study and the protocol requirements, it may also require confirmatory measurement (see Section 4.6). Specifically, in non-randomized trials where response is the primary endpoint, confirmation of PR or CR is needed to deem either one the 'best overall response'. This is described further below.

4.4.1. Time Point Response

It is assumed that at each protocol specified time point, a response assessment occurs. Table 1 on the next page provides a summary of the overall response status calculation at each time point for patients who have measurable disease at baseline.

When patients have non-measurable (therefore non-target) disease only, Table 2 is to be used.

4.4.2. Missing Assessments and Inevaluable Designation

When no imaging/measurement is done at all at a particular time point, the patient is not evaluable (NE) at that time point.

If only a subset of lesion measurements are made at an assessment, usually the case is also considered NE at that time point, unless a convincing argument can be made that the contribution of the individual missing lesion(s) would not change the assigned time point response. This would be most likely to happen in the case of PD. For example, if a patient had a baseline sum of 50mm with three measured lesions and at follow-up only two lesions were assessed, but those gave a sum of 80 mm, the patient will have achieved PD status, regardless of the contribution of the missing lesion.

4.4.3. Best Overall Response: All Time Points

The best overall response is determined once all the data for the patient is known.

Best response determination in trials where confirmation of complete or partial response IS NOT required: Best response in these trials is defined as the best response

across all time points (for example, a patient who has SD at first assessment, PR at second assessment, and PD on last assessment has a best overall response of PR). When SD is believed to be best response, it must also meet the protocol specified minimum time from baseline. If the minimum time is not met when SD is otherwise the best time point response, the patient’s best response depends on the subsequent assessments. For example, a patient who has SD at first assessment, PD at second and does not meet minimum duration for SD, will have a best response of PD. The same patient lost to follow-up after the first SD assessment would be considered inevaluable.

Best response determination in trials where confirmation of complete or partial response IS required: Complete or partial responses may be claimed only if the criteria for each are met at a subsequent time point as specified in the protocol (generally 4 weeks later). In this circumstance, the best overall response can be interpreted as in Table 3.

4.4.4. Special Notes on Response Assessment

When nodal disease is included in the sum of target lesions and the nodes decrease to ‘normal’ size (<10 mm), they may still have a measurement reported on scans. This measurement should be recorded even though the nodes are normal in order not to overstate progression should it be based on increase in size of the nodes. As noted earlier, this means that patients with CR may not have a total sum of ‘zero’ on the case report form (CRF).

In trials where confirmation of response is required, repeated ‘NE’ time point assessments may complicate best response determination. The analysis plan for the trial must address how missing data/assessments will be addressed in determination of response and progression. For example, in most trials it is reasonable to consider a patient with time point responses of PR-NE-PR as a confirmed response.

Patients with a global deterioration of health status requiring discontinuation of treatment without objective evidence of disease progression at that time should be reported as ‘symptomatic deterioration’. Every effort should be made to document objective progression even after discontinuation of treatment. Symptomatic

deterioration is not a descriptor of an objective response: it is a reason for stopping study therapy. The objective response status of such patients is to be determined by evaluation of target and non-target disease as shown in Tables 1–3.

Conditions that define ‘early progression, early death and inevaluability’ are study specific and should be clearly described in each protocol (depending on treatment duration, treatment periodicity).

In some circumstances it may be difficult to distinguish residual disease from normal tissue. When the evaluation of complete response depends upon this determination, it is recommended that the residual lesion be investigated (fine needle aspirate/biopsy) before assigning a status of complete response. FDG-PET may be used to upgrade a response to a CR in a manner similar to a biopsy in cases where a residual radiographic abnormality is thought to represent fibrosis or scarring. The use of FDG-PET in this circumstance should be prospectively described in the protocol and supported by disease specific medical literature for the indication. However, it must be acknowledged that both approaches may lead to false positive CR due to limitations of FDG-PET and biopsy resolution/sensitivity.

Table 1 Time Point Response: Patients with Target (+/– Non-target) Disease.

Target lesions	Non-target lesions	New lesions	Overall response
CR	CR	No	CR
CR	Non-CR/non-PD	No	PR
CR	Not evaluated	No	PR
PR	Non-PD or not all evaluated	No	PR
SD	Non-PD or not all evaluated	No	SD
Not all evaluated	Non-PD	No	NE
PD	Any	Yes or No	PD
Any	PD	Yes or No	PD
Any	Any	Yes	PD

CR = complete response, PR = partial response, SD = stable disease, PD = progressive disease, and NE = inevaluable.

Table 2 – Time Point Response: Patients with Non-target Disease Only.

Non-target lesions	New lesions	Overall response
CR	No	CR
Non-CR/non-PD	No	Non-CR/non-PD*
Not all evaluated	No	NE
Unequivocal PD	Yes or No	PD
Any	Yes	PD

Note：'Non-CR/non-PD' is preferred over 'stable disease' for non-target disease since SD is increasingly used as endpoint for assessment of efficacy in some trials so to assign this category when no lesions can be measured is not advised.

For equivocal findings of progression (e.g. very small and uncertain new lesions; cystic changes or necrosis in existing lesions), treatment may continue until the next scheduled assessment. If at the next scheduled assessment, progression is confirmed, the date of progression should be the earlier date when progression was suspected.

Table 3 Best Overall Response when Confirmation of CR and PR Required.

Overall response First time point	Overall response Subsequent time point	BEST overall response
CR	CR	CR
CR	PR	SD, PD or PRa
CR	SD	SD provided minimum criteria for SD duration met, otherwise, PD
CR	PD	SD provided minimum criteria for SD duration met, otherwise, PD
CR	NE	SD provided minimum criteria for SD duration met, otherwise NE

PR	CR	PR
PR	PR	PR
PR	SD	SD
PR	PD	SD provided minimum criteria for SD duration met, otherwise, PD
PR	NE	SD provided minimum criteria for SD duration met, otherwise NE
NE	NE	NE

Note: CR = complete response, PR = partial response, SD = stable disease, PD = progressive disease, and NE = inevaluable.

[a]: If a CR is truly met at first time point, then any disease seen at a subsequent time point, even disease meeting PR criteria relative to baseline, makes the disease PD at that point (since disease must have reappeared after CR). Best response would depend on whether minimum duration for SD was met. However, sometimes 'CR' may be claimed when subsequent scans suggest small lesions were likely still present and in fact the patient had PR, not CR at the first time point. Under these circumstances, the original CR should be changed to PR and the best response is PR.

4.5. Frequency of Tumor Re-evaluation

Frequency of tumor re-evaluation while on treatment should be protocol specific and adapted to the type and schedule of treatment. However, in the context of phase II studies where the beneficial effect of therapy is not known, follow-up every 6–8 weeks (timed to coincide with the end of a cycle) is reasonable. Smaller or greater time intervals than these could be justified in specific regimens or circumstances. The protocol should specify which organ sites are to be evaluated at baseline (usually those most likely to be involved with metastatic disease for the tumor type under study) and how often evaluations are repeated. Normally, all target and non-target sites are evaluated at each assessment. In selected circumstances certain non-target organs may be evaluated less frequently. For example, bone scans may need to be repeated only when complete response is identified in target disease or when

progression in bone is suspected.

After the end of the treatment, the need for repetitive tumor evaluations depends on whether the trial has as a goal the response rate or the time to an event (progression/death). If 'time to an event' (e.g. time to progression, disease-free survival, progression-free survival) is the main endpoint of the study, then routine scheduled re-evaluation of protocol specified sites of disease is warranted. In randomized comparative trials in particular, the scheduled assessments should be performed as identified on a calendar schedule (for example: every 6~8 weeks on treatment or every 3~4 months after treatment) and should not be affected by delays in therapy, drug holidays or any other events that might lead to imbalance in a treatment arm in the timing of disease assessment.

4.6. Confirmatory Measurement/Duration of Response

4.6.1. Confirmation

In non-randomized trials where response is the primary endpoint, confirmation of PR and CR is required to ensure responses identified are not the result of measurement error. This will also permit appropriate interpretation of results in the context of historical data where response has traditionally required confirmation in such trials. However, in all other circumstances, i.e. in randomized trials (phase II or III) or studies where stable disease or progression are the primary endpoints, confirmation of response is not required since it will not add value to the interpretation of trial results. However, elimination of the requirement for response confirmation may increase the importance of central review to protect against bias, in particular in studies which are not blinded.

In the case of SD, measurements must have met the SD criteria at least once after study entry at a minimum interval (in general not less than 6~8 weeks) that is defined in the study protocol.

4.6.2. Duration of Overall Response

The duration of overall response is measured from the time measurement criteria are first met for CR/PR (whichever is first recorded) until the first date that recurrent or progressive disease is objectively documented (taking as reference for progressive

disease the smallest measurements recorded on study). The duration of overall complete response is measured from the time measurement criteria are first met for CR until the first date that recurrent disease is objectively documented.

4.6.3. Duration of Stable Disease

Stable disease is measured from the start of the treatment (in randomized trials, from date of randomization) until the criteria for progression are met, taking as reference the smallest sum on study (if the baseline sum is the smallest, this is the reference for calculation of PD). The clinical relevance of the duration of stable disease varies in different studies and diseases. If the proportion of patients achieving stable disease for a minimum period of time is an endpoint of importance in a particular trial, the protocol should specify the minimal time interval required between two measurements for determination of stable disease.

Note: The duration of response and stable disease as well as the progression-free survival are influenced by the frequency of follow-up after baseline evaluation. It is not in the scope of this guideline to define a standard follow-up frequency. The frequency should take into account many parameters including disease types and stages, treatment periodicity and standard practice. However, these limitations of the precision of the measured endpoint should be taken into account if comparisons between trials are to be made.

4.7. Progression-free Survival/Proportion Progression-free

4.7.1. Phase II trials

This guideline is focused primarily on the use of objective response endpoints for phase II trials. In some circumstances, response rate' may not be the optimal method to assess the potential anticancer activity of new agents/regimens. In such cases 'progression-free survival' (PFS) or the 'proportion progression-free' at landmark time points, might be considered appropriate alternatives to provide an initial signal of biologic effect of new agents. It is clear, however, that in an uncontrolled trial, these measures are subject to criticism since an apparently promising observation may be related to biological factors such as patient selection and not the impact of the intervention. Thus, phase II screening trials utilizing these endpoints are best designed

with a randomized control. Exceptions may exist where the behaviour patterns of certain cancers are so consistent (and usually consistently poor), that a non-randomized trial is justifiable. However, in these cases it will be essential to document with care the basis for estimating the expected PFS or proportion progression-free in the absence of a treatment effect.

For the endpoints evaluation, independent assessment and result reporting concerning the Phase III trials, please see the English version.

Appendix 3. Restricted and Prohibited Drugs and Food

Restricted drugs and prohibited traditional Chinese medicine and food during the study are as follows:

1. Strong inhibitors and strong inducers of CYP3A4

The comparatively more common strong inhibitors of CYPA34 are listed as follows, which include but not limited to:

Ketoconazole
Itraconazole
Fluconazole
Voriconazole
Nefazodone
Indinavir
Nelfinavir
Saquinavir
Erythromycin
Clarithromycin
Telithromycin
Grapefruit
Grapefruit juice

The comparatively more common strong inducers of CYPA34 are listed as follows, which include but not limited to:

Rifabutin
Rifampicin
Rifapentine
Carbamazepine
Phenobarbital
Phenytoin
Hypericum perforatum

2. Traditional Chinese medicine: include all traditional Chinese medicine for anti-tumor indications

Huazhenghuisheng Pian
Yadanziyou Ruanjiaonang
Zhemu Tangjiang
Ban'ao
Cinobufotalin
Kang'ai Injection
Fukang Capsules
Xiaoaiping
Pingxiao Capsules
Pingxiao
Shendan Sanjie Capsule
Kanglixin Capsules

Kanglaite	Ankangxin Capsules
Zhongjiefeng Injection	Bosheng Aining
Brucea javanica oil emulsion Injection	Zedoray Turmeric Oil and Glucose Injection
Aidi injection	Kanglixin Capsules
Awei Huapigao	Cidan Capsules
Kang'aiping Wan	

3. Food: Avoid eating fruits including grapefruit, pomelo or citrus maxima or any beverage containing the above-mentioned fruits.

Appendix 4. Safety Data Report

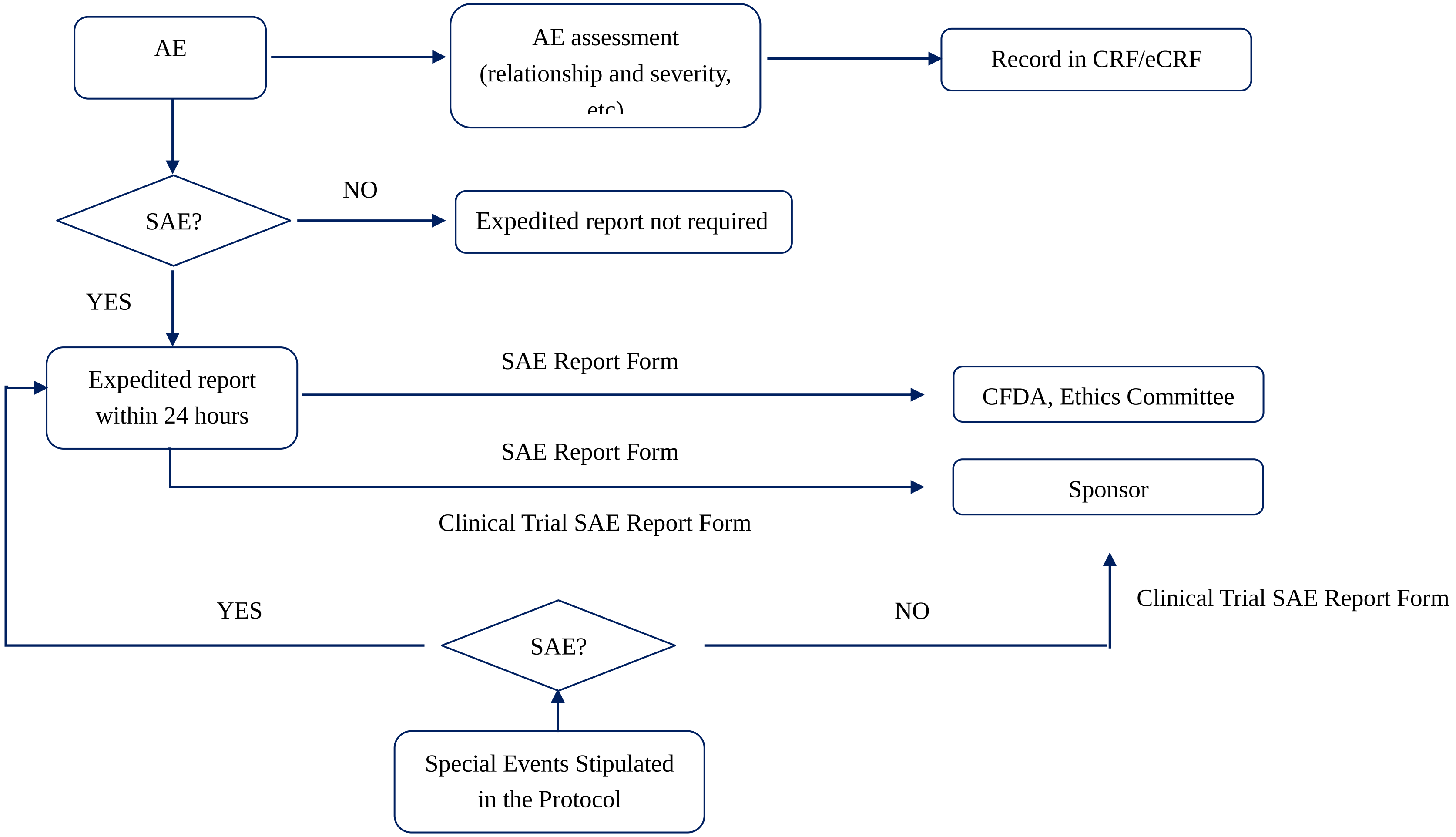

Appendix 5 Clinical Evaluation of Liver Damage

In accordance with the description of Section 7.1.3, for subjects with confirmed elevation of ALT/AST combined with increased total bilirubin (TBiL), which is equivalent to special event stipulated by the Protocol, repetitive blood biochemistry tests and increased frequency of monitoring are required to further describe the trend of biochemical indicators. In addition, it is necessary for investigators to exclude other causes leading to parameter abnormality through inquiry of medical history, physical examination and appropriate auxiliary examination.

Usual causes that may result in liver damage:

- Acute viral hepatitis
- Alcoholic and autoimmune hepatitis
- Biliary tract disease
- Cardiovascular reasons

Other less common causes may require consideration as well.

The investigator is recommended to obtain the following information, further evaluate and follow-up to complete the clinical data:

◆ Obtain the medical history of the subject

✧ Detailed history of current symptom, diagnosis of complications and medical history.

✧ Previous medical history (viral hepatitis, alcoholic hepatitis, autoimmune disease, biliary tract disease and cardiovascular diseases, etc.)

✧ History of concomitant medication (including OTC and prescription drugs, herbal medicine and dietary supplements), alcohol consumption, recreational drugs and special diet.

✧ History of exposure to chemicals

◆ Complete the following laboratory tests:

  ✧ Haematology

  ✧ Clinical biochemistry:

ALT, AST, bilirubin (including total bilirubin and direct bilirubin), alkaline phosphatase, albumin, PT or INR, amylase, fasting blood glucose, cholesterol and triglycerides.

- ✧ Serum test:

  Hepatitis A (Anti-IgM, Anti-IgG), hepatitis B (HbsAg, Anti-HBs and DNA), hepatitis C (Anti-HCV, and RNA test is required if positive), hepatitis D (Anti-IgM, Anti -IgG), hepatitis E (Anti-HEV, Anti-HEV IgM), and anti-nuclear antibody.

- ◆ Complete appropriate auxiliary examination:
- ✧ Subjects with confirmed elevation of ALT/AST combined with TBil are required to perform abdominal ultrasonography or other clinically applicable imaging examination within 48 hours (to exclude biliary tract, pancreas or intrahepatic causes, such as biliary calculi or tumor) and obtain the liver imaging result as soon as possible. If the causes (such as biliary tract, pancreas or intrahepatic causes) of abnormal hepatic result cannot be confirmed by imaging, paracentesis is recommended for pathological examination on the premise of obtaining consent of the subject;
- ✧ If suspected cardiovascular causes exist, cardiac ultrasonography is recommended to exclude cardiovascular dysfunction (including right heart failure, etc.);

Long-term follow-up: Perform close monitoring to the subject through repetitive tests of ALT, AST and bilirubin (including total bilirubin and direct bilirubin) for at least once a week till laboratory ALT and/or AST abnormally becomes stable or recovers, and then proceed according to the protocol. Report the data through the eCRF.
