# Supplementary figures and images for: Safety and efficacy of fruquintinib in patients with previously treated metastatic colorectal cancer: a phase Ib study and a randomized double-blind phase II study
Source: J Hematol Oncol. 2017 Jan 19;10:22. doi: 10.1186/s13045-016-0384-9 (PMC5244709; doi:10.1186/s13045-016-0384-9)

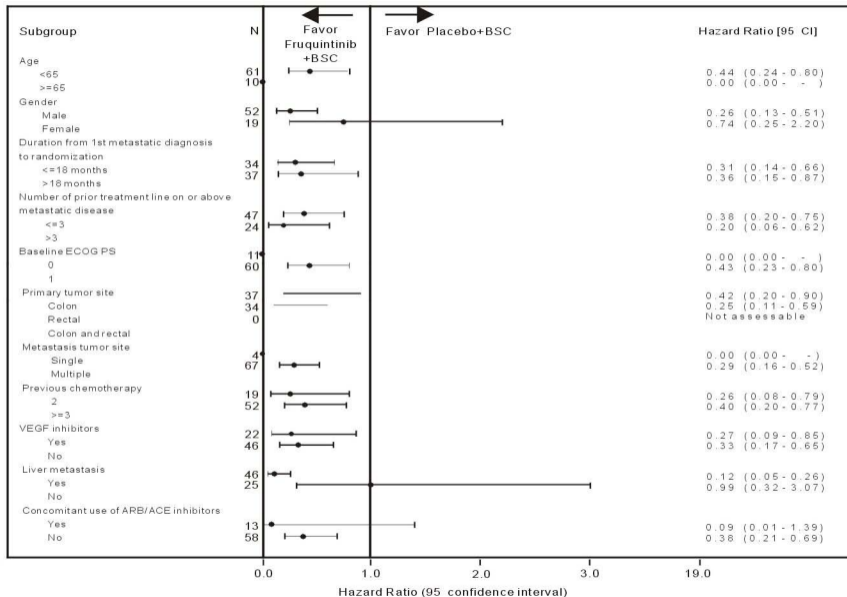

Supplement: Additional file 3: — Forest plot of subgroup analysis in phase II study. (PDF 104 kb) [file 13045_2016_384_MOESM3_ESM.pdf]

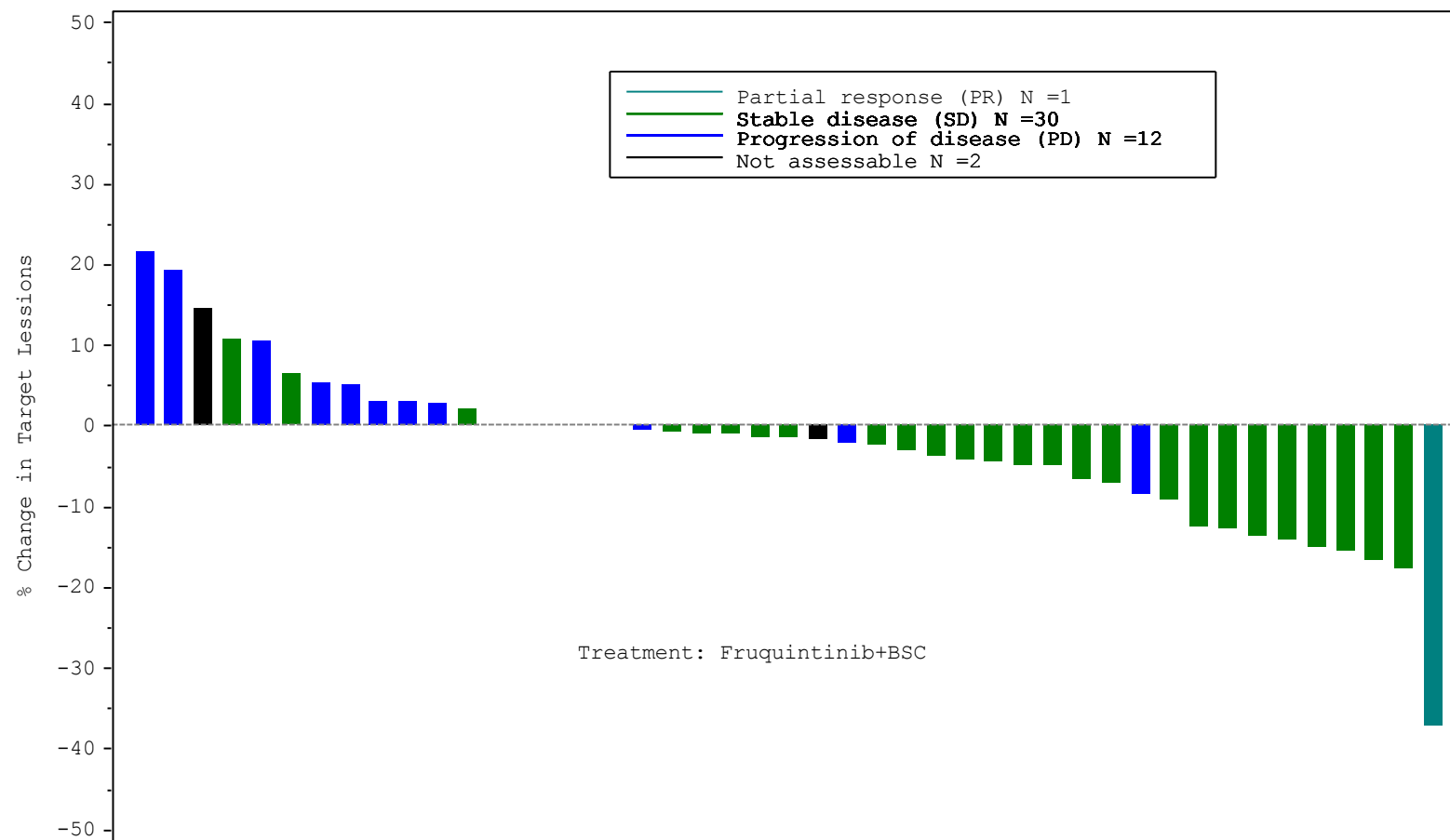

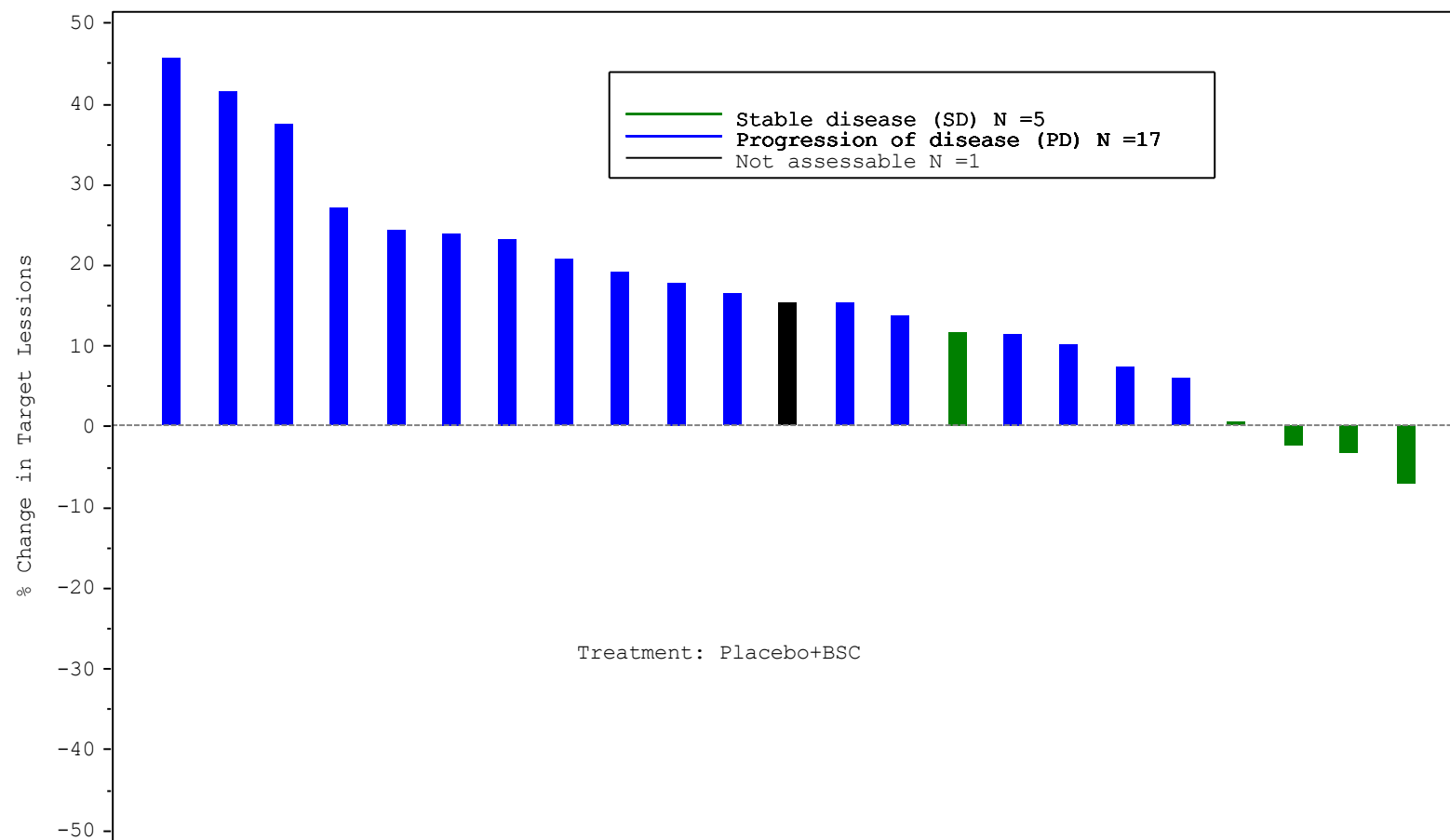

Supplement: Additional file 4: — The waterfall plots for tumor responses in phase II study. (PDF 49 kb) [file 13045_2016_384_MOESM4_ESM.pdf]
