# Supplementary material for: Safety and efficacy of fruquintinib in patients with previously treated metastatic colorectal cancer: a phase Ib study and a randomized double-blind phase II study
Source: J Hematol Oncol. 2017 Jan 19;10:22. doi: 10.1186/s13045-016-0384-9 (PMC5244709; doi:10.1186/s13045-016-0384-9)
Supplement: Additional file 5: — Analysis of progression-free survival in phase II study by blinded independent central review. (PDF 129 kb) [file 13045_2016_384_MOESM5_ESM.pdf]

Table 14.4.3
Analysis of Progression Free Survival (BICR)
ITT Set

Characteristic	Statistic	Fruquintinib+BSC (N=47)	Placebo+BSC (N=24)
NUMBER OF SUBJECTS WITH PROGRESSION OR DEATH	n (%)	44 (93.6)	23 (95.8)
PROGRESSION	n (%)	35 (74.5)	21 (87.5)
DEATH	n (%)	9 (19.1)	2 (8.3)
NUMBER OF SUBJECTS CENSORED	n (%)	3 (6.4)	1 (4.2)
REASON FOR CENSORING			
NO PD/DEATH	n (%)	3 (6.4)	1 (4.2)
RANDOMIZED BUT NO DRUG ADMINISTRATION	n (%)	0	0
NO POST BASELINE TUMOR ASSESSMENT	n (%)	0	0
PROGRESSION FREE SURVIVAL (MONTHS)	Median (95% CI)	3.713 (1.971, 4.731)	0.953 (0.920, 1.018)
	Stratified Log-Rank Test P-value	<0.001	
	Non-stratified Log-Rank Test P-value	0.002	
	Stratified HR (95% CI)	0.288 (0.150, 0.552)	
	Non-stratified HR (95% CI)	0.437 (0.260, 0.734)	

SOURCE: Listing 16.2.37
BICR: Blinded Independent Central Review. HR: Hazard Ratio.
% = Percentage of subjects in each category relative to the total number of subjects in the relevant analysis set.
Median progression free survival and survival rate are based on Kaplan-Meier estimate.
P-values are calculated from stratified/non-stratified log-rank tests (Fruquintinib+BSC vs. Placebo+BSC).
HRs and its 95% CIs are calculated from stratified/non-stratified cox proportional hazard models. Stratification factors are previous chemotherapy, VEGF inhibitors and liver metastasis. If less than 5 subjects in one stratification level, this stratum will be removed from stratified model. Subjects 10011001, 10061001 and 10061008 are excluded from stratified analysis due to the uncertainty of VEGF inhibitor usage.
For subjects without PD or death, the date of last tumor assessment is the censoring date. For subjects without death and tumor assessment after baseline evaluation, censoring date is the randomization date.

Table 14.4.3
Analysis of Progression Free Survival (BICR)
ITT Set

Characteristic	Statistic	Fruquintinib+BSC (N=47)	Placebo+BSC (N=24)
SURVIVAL RATE AT			
3 MONTHS		0.506	0.125
6 MONTHS		0.132	0.083

SOURCE: Listing 16.2.37
BICR: Blinded Independent Central Review. HR: Hazard Ratio.
% = Percentage of subjects in each category relative to the total number of subjects in the relevant analysis set.
Median progression free survival and survival rate are based on Kaplan-Meier estimate.
P-values are calculated from stratified/non-stratified log-rank tests (Fruquintinib+BSC vs. Placebo+BSC).
HRs and its 95% CIs are calculated from stratified/non-stratified cox proportional hazard models. Stratification factors are previous chemotherapy, VEGF inhibitors and liver metastasis. If less than 5 subjects in one stratification level, this stratum will be removed from stratified model. Subjects 10011001, 10061001 and 10061008 are excluded from stratified analysis due to the uncertainty of VEGF inhibitor usage.
For subjects without PD or death, the date of last tumor assessment is the censoring date. For subjects without death and tumor assessment after baseline evaluation, censoring date is the randomization date.

K:\Hutchison\Fruquintinib\UWA13414\Biostatistics\Production\Tables\T_14.4-3.sas 23DEC2015 11:42

Table 14.4.4
Summary of Early and Late Discordance Rates between BICR and LE
ITT Set

Characteristic	Statistic	Fruquintinib+BSC BICR Assessment PD	Fruquintinib+BSC BICR Assessment NO PD	Placebo+BSC BICR Assessment PD	Placebo+BSC BICR Assessment NO PD
LE ASSESSMENT					
PD	N	(a1/a2/a3) 5/27/0	(b) 7	(a1/a2/a3) 10/8/0	(b) 1
NO PD	N	(c) 3	(d) 5	(c) 3	(d) 2
EARLY DISCREPANCY RATE (EDR)		0.18		0.05	
LATE DISCREPANCY RATE (LDR)		0.81		0.92	

SOURCE: Listing 16.2.20 and 16.2.37

BICR: Blinded Independent Central Review. LE: Local Evaluation.
a1: Number of agreements on timing and occurrence of PD. a2: Number of times LE declares PD later than BICR. a3: Number of times LE declares PD earlier than BICR.
a=a1+a2+a3. EDR=(b+a3)/(a+b). LDR=(c+a2)/(b+c+a2+a3).

K:\Hutchison\Fruquintinib\UWA13414\Biostatistics\Production\Tables\T_14.4-4.sas 23DEC2015 11:42

Hutchison 2012-013-00CH1 - Cutoff Date: 04Nov2015 - Final - Unblinded

Figure 14.4.3
Kaplan-Meier Curve of Progression Free Survival by Treatment Group (BICR)
ITT Set

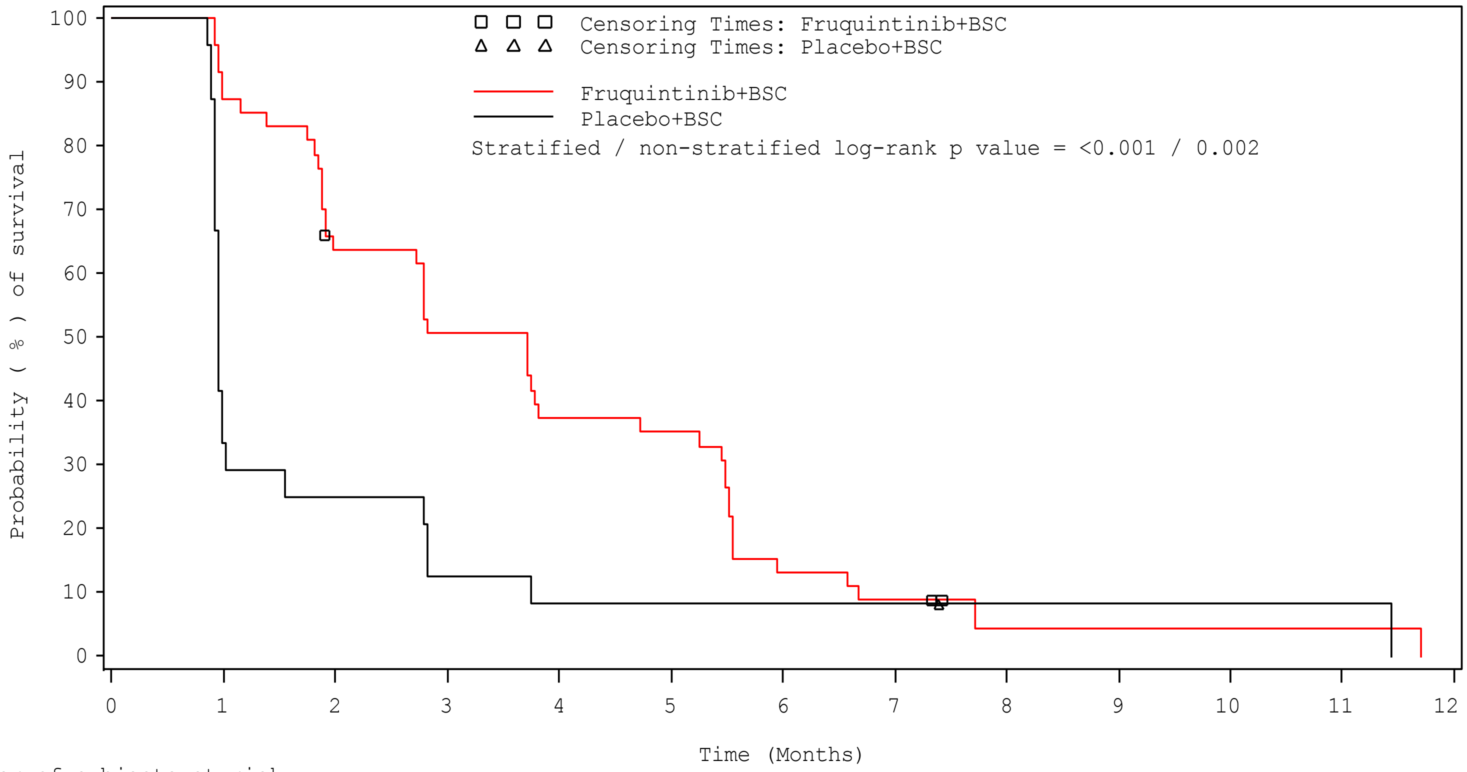

Number of subjects at risk

**Fruquintinib+BSC**	47	41	29	23	17	16	6	4	1	1	1	1	0
**Placebo+BSC**	24	8	6	3	2	2	2	2	1	1	1	1	0

- P-value is obtained from stratified/non-stratified log-rank test. If less than 5 subjects in one stratification level, this strata will be removed from stratified model. Subjects 10011001, 10061001 and 10061008 are excluded from stratified analysis due to the uncertainty of VEGF inhibitor usage, but are included in the non-stratified analysis and Kaplan-Meier curve.

SOURCE: Listing 16.2.37

K:\Hutchison\Fruquintinib\UWA13414\Biostatistics\Production\Figures\F_14.4-3.sas 23DEC2015 11:47
